# Emerging Status of Multidrug-Resistant Bacteria and Fungi in the Arabian Peninsula

**DOI:** 10.3390/biology10111144

**Published:** 2021-11-06

**Authors:** J. Francis Borgio, Alia Saeed Rasdan, Bayan Sonbol, Galyah Alhamid, Noor B. Almandil, Sayed AbdulAzeez

**Affiliations:** 1 Department of Epidemic Diseases Research, Institute for Research and Medical Consultations (IRMC), Imam Abdulrahman Bin Faisal University, Dammam 31441, Saudi Arabia; 2210500048@iau.edu.sa (A.S.R.); 2210500110@iau.edu.sa (B.S.); 2210500121@iau.edu.sa (G.A.); 2 Department of Genetic Research, Institute for Research and Medical Consultations (IRMC), Imam Abdulrahman Bin Faisal University, Dammam 31441, Saudi Arabia; asayed@iau.edu.sa; 3 Department of Clinical Pharmacy Research, Institute for Research and Medical Consultations (IRMC), Imam Abdulrahman Bin Faisal University, Dammam 31441, Saudi Arabia; nbalmandil@iau.edu.sa

**Keywords:** multidrug-resistant bacteria, *Acinetobacter baumannii*, *Mycobacterium tuberculosis*, mortality, *Candida auris*, Arabian Peninsula

## Abstract

**Simple Summary:**

The incidence and developing status of multidrug-resistant bacteria and fungi, as well as their related mortality, is reviewed by a systematic published literature search from nine countries in the Arabian Peninsula. In order to analyse the emerging status and mortality, a total of 382 research articles were selected from a comprehensive screening of 1705 papers. More than 850 deaths reported since 2010 in the Arabian Peninsula due to the infection of multidrug-resistant bacteria and fungi. Multidrug-resistant bacteria *Acinetobacter baumannii, Mycobacterium tuberculosis*, *Staphylococcus aureus*, and fungi *Candida auris* are the most prevalent and causing high deaths. To control these infections and associated deaths in the Arabian Peninsula, continuous preventive measures, accurate methods for early diagnosis of infection, active surveillance, constant monitoring, developing vaccines, eradicating multidrug resistance modulators, and data sharing among countries are required.

**Abstract:**

We aimed to identify the prevalence and emerging status of multidrug-resistant bacteria and fungi and their associated mortality in nine countries in the Arabian Peninsula. Original research articles and case studies regarding multidrug-resistant bacteria and fungi in the Arabian Peninsula, published during the last 10 years, were retrieved from PubMed and Scopus. A total of 382 studies were included as per the inclusion and exclusion criteria, as well as the PRISMA guidelines, from a thorough screening of 1705 articles, in order to analyse the emerging status and mortality. The emerging nature of >120 multidrug-resistant (MDR) bacteria and fungi in the Arabian Peninsula is a serious concern that requires continuous monitoring and immediate preventive measures. More than 50% (*n* = 453) of multidrug-resistant, microbe-associated mortality (*n* = 871) in the Arabian Peninsula was due to MDR *Acinetobacter baumannii, Mycobacterium tuberculosis* and *Staphylococcus aureus* infection. Overall, a 16.51% mortality was reported among MDR-infected patients in the Arabian Peninsula from the 382 articles of this registered systematic review. MDR *A. baumannii* (5600 isolates) prevailed in all the nine countries of the Arabian Peninsula and was one of the fastest emerging MDR bacteria with the highest mortality (*n* = 210). A total of 13,087 *Mycobacterium tuberculosis* isolates were reported in the region. *Candida auris* (580 strains) is the most prevalent among the MDR fungal pathogen in the Arabian Peninsula, having caused 54 mortalities. Active surveillance, constant monitoring, the development of a candidate vaccine, an early diagnosis of MDR infection, the elimination of multidrug resistance modulators and uninterrupted preventive measures with enhanced data sharing are mandatory to control MDR infection and associated diseases of the Arabian Peninsula. Accurate and rapid detection methods are needed to differentiate MDR strain from other strains of the species. This review summarises the logical relation, prevalence, emerging status and associated mortality of MDR microbes in the Arabian Peninsula.

## 1. Purpose and Methods

This systematic review of the emerging status of multidrug-resistant (MDR) bacteria and fungi in the Arabian Peninsula was designed to answer the following questions:

1. Is there a common MDR organism (MDRO) reported in the Arabian Peninsula?
2. Are there any MDR bacteria and fungi that have emerged in the Arabian Peninsula in the last 10 years?
3. What are the logical relationships between sets of MDR bacteria and fungi in countries of the Arabian Peninsula?
4. How many in-depth studies have been conducted on the molecular nature of drug-resistant micro-organisms prevalent in the Arabian Peninsula?
5. What are the novel antimicrobial strategies developed in the study region?
6. Is there a high mortality reported due to MDR micro-organisms in the Arabian Peninsula?
7. To what extent have nanomaterials and nanoparticles been exploited against MDR micro-organisms in the Arabian Peninsula?

This review aims to identify the prevalence and emerging status of multidrug-resistant bacteria and fungi and their associated mortality in nine countries of the Arabian Peninsula: Saudi Arabia, Bahrain, Kuwait, Oman, Qatar, the United Arab Emirates, Jordan, Iraq and Yemen. Published data from the past 10 years were retrieved from PubMed, Scopus and Google Scholar from 2 March 2021 to 8 March 2021. This review was registered with PROSPERO (CRD42021246777). The participants or populations selected from the Arabian Peninsula were Saudi Arabia, Bahrain, Kuwait, Oman, Qatar, the United Arab Emirates, Jordan, Iraq, and Yemen.

Inclusion criteria:

1. Articles reporting the multidrug-resistant bacteria in the Arabian Peninsula.
2. Articles reporting multidrug-resistant fungi in the Arabian Peninsula.
3. Research articles published since 2010 regarding multidrug-resistant micro-organisms in the Arabian Peninsula.
4. Full-text articles.

Exclusion criteria:

1. Articles discussing the use of multidrug-resistant bacteria and fungi to screen the antimicrobials in the Arabian Peninsula.
2. Articles reporting the multidrug-resistant micro-organisms from other than the Arabian Peninsula.
3. Review articles reporting the multidrug-resistant micro-organisms from the Arabian Peninsula.

**Figure 1:**
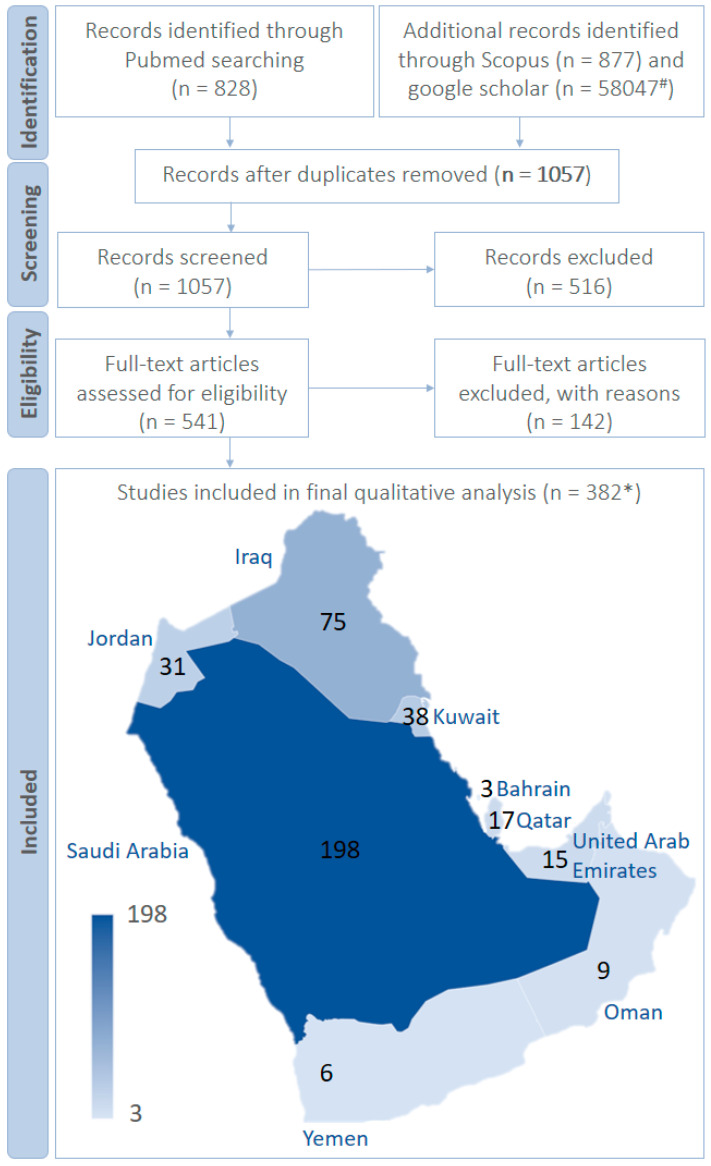
PRISMA 2009 flow diagram for the selection of articles reporting multi drug-resistant micro-organisms from the Arabian Peninsula, since 2010. * There were three papers reporting MDR micro-organisms in multiple countries [1]: Bahrain, Saudi Arabia and United Arab Emirates; [2]: Saudi Arabia, United Arab Emirates, Oman, Qatar, Bahrain, and Kuwait; [3]: Saudi Arabia, Kuwait, Oman and United Arab Emirates. ^#^ Google scholar not considered. Country-wise data for the number of articles from various databases are presented in Supplementary Table S1.

Original research articles and case studies of multidrug-resistant bacteria and fungi in the Arabian Peninsula and their associated mortality were considered as articles of interest. A total of 1705 articles (published after 2010) from Scopus and PubMed were thoroughly screened for eligibility, as per the inclusion and exclusion criteria. A total of 382 studies were eligible for inclusion as per PRISMA 2009 (Figure 1; Supplementary Table S1). There were three papers reporting MDR micro-organisms from multiple countries: Article 1 [1]—Bahrain, Saudi Arabia and United Arab Emirates; Article 2 [2]—Saudi Arabia, United Arab Emirates, Oman, Qatar, Bahrain, and Kuwait and Article 3 [3]—Saudi Arabia, Kuwait, Oman and United Arab Emirates. 

The most urgent public and animal health problems are increasingly caused by multidrug-resistant (MDR) micro-organisms. This problem is widely shown in bacteria that defy ultimate antibiotics and portend an untreatable future. The Arabian Peninsula has several challenges that stimulate the emergence and spread of multidrug-resistant bacteria. Dealing with these challenges requires the of multiple sectors to successfully control the spread and emergence of antimicrobial resistance. The present review summarises the logical relationship between emerging multidrug resistance organisms and associated mortality in the Arabian Peninsula.

## 2. Logical Relation between Sets of Multidrug-Resistant (MDR) Microbes

**Figure 2:**
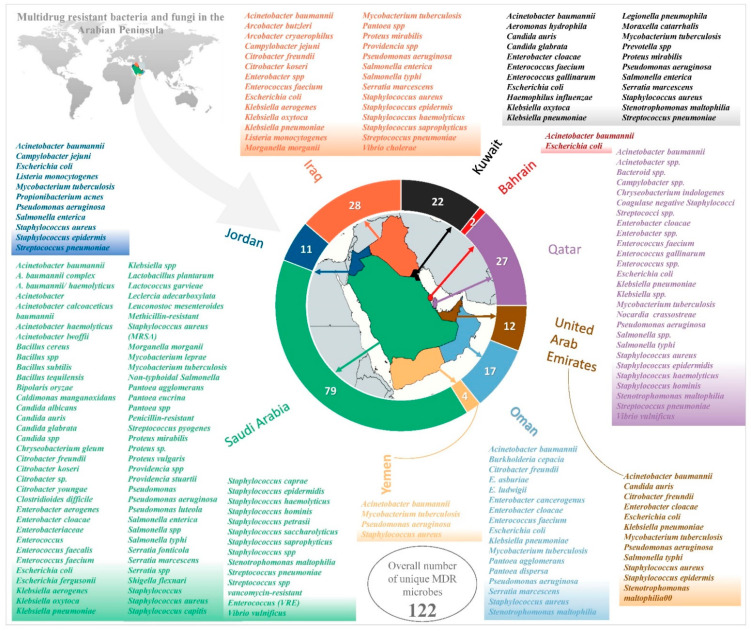
List of multidrug-resistant bacteria and fungi reported in Arabian Peninsula. Numbers in the doughnut indicate the number of multidrug-resistant organisms reported in the respective countries of the Arabian Peninsula. MRSA: Methicillin-resistant *Staphylococcus aureus*.

Eighty different species of multidrug-resistant bacteria and fungi were reported in the Arabian Peninsula (Figure 2). MDR *Acinetobacter baumannii* prevailed in all the nine countries of the Arabian Peninsula and was the most prevalent MDR microbe (Table 1), and was able to survive in hot dry summers and mild wet winters. After *A. baumannii, Escherichia coli* was the next most prevalent MDR microbe, reported in eight countries of the Arabian Peninsula; it was not reported in Yemen.

**Figure 3:**
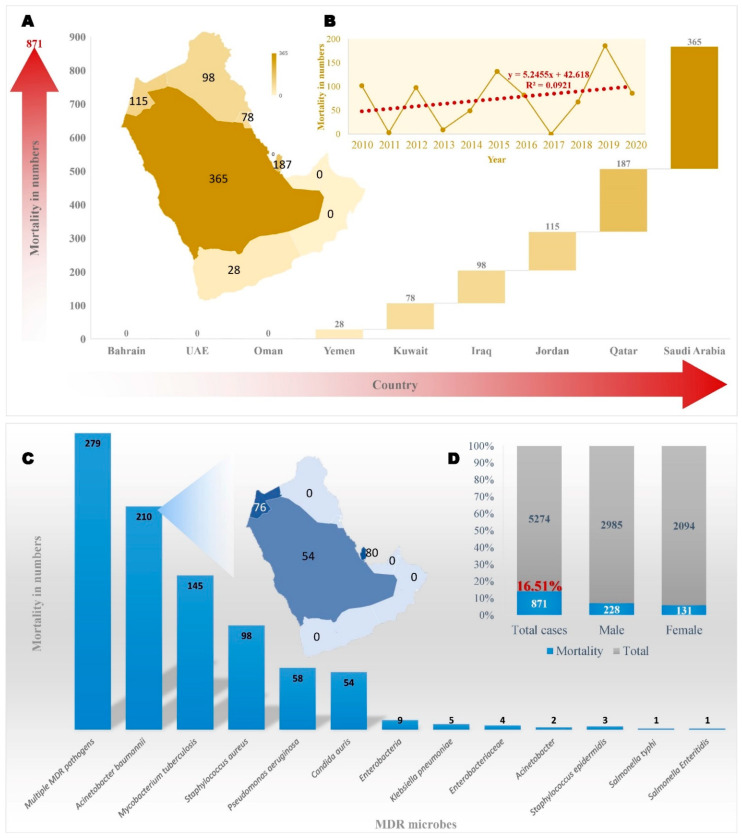
(**A**) Multidrug-resistant microbial associated mortality reported in the Arabian Peninsula. (**B**) Dark red line indicates linear trend line of annual mortality due to multidrug-resistant microbial infection. (**C**) Number of deaths reported due to specific MDR microbial infection in Arabian Peninsula. Map in blue color indicate the country wise mortality due to *Acinetobacter baumannii*. (**D**) Percentage mortality due to MDR microbes. Gender information were not reported on the mortality in certain articles. UAE: United Arab Emirates.

MDR *Candida auris* is a globally emerging fungal pathogen, for which diagnoses and treatments are challenging. The incidence of *C. auris* in clinical specimens from Kuwait, Saudi Arabia, and United Arab Emirates was reported in 11 articles; 580 strains (Table 1 and Table 2) responsible for 54 deaths (Figure 3, Table 3 and Table 4). A total of 13087 Mycobacterium tuberculosis isolates were reported in the region (Table 5).

### 2.1. Saudi Arabia

Due to its large area and population, the Kingdom of Saudi Arabia has the most significant number of scientific studies on the subject of MDR organisms among the countries of the Arabian Peninsula. Therefore, it has several obstacles that might promote the emergence and transmissiom of MDR micro-organisms. Eventually, a total of 79 unique MDR microbes were reported (Figure 2). The difficulties of this were overcome due to the successful efforts of different sectors to restrict the growth and establishment of MDR micro-organisms in the country. The active monitoring of the onset and spread of MDR micro-organisms is very important. Many relevant articles were published in research and case studies from Saudi Arabia on MDR bacteria and fungi. The inclusion criteria were applied carefully to all the search results (for articles published after 2010) from Scopus and PubMed. A total of 198 studies, as per PRISMA 2009, were eligible for inclusion (Figure 1; Supplementary Table S1). Three publications reported MDR micro-organisms in more than one country of the Arabian Peninsula: the report by Sonnevend and colleagues [1] in Bahrain, Saudi Arabia and United Arab Emirates; a study by Zowawi and colleagues [2] in Saudi Arabia, United Arab Emirates, Oman, Qatar, Bahrain, and Kuwait and a report by Sonnevend and colleagues [3] in Saudi Arabia, Kuwait, Oman and United Arab Emirates. These reports were considered separately for individual counties and also considered as only three studies in general (Figure 1). One hundred and ninety-eight studies reported various MDR organisms in Saudi Arabia, (Table 2), where there were considerably more MDR organisms compared to the other countries in the Arabian Peninsula.

Saudi Arabia had the highest number of MDR strains reported compared to other countries (Supplementary Table S2): 10972 *Escherichia coli*, 9829 *Mycobacterium tuberculosis*, 4092 *Klebsiella pneumonia*, 3787 *Acinetobacter baumannii*, 2594 *Pseudomonas aeruginosa*, 2014 *Acinetobacter*, 986 *Staphylococcus aureus*, 927 *Klebsiella* spp., 812 *Enterobacter*, 722 *Proteus vulgaris*, 715 *Enterobacteriaceae*, 695 *Methicillin-resistant Staphylococcus aureus (MRSA)* and 553 *Enterococcus*. Recently, the emergence of 18 *Clostridioides difficile and* 19 *Candida auris* were reported [24,317]. Between 2010 and 2021, the top five research papers reported MDR micro-organisms in Saudi Arabia. The first report included *A. baumannii* 59 isolates from human samples: urine, blood culture [147], respiratory specimens, skin and soft tissue specimens, blood, sterile body fluids [146,224] Biofilm-Formation in Clonally Unrelated Multidrug-Resistant Acinetobacter baumannii Isolates), wound swabs, rectal swabs, and sputum [326]. The second report included 51 *E. coli* isolates from human and animal samples, the isolates from human samples originated from tissue swabs [327] (the genomic diversity and antimicrobial resistance genes in multidrug-resistant CTX-M-positive isolates of Escherichia coli at a healthcare facility in Jeddah), urine, wound pus, vaginal swabs, blood culture, stools and ear swabs [87]. The isolates from animal samples originated from: chicken [60], camel [77], and minced meat samples [72]. The third report included 36 *K. pneumonia* isolates from human samples: blood, urine, wound swabs, sputum [40], rectal swabs and suction swabs [216]. The fourth report included 36 *P. aeruginosa* isolates from human samples: respiratory, urine, surgical, blood culture, genital swabs, eye swabs, ear swabs, burns [234], skin tissue, intra-abdominal fluid, bone tissue [26], wound swabs, sputum, and tracheal swabs [228]. The fifth report included 31 *M. tuberculosis* isolates from human samples: sputum, tissues and biopsies, abscesses, abdominal fluids, breast fluid, cerebrospinal fluid, urine, knee fluid and synovial fluid [117], wound swabs, ENT swabs, blood, tracheal swabs [130] (Supplementary Table S3).

Saudi Arabia had the highest mortality rate, with 365 patients who died due to MDR microbes (Figure 3A). The top three MDR organisms with the highest mortality rates were *A. baumannii*, *M. tuberculosis* and *P. aeruginosa.* Six MDR organisms were related to mortality: *S. epidermidis*, *A. baumannii*, *M. tuberculosis*, *C. auris*, *P. aeruginosa* and *Enterobacteriaceae* (Table 4). Thus, there were 365 MDR strains associated with death. *M. tuberculosis* caused the highest level of mortality [27]. The impact of the migrant workers from other countries cannot be understated; in 2017, a Filipino resident of Saudi Arabia was diagnosed with MDR *Mycobacterium leprae.* The etiologic agent was discovered using metagenomic sequencing a biopsy sample of the patient’s skin [324]. In one of the studies, 105 adult patients admitted to the intensive care unit (ICU) at Aseer Central Hospital in 2014 and 2015 were reviewed retrospectively. The species of *Acinetobacter* were isolated using specific phenotypes and verified by the automated system, Vitek 2. Of the 105 stains, genus *Acinetobacter* accounted for *A. baumannii* 49, *A. baumannii* complex 19, *A. baumannii/haemolyticus* 32, *A. haemolyticus* 4, *A. lwoffii* 1 [144]. There are well-established studies on electron microscopic structures, which reported both dead and alive multidrug-resistant micro-organisms in Saudi Arabia, using confocal laser scanning micrography [328]. 

### 2.2. Bahrain

The Kingdom of Bahrain had the lowest number of research articles, in contrast to the rest of the Arabian Peninsula’s countries, due to its small area and population. The papers in this review, published between 2010 and 2021 and retrieved from PubMed and Scopus, were thoroughly screened for their eligibility, as per the inclusion and exclusion criteria. Only three studies published on MDR bacteria were included in the final qualitative analysis, as per PRISMA 2009 (Figure 1; Supplementary Table S1). Two studies reported MDR micro-organisms in multiple countries: a study by Sonnevend and colleagues [1] in Bahrain, Saudi Arabia, and the United Arab Emirates; and a study by Zowawi and colleagues [2] in Saudi Arabia, United Arab Emirates, Oman, Qatar, Bahrain, and Kuwait (Figure 1). Two unique MDR microbes were reported: *Acinetobacter baumannii* and *Escherichia coli* (Figure 2). Compared to the other countries mentioned in this review, Bahrain had the lowest reported MDR strain (*n* = 11): eight *A. baumannii* and three *E. coli* (Supplementary Table S2). Three *E. coli* isolates from human specimens were obtained, including urine and tissue swabs [1], and eight *A. baumannii* isolates, from which human samples of blood, sputum, swab, and urine were collected [2] (Supplementary Table S3). Unlike the mortality rates of the other countries, Bahrain reported no MDR microbial infection-associated deaths (Figure 3A). In one of the scientific articles, researchers screened for the *mcr-1* gene of colistin resistant *Enterobacteriaceae* in Bahrain; the *mcr-1* strains were positive and their antibiotic resistance was determined. The *mcr-1* gene was found in two *E. coli* isolates [1]. In the study by Thani, on a urinary tract infection (UTI) cefotaxime resistant *E. coli* strain, a new DNA fragment was discovered. The DNA sequence was found to be linked to the pathogenicity island III_536_ locus. Researchers used the Single Genome Specific Primer-PCR (SGSP-PCR) to study the genomic state of the newly identified locus, which was discovered to obtain the antibiotic resistance gene bla_CTX m_ [110]. Bahrain was included in the research on the molecular epidemiology and resistance mechanisms of carbapenem-resistant acinophilic bacteria (CRAB) for the Arabian Peninsula countries; eight *A. baumannii* isolates were identified in Bahrain hospitals, and OXA-51 was found in all of the isolates. Antibiotic resistance genes were detected using PCR, and clonality was determined using repetitive sequence-based PCR (rep-PCR). The majority (84%) of the isolates in this investigation were related to health exposure. The increased awareness of multidrug-resistant organisms in the Gulf Cooperation Council (GCC) states has significant implications for improving infection control procedures, developing antimicrobial stewardship programs and highlighting the importance of regional active monitoring systems [2].

### 2.3. Kuwait

The studies, published between 2010 and 2021, from selected databases were evaluated for eligibility, as per the inclusion and exclusion criteria, according to PRISMA 2009. Thirty-eight papers on multidrug-resistant (MDR) micro-organisms were included in the final qualitative analysis (Figure 1; Supplementary Table S1). A total of 22 unique MDR micro-organisms were identified from Kuwait (Figure 2), including the studies that reported MDR micro-organisms in various countries (Figure 1) [2,3]. There were seven publications that reported >100 MDR strains such as: 7138 methicillin-resistant *Staphylococcus aureus* (MRSA), 1176 *Mycobacterium tuberculosis*, 561 *Candida auris*, 410 *Acinetobacter baumannii*, 302 *Staphylococcus aureus*, 222 *Klebsiella pneumonia* and 149 *Enterobacteriaceae* (Supplementary Table S2). Notably, five studies in Kuwait from 2010 to 2021 revealed MDR micro-organisms. The first study revealed 11 *M. tuberculosis*, from human samples such as, sputum, pus, tissue biopsy, lymph node and endotracheal secretion, and cerebrospinal [112] samples. The second study found eight isolates of *A. baumannii* acquired from human urine [198] and swab specimens, sputum, blood [2]. The third study discovered six *C. auris* human isolates from axilla, groin, anterior nares, vascularline exit sites, the oropharynx, respiratory and urinary tract. The isolates were also taken from environmental samples: rooms, units occupied by all the infected *C. auris*, colonised patients, medical devices, linen, walls, floors, furniture, doorknobs, bed railings, bedside drawers, toilet faucets, and flush handles [9]. The fourth study observed five isolates of *Enterobacteriaceae* obtained from blood, urine, wound, and tissue swabs [259]. Finally, the fifth study anlysed four *E. coli* isolates of human samples derived from blood culture, urine, wounds, and central venous pressure [10]. After Saudi Arabia, Qatar, Jordan, and Iraq, Kuwait has the fifth highest mortality rate, with a total number of 78 non-survivors associated with MDR microbial infection (Figure 3A). Seven articles reported MDR microbial infection associated with mortality. The highest mortality in Kuwait was related to the MDR organism, *C. auris,* with 37 non-survivor patients, whereas *C. auris* were the most prominent MDRO in terms of the number of deaths (Table 3). 

Healthcare-related pneumonia caused by MDR pathogens poses a significant treatment challenge. The frequency of antibiotic medication leads to inefficient antimicrobial therapy. Jamal and colleagues reported unique MDR organisms: two isolates of *Haemophilus influenzae*, two isolates of *Legionella pneumophila*, and one isolate of *Moraxella catarrhalis*. Critically ill patients with clinically diagnosed respiratory tract infections, hospitalised at Mubarak Al Kabeer Hospital between January and April 2013, were selected for their study. Recently, a rapid-multiplex, PCR-based Unyvero pneumonia application (UPA) assay that aids in early decision making became accessible. The respiratory samples from patients with nosocomial pneumonia included the sputum, bronchoalveolar lavage fluid, and endotracheal secretions [12]. From January to December 2016, a study conducted by Hamza and colleagues revealed a novel pathogen of an MDR micro-organism. All adult patients who had the specified GI procedures, used to estimate surgical site infection (SSI) rates among gastrointestinal surgeries in all Kuwait governmental hospitals, were examined in this study. Eventually, a single isolate of *Aeromonas hydrophila* was obtained from small bowel (SB) surgery [329].

### 2.4. Oman

After removing duplications, peer reviewed articles, and non-relevant countries of interest, a total of 42 articles were screened. Thirty-three studies were excluded from further analyses and nine articles fulfilled the inclusion criteria from the published data for MDR organisms from Oman in PubMed (*n* = 43) and Scopus (*n* = 36). Seventeen different species of multi-drug-resistant organisms were reported in Oman (Figure 2). *Escherichia coli* was the most recorded MDRO; this organism was identified five times in the studies (Table 2). Although several MDRO were recorded in this country, the following organisms were reported as the lowest among the collected studies: *Pseudomonas aeruginosa, Staphylococcus aureus, Stenotrophomonas maltophilia Citrobacter freundii, Enterobacter asburiae, Enterobacter hormaechei, and Enterobacter ludwigii, Serratia marcescens and Pantoea dispersa*. The total number of MDRO strains reported in Oman were as follows: 520 *Klebsiella pneumoniae*, 224 *Acinetobacter baumannii*, 78 *Escherichia coli,* 35 *Staphylococcus aureus*, 27 *Pseudomonas aeruginosa*, 25 *Stenotrophomonas maltophilia*, 8 *Enterobacter cloacae*, 2 *Enterococcus faecium,* and 1 *Citrobacter freundii*, (Supplement Table S2). 

In the Oman region, *E. coli* was the most abundant MDRO, as reported by several studies. The study by Al-Kharousi focused on the microbial features of fresh fruit and vegetables at the market stage, evaluating the impact of antibiotic-resistant enterobacteria transmission. More specifically, this study focused on one of the resistance mechanisms in Enterobacteriaceae that could produce AmpC β-lactamase in cefoxitin-resistant isolates. Fourteen isolates displayed a multi-drug resistance for different classes of enterobacteria. From these different classes, *E. coli* isolates gained resistance to several types of antibiotics compared to other classes; they displayed the resistance to five antibiotics such as ampicillin, chloramphenicol, kanamycin, nalidixic acid, and tetracycline. Six *E. coli* isolates were collected from lettuce samples that gained a resistance to multiple antibiotics. Some isolates were phenotypically positive for producing the ampC β-lactamase [257]. Another study focused on the epidemiology of multi-drug-resistant organisms via the site of infection and types of bacteria at Sultan Qaboos University Hospital. Although several MDRO were identified, *E. coli* was the second most abundant among the isolates. The number of isolates was 60 for *E. coli* with extended spectrum β-lactamases and 5 for *E. coli* with a carbapenem resistance, respectively, among the samples from different infection sites, such as the bloodstream, urinary tract, and surgical infection [330]. A study focused on the colistin-resistant Enterobacteriaceae in several countries of the Arabian Peninsula, more specifically, studying the presence of the mcr-1 gene in this class. Four *E. coli* isolates were MDROs had an mcr-1-positive strain. The *E. coli* strains such as ST648, ST224, ST68, and ST131 belonged to other countries; none of these strains were from Oman [1]. The focus on identifying Arabian Enterobacteriaceae in the Arabian Peninsula region revealed the presence of strains with New Delhi metallo-beta lactamase (NDM)-7. Four stains showed a multi-drug resistance to NDM-7 *E. coli*; only one (*E. coli* ST4107) strain was from Oman Hospital, isolated from a patient’s endotracheal secretion. The blaNDM7 was identified on IncX3-type plasmids. In this study, an association was found between identifying the IncX3 type plasmids and spreading the rare NDM-7 variant in the region [101]. The defined mortality data associated with MDRO was not declared in studies from the last ten years [331,332]. This could be a major limitation of the statistical mortality data from the region; further studies are needed to discover the mortality data, which can be correlated with MDRO. Although several MDROs recorded in Oman were found in other regions in the Arabian Peninsula, few organisms were only found in the Oman region: *Pantoea dispersa, Bukholderia cepacian, Enterobacter Hormaechei*, and *Enterobacter ludwigii* (Table 1). Therefore, these MDROs were considered unique to the Oman region since they were able to survive in this region; specific studies are needed to find their prevalence and associated physiology.

### 2.5. Qatar

The searching process for the published data of MDRO from Qatar for the last decade resulted in a similar number of research results for both PubMed and Scopus; 54 and 52 studies were found in PubMed and Scopus, respectively. After removing duplicates, peer reviewed articles, and non-relevant countries of interest, a total of 52 articles were screened. Thirty-five were excluded from further analyses. Seventeen articles were screened for full-test article eligibility by screening the title and the abstract (Supplementary Table S1). Twenty-seven different species of multidrug-resistant organisms were reported in Qatar (Figure 2). *Escherichia coli* was the most recorded MDRO and was identified six times (Table 2). Among the collected studies, there were several MDRO recorded in Qatar; nevertheless, the following organisms were recorded as the lowest: *Mycobacterium tuberculosis, Staphylococcus aureus, Streptococcus pneumoniae, Stenotrophomonas maltophilia, Enterobacter cloacae, Salmonella typhi, Vibrio vulnificu, Campylobacter* spp., *Nocardia crassostreae, Staphylococcus epidermidis, Staphylococcus haemolyticus, Staphylococcus hominis, Enterococcus gallinarum, Chryseobacterium indologenes, Acinetobacter* spp., *Enterobacter* spp., *Klebsiella* spp., *Salmonella* spp., *Bacteroid* spp., *Coagulase negative Staphylococci, Streptococci* spp., and *Enterococcus* spp. These organisms were reported once as MDR (Table 2). A total of five MDRO above 100 strains were reported in Qatar: 426 *Acinetobacter baumannii*, 223 *Escherichia coli*, 223 *Mycobacterium tuberculosis*, 178 *Pseudomonas aeruginosa*, and 134 *Streptococcus pneumoniae*. Furthermore, six MDR organisms were reported: 73 *Campylobacter jejuni*, 34 *Staphylococcus aureus*, 26 *Staphylococcus epidermidis*, 24 *Klebsiella pneumoniae*, 23 *Vibrio vulnificus* and 13 *Nocardia crassostreae* (Supplementary Table S2).

Overall, *Escherichia coli* was the most abundant MDRO in Qatar. From the studies in Qatar, six research articles reported *Escherichia* coli (Table 2), which had the highest reported MDRO in Qatar. Hasan and colleagues studied the case of a patient infected with cytomegalovirus-associated, hemophagocytic lymphohistiocytosis that gained antimicrobial resistance. The patient colonised the multidrug-resistant MDR to *Enterococcus faecium, Escherichia coli,* and *Klebsiella pneumoniae.* As a result of the severe multidrug resistance, several infections such as respiratory, urine, and bloodstream infections, developed, causing a threat associated with the HLH patients and obtained MDR organisms. The study reported *E. coli* type ST410, which was isolated from blood. No specific strain numbers were declared for *E. coli* in this study. Using a whole-genome sequence, there were different sequence types between the vancomycin-resistant *Enterococcus* isolates from the urine and blood cultures of patients and the previously colonised patients. These authors found that the resistance mechanisms were acquired in the colonizing strains [333].

In another study, the bacterial infection and resistance patterns of pediatric oncology patients after chemotherapy treatment were analysed for MDRO in the bloodstream; 116 strains of Gram-positive and Gram-negative bacteria were isolated. *E. coli* was the third most common Gram-negative isolate with seven MDR isolates. Two patients died due to the infection of MDRO *Stenotrophomonas maltophilia* and *E. coli.* Unfortunately, no specific strain numbers and names were provided, hence they do not appear in the mortality table (Table 3) [55]. The study by Garcell et al. [78] focused on studying the etiology of surgical site infection in a community hospital in Qatar for a period of three years. Samples were collected and patients with contaminated wounds had the highest number of isolates. Most of the isolates in the surgical site were *E. coli* and *Klebsiella* spp.; 12 *E. coli* beta-lactamase (ESBL) isolates were collected from the infection in the surgical site [78]. The epidemiology of bacteremia at Hamad General Hospital isolated 167 g-positive, and 285 g-negative bacteria over a period of one year. The samples were collected from patients’ blood. Several MDRO were found; nevertheless, *E. coli* was the most common bloodstream isolate; 97 *E. coli* were isolated. The intravenous catheterisation blood stream infection was found to be the most common source of bacteraemia. In comparison with the other types of etiologic bacteria, the MDR *E. coli* ESBL caused the highest number of deaths, and accounted for 14 out of 102 mortalities [7]. Regarding the antibiotic resistance of *E. coli* in Qatar from food-producing animals, 90 *E. coli* isolates were reported from poultry farms and tested with several antibiotics. Uncooked food or unsanitary hygiene practices can accelerate the transmission of MDR to humans [75]. Having a statistical mortality on MDR microbial infection in the Arabian Peninsula is important for identifying the mortality caused by the MDRO. Qatar had the second highest number (*n* = 187) of mortalities compared to the other countries in the Arabian Peninsula, (Figure 3A). In Qatar, four studies reported information on mortalities. Several MDR organisms were recorded as the cause of a patient’s death. *Acinetobacter baumannii* was recorded as a pathogen associated with mortality in several studies (Table 3). The study by Al Samawi et al. recorded the higest number of deaths caused by *Acinetobacter baumannii*. The total number of non-survivors was 65 out of 239 patients, and 372 strains were isolated from adult patients samples. The respiratory tract was the most prominant site of *A. baumannii* infection [6]. (Table 3). Another study reported the same organism and its association with the deaths of 15 patients out of 48. A total of 48 MDR *A. baumannii* strains were isolated from the patients [4] (Table 3). Another study reported 102 deaths due to the MDR organisms, of which 34 were females. Multiple MDR pathogens caused the death of patients; nevertheless, ESBL and MSSA had the highest-recorded mortality [7] (Table 3). Although several MDRO recorded in Qatar were found in other parts of the Arabian Peninsula, *Campylobacter* spp., *Nacardia crassostreae, Chryseobacteriu indologenes, Acinetobacter* spp., *Bacteroid* spp., coagulase negative staphylococci, and *Enterococcus* spp. were considered unique to Qatar.

### 2.6. United Arab Emirates

The search terms related to multidrug resistance resulted in 71 studies from PubMed and 38 studies from Scopus. After removing duplicates, peer reviewed articles, and non-relevant countries of interest, a total of 70 articles were screened. Fifty-one studies were excluded from further analyses and 19 articles were screened for eligibility as full-text articles by screening the abstract. Four full-text articles were eliminated because patients were not from the country of interest. A total of fifteen full-text articles were included in this study (Supplementary Table S1). 

Twelve different species of multidrug-resistant organisms were reported in the UAE (Figure 2). *Escherichia coli* was the most abundant MDRO in the UAE and was reported in six studies (Table 2). Although several MDRO were recorded in this country, the following organisms were reported as the lowest among the collected studies: *Pseudomonas aeruginosa*, *Enterobacter cloacae, Candida auris, Citrobacter freundii, Staphylococcus epidermidis,* and *Salmonella typhi* (Table 2). The total strain numbers of MDROs reported in the UAE were: 1116 *Mycobacterium tuberculosis*, 73 *Enterobacteriaceae*, 61 *Escherichia coli*, 55 *Klebsiella pneumoniae*, 31 *Pseudomonas aeruginosa*, 16 *Acinetobacter baumannii*, 6 methicillin-resistant *Staphylococcus aureus*, and 1 *Stenotrophomonas maltophilia* (Supplementary Table S2). 

*E. coli* was the most abundant MDRO in UAE, and the highest reported by several articles. The study by Sonnevend et al. focused on the presence of the *mcr-1* gene in colistin resistance to *E. coli* from several countries in the Arabian Peninsula. Four *E. coli* strains were isolated in this study; only one strain was from UAE, named ABC149 with the sequence type ST131. This sample was isolated from patients’ blood samples. In the Arabian Peninsula, several multi-resistance isolates contained the *mcr-1* gene, which could increase mcr-1-carrying strains in these regions [1]. The impacts of ceftolozane–tazobactam and ceftazidime-avibactam against multidrug-resistant isolates such as *Escherichia coli, Klebsiella pneumoniae,* and *Pseudomonas aeruginosa* were studied, and included 60 CRE *E. coli* from different patient samples such as sputum, blood, urine, tissue, and body fluid samples. The study suggested that ceftolozane–tazobactam and ceftazidime–avibactam could treat most of the infections caused by MDROs [45]. Another study focused on the pattern of uropathogenic resistance to antibiotic prophylaxis in patients infected in the urinary tract. *E. coli* was the most grown organism with prophylaxis. The number of *E. coli* isolates was 26, and the majority of them were isolated from urine [97]. The activity of hymenochirin-1B against multidrug-resistant bacteria and immunomodulatory properties were studied [182] such as the *E. coli* strains, ABC 54 and ABC 85, collected from urine and tracheal aspirate. The study revealed a potential drug development, combined with hymenochirin-1B, against several Gram-positive and Gram-negative bacteria, with low cytotoxicity in humans. The characteristics of *E. coli* in several counties in the peninsula region revealed the presence of the New Delhi Metallo-beta-lactamase (NDM)-7, four stains showed a multi-drug resistance of NDM-7 *E. coli*, including two strains (ABC133 and ABC218) from the UAE region of patient’s sputum and wound secretion samples. The blaNDM7 was identified on IncX3 type plasmids and an association was found between IncX3 type plasmids and the transmission of the rare NDM-7 variant in the Middle East [101]. Mortality data were not declared in the studies; which is one of the limitations of such data. Further studies are needed to find the mortality data, which can be correlated with the MDRO. All twelve of the different species of multidrug-resistant organisms reported in the UAE were also reported in other countries in the Arabian Peninsula (Table 1). These organisms were able to survive in other parts of the Arabian Peninsula with hot dry summers and mild wet winters. 

### 2.7. Jordan

The screening of multidrug-resistant micro-organisms from Jordan through the databases PubMed, Scopus, and Google Scholar revealed 136, 92 and 22,400 published articles related to multidrug resistance, respectively. After filtering out review papers and limiting the search results timeline to 2010–2021, thirty-seven articles were screened and six papers were excluded, since their samples were reported from ATCC standard cultures rather than human samples, resulting in a total of 31 full-text articles for final consideration. Jordan reported 11 distinct, multidrug-resistant bacterial species; *Escherichia coli* was the dominant species with 886 isolates in total, and *Acinetobacter* had the lowest number of strains reported, with 19 [33]. Out of all countries in the Arabian Peninsula, Jordan is the only country that reported *Propionibacterium acnes* as a unique multidrug-resistant bacterium. Research articles reported a variety of strains that were isolated from humans, hospital environments, or other sources where meat products and green vegetables could be present [334]. Some articles reported a large number of bacterial strains isolated from the aforementioned sources. For instance: 269 *E. coli* strains were isolated from broiler chickens [85], another study reported 287 *Salmonella enterica* isolates from beef cattle [100], while 544 *Streptococcus pneumoniae* isolates were obtained from humans [268].

*E. coli* were the dominant MDR bacteria in 11 research studies. One study isolated 142 *E. coli* ST131 strains from infants’ faecal samples, of which the majority were ESBL producers. The antimicrobial susceptibility of these strains was tested using the disc diffusion technique [64]. Similarly, Haddadin et al. found 19 and 25 MDR *E. coli* strains from 70 strains isolated from green vegetables and 68 isolated from infants’ faeces, respectively, after testing them for antibiotic susceptibility. In addition, they found a genetic similarity between the strains isolated from plants and infants, implying the possibility of the circulation of these pathogens between both sources [80]. O1, O2, O25, and O78 avian *E. coli* serotypes were characterised in a study after their isolation from broiler chickens, in order to study their antimicrobial resistance and the associated risk factors [85]. Another study investigated the cause of urinary tract infections among patients in Jordan. They isolated 262 *E. coli* strains from urine samples, of which 150 were MDR with a high association with the ST131 clone [96]. In addition, for the purpose of studying the colonisation of *E. coli* in the intestines, 288 stool samples were collected from infants. From these isolates, 52 were MDR and ESBL producers [104].

It was crucial to include mortality data in this review to observe the number of deaths caused solely by MDR micro-organisms and the root cause of their ability to cause a high mortality in the Arabian Peninsula (Table 4). Regarding mortality data collected from Jordan, several bacterial species contributed to these numbers. Specifically, Almomani et al. reported the deaths of 50 patients (24 males and 26 females) out of 119 patients suffering from pneumoniae caused by using ventilators infected with *Acinetobacter baumannii* in ICUs. This article reported the highest number of mortalities in Jordan [34] (Table 4). MDR *Acinetobacter* sepsis cases in neonates were also studied to determine the effect of Colistin; this organism caused two deaths out of twenty-one neonates, averaging 0.6 years of age [33]. Furthermore, there were 457 wound infection cases at a Jordanian hospital that included 395 males and 62 females with a median age of 27 years. These infections were caused by several MDR micro-organisms, including *Staphylococcus aureus*, *E. coli*, *Klebsiella pneumoniae*, *Pseudomonas*, *Enterobacter*, *Acinetobacter*, and *Proteus* and 37 deaths were reported [32]. 

### 2.8. Iraq

The number of final research articles from Iraq (*n* = 75) was generally higher than those reported from other countries in the Arabian Peninsula, with the exception of Saudi Arabia (*n* = 198), in PubMed and Scopus, and Google Scholar (Supplementary Table S1) for the keywords “multidrug-resistant”, and “multidrug resistance”. After excluding irrelevant articles, review articles, and limiting the timeline to 2010–2021, 75 research articles were eventually obtained for further analysis. *A. baumannii* was the dominant species in Iraq, followed by *E. coli*, with 10 research reports among the 28 different bacterial species reported, including four *Staphylococcus* species and three *Klebsiella* species. However, the largest number of strains were from *Providencia* spp.; 1213 isolates were reported from only two studies [49,261]. Several varieties of bacterial strains were reported from Iraq; these included 1063 *S. aureus* isolated from humans [15], 142 *E. coli* isolated from chickens [37], and 1209 *Providencia* spp. from various sources such as humans, soil, chickens, and wastewater [49]. Iraq is the only country in the Arabian Peninsula that reported the multidrug-resistant *Vibrio cholerae*, making it unique to this region [316].

A total of 12 research articles reported *A. baumannii* MDR strains. Metallo ג-lactamases producing *A. baumannii* caused a nosocomial infection in clinical samples from patients’ sputum, blood, urine, and burn wound swabs, in addition to samples from the environment; 124 strains were acquired from Baghdad hospitals to study their multidrug resistance [189]. A study conducted by Muslim et al. to determine the lectin-producing ability among 51 *A. baumannii* isolates from a hospital environment and patient samples of the sputum, blood, wounds and urine, revealed 17 lectin producers [183]. In addition, a study isolated 96 *A. baumannii* from clinical samples of wound and burn infections tested against 16 antimicrobial drugs using the disc diffusion technique, and 84 were found to be MDR [174]. Another article reported carbapenem resistance in 44 *A. baumannii* isolates from 182 patients who suffered wound infections, samples included swabs from joints, bones, and connective tissues [162]. In addition, the virulence factors of 30 *A. baumannii* strains from patients with blood infections were determined, implying their ability to cause persistent infections due to harboring *plc*N and *las*B genes [177]. Even though, in general, there were many reports from Iraq, only one article reported mortality data compared to the other countries in the Arabian Peninsula. In 2012, Babakir-Mina et al. reported that, out of 654 burn patients, 98 died due to infections caused by *S. aureus*, while 556 survived (Table 3). These burn patients were mainly females (381) with a median age of 18 years. The nosocomial infection-causing agent was the methicillin-resistant *S. aureus* (MRSA), with a total of 1063 isolates from the burn swabs [15]. This study alone does not reflect the total mortality caused by MDR micro-organisms in Iraq. More research must be conducted in order to determine the dominant micro-organisms and the root cause of mortality from such organisms.

### 2.9. Yemen

Yemen had much fewer search results in PubMed, Scopus, and Google Scholar when the keywords, “multidrug-resistant”, and “multidrug resistance” were used. For instance, there were 19 results from PubMed, 38 from Scopus, and 2269 from Google Scholar; articles from Google Scholar were not included. Many search results showed articles reporting parasitic infections in some regions in Yemen and their multidrug resistances, but these were also not included, in addition to irrelevant and older articles published before 2010. Eventually, only six articles were included from the databases PubMed and Scopus for a final analysis. Yemen reported only four distinct bacterial species dominated by *Mycobacterium tuberculosis*, with 240 strains from four research articles. The remaining three articles reported *A. baumannii*, *Pseudomonas aeruginosa*, and *S. aureus*. All the bacterial isolates in these articles were from human sources, including 120 strains of *M. tuberculosis* [115], 60 of *S. aureus* [270], and 65 of *P. aeruginosa* [237]. As mentioned, the number of reports from Yemen were generally fewer compared to the other countries studied; three research articles mentioned the isolation of *M. tuberculosis* from patients residing in tuberculosis (TB) centers in Yemen. For instance, MDR *M. tuberculosis* were isolated from 115 patients’ sputum samples to evaluate the associated risk factors [17]. In a similar study was conducted by the same author, in which MDR *M. tuberculosis* involving 135 patients was explored to evaluate health-related risk factors [16]. Furthermore, sputum samples were collected from 170 patients in the National TB Institute of Sana’a and 118 *M. tuberculosis* isolates were identified [115]. Two articles, both written by Jaber et al., reported mortality data in Yemen [16,17]. The first study reported 14 deaths out of 115 tuberculosis patients (65 males and 50 females), with a median age of 45 years, while being treated in TB centers. All of the isolated *M. tuberculosis* were resistant to at least two drugs. The other study also reported the isolation of *M. tuberculosis* from sputum samples. This micro-organism caused 14 deaths out of 80 patients (48 males and 32 females) who were also in TB centers [16]. Mortality data were limited to patients treated in TB centers, extensively focusing on MDR *M. tuberculosis* per se. More extensive mortality studies must be conducted to investigate the prominent MDR micro-organisms causing substantial death cases in the Arabian Peninsula in general, particularly in Yemen.

## 3. Methods Used to Detect Multidrug-Resistant Organisms

Various detection methods are used to identify MDR organisms: the disc diffusion method [300,330], Kirby and Bauer method [335], microdilution process [161], PCR with specific primers [336], real-time PCR tests with temperature melting analysis [49], MDR-TB detection [125], E-test for antimicrobial resistance [4,148], automated identification and susceptibility system [330], Growth Indicator Tube system [337] automated Phoenix method [221], matrix-assisted laser desorption/ionisation time-of-flight mass spectrometry (MALDI-TOF MS) [4], GeneXpert assay [338], AcrAB–TolC efflux pump system [36], multiplex PCR for confirming isolates [300,317], multilocus sequence typing [300], sequences of *18S rRNA* gene and ITS region for MDR fungi [24], *16S rRNA* gene sequencing [317], toxin genes multiplex PCR [317], multiplex real-time quantitative PCR [339], DNA Microarray Detection [84] and whole-genome sequencing [21].

The resistance of antibiotics was established for the *A. baumannii* isolates 506, 510 and 936 isolated in 2006, 2009 and 2012, respectively. In 2012, 12 unique XDRAB strains were extracted from critical patients with ventilator-associated pneumonia (VAP) using the microdilution process [161]. Minimum Inhibitory Concentration (MICs) were indicated by E-test. A CHROMagar Acinetobacter medium was used to diagnose susceptible and multi-drug-resistant *A. baumannii* (MDRAB) strain. The updated Hodge test (MHT) and multiplex PCR were used to examine carbapenemase-resistant *A. baumannii*. The E-test process was used to carry out a synergy test. A recent 3-year evaluation study determined a reduction in the antibiotic resistance in Gram-negative bacteria by the Saudi National Action Plan using commercially available identification cards (VITEK 2 ID-GNB cards) and MDR detection (AST-No. 12 cards) [340]. They found that the rates of antibiotic resistance, extended-spectrum beta-lactamase, and multidrug resistance were reduced over time, indicating that the Saudi National Action Plan was effective. Yassin et al., [299] reported the advent of *Candida* species resistance and suggested an antifungal sensitivity procedure performed prior to deciding on a treatment regimen. Updated techniques such as sequencing the *18S rRNA* gene and ITS region in MDR fungi, *16S rRNA* gene sequencing in bacteria, and toxin genes multiplex PCR were also adopted by laboratories in Saudi [24,317]. Whole-genome-sequencing-based MDR identifications were also reported [21]. Improved tools are needed for the diagnosis of MDRO [341,342].

*Candida auris* is an emerging fungal pathogen with multidrug resistance, causing nosocomial infections and invasive deaths [336]. A trustworthy diagnostic approach, such as using the *18S rRNA* gene and ITS region against MDR fungi, is urgently needed [24]. *C. auris* is often mistaken for numerous other yeast species by phenotypic identification platforms [336]. In this work, PCR and real-time PCR tests were used for identifying *C. auris* and related species: *Candida duobushaemulonii, Candida haemulonii* and *Candida lusitaniae*. Targeted rDNA nucleotide region sequences, *C. auris*-specific, primers-based PCR or real-time PCR experiments followed by electrophoresis or temperature melting analysis, respectively, were performed using a panel of 140 clinical fungus isolates [336]. The findings of the tests completely corroborated the findings of the DNA sequencing. These genetic techniques address the current phenotypical shortcomings in the identification of *C. auris* and its associated species. The present MDR-TB outbreak is the result of decades of neglecting significant infectious diseases, a lack of national prevention program services, slow case identification, and inadequate treatment in high-burden countries [113]. The clinical results were vastly enhanced by optimizing care regimes along with the quick detection of DST in first- and second-line medications. The prognosis of MDR-TB was further strengthened by the recent advancements in MDR-TB detection and an intensive empirical management of patients with multiple medications during the initial period of treatment. However, laboratory funding, which is essential for the effective diagnosis of patients with MDR-TB, was largely insufficient. The rapid and cost-effective cultivation of DST systems of *M. tuberculosis* strains must be strengthened.

A pilot study from Hamad Medical Corporation (HMC), supported carbapenem resistance in randomly selected clinical isolates of multidrug-resistant *Acinetobacter baumannii* [4]. The research results were used to analyse carbapenemasal in all of the isolated MDR *A. baumannii* through molecular techniques. Matrix-assisted laser desorption/ionisation time-of-flight mass spectrometry verified the identification of MDR isolates, which was verified and validated by E-test antibiotic tolerance. Real-time PCR was used to explore the molecular nature of carbapenemases (blaOXA-23, blaOXA-24, blaOXA-58, blaNDM). The phylogenetic study on the partial sequence of CsuE and blaOXA-51 genes confirmed the epidemiological relationship between the isolates. The 48 isolates were resistant to most antibiotics, including meropenem, imipenems, ciprofloxacin, levofloxacin, amicacin, gentamicin, and most β-lactam; yet they were susceptible to colistin. The MDR *A. baumannii* production of OXA-23 carbapenemase in Qatar was described in detail by Rolain [4]. The effect of a 1 and 2 h exposure to low-frequency magnetic fields (0.3 and 0.42 mT) on *Pseudomonas aeruginosa* resulted in a reduced resistance and increased drug susceptibility [225]. This increase was often linked to the temperature of the environment and the duration of the exposure. Antibiotic resistance in Iraq was conferred on *Escherichia coli* by the AcrAB–TolC efflux pump system [36], which contributed to poor patient results. 

Another study examined 177 chicken samples using the ISO 10272-2006 procedure to isolate *Campylobacter* spp. A multiplex PCR was used for the confirmation and labelling of the isolates [300]. The disc diffusion method was used to assess the isolates’ antimicrobial resistance, and multilocus sequence typing was used to genotype the isolates. There were three distinct sequence types in ten *C. coli* isolates, but seven different sequence types in ten *C. jejuni* isolates. Many of the isolates tested were resistant to ciprofloxacin but not to imipenem, and they were multidrug-resistant to five or more antimicrobials. A retrospective analysis of MDRO data from January to December 2012 used the disc diffusion method to verify antibiotic susceptibility analysis, and an automated identification and susceptibility system was used to recognise and analyse species [330]. Increasing patterns in the prevalence rates of MDRO patients and MDRO isolates were discovered during the study [330]. The study by Al Shamahy et al. examined septic-resistant organisms in the neonatal unit at Al-Thawra Hospital, Sana’a, Yemen and offered an empirical treatment approach [335]. One hundred and fifty-eight neonates, aged 0 to 28 days, were recruited in the study. Blood samples were collected from each neonate, then the samples were cultivated, and, subsequently, antimicrobial susceptibility testing was performed using the Kirby and Bauer method [335].

## 4. Antimicrobial Resistance Profiles Used for Treating Multidrug-Resistant Organisms

Combining low-frequency magnetic fields with a wide range of antibiotics helped to reduce *P. aeruginosa* tolerance [225]. The physical parameters of LF-MF, such as strength and exposure duration, influenced the reduction in bacterial resistance and provided a potentially noninvasive and fast treatment option for burn victims [225]. Peptide B1 had a broad-spectrum behaviour against the strains examined in the study by Tell et al. [343]. The concentrations used to destroy microbial cells ranged from 10 to 20 M. B1 had a lower toxicity against mammalian cells and a lower probability of causing hemolysis [343]. Domperidone is an important ArcB inhibitor, a well-known and secure over-the-counter antiemetic [36]. Domperidone reversed the antibiotic tolerance of levofloxacin and ciprofloxacin in MDR *E. coli* stains and was more effective than the recognised efflux pomp-inhibitor reserpine. It was also able to improve every impact on the sensitive strains of antibiotics. This finding suggests that a combination of antibiotics and domperidone may be utilised to handle multi-drug-resistant *E. coli* bacteria in the clinic [36]. Multidrug-resistant pneumonia in the health sector offers an important treatment challenge [12]. A Unyvero Pneumonia Application (UPA) quick PCR multiplex test helps to make prompt decisions. UPA was assessed for its successful detection in nosocomial pneumonia of the etiologic pathogen and for resistance indicators. This test also investigated the impact on acute nosocomial pneumonia. The appropriate specimens were treated by UPA according to the manufacturer’s methods, along with conventional cultivation techniques. The results indicated that a multiplex PCR-based test was capable of reliably diagnosing the etiological agents of NP and MDR, as well as resistance indicators. This could allow doctors to make early antibiotic modifications [12]. The analysis of *E. faecalis* genomes was performed by means of multilocus sequence typing. ST179 and ST16 were the most common STs in clonal complex 16 in Saudi patients (CC16). MDR isolates were approximately 96% (*n* = 149). There was nearly no coverage of the antibiotics quinupristin/dalfopristine, clindamycin, and erythromycin, but there were elevated levels of streptomycin, gentamycin and ciprophloxacin, which were observed to be under-optimal. Low vancomycin, linezolid and ampicillin resistance were found and suggested for treating *E. faecalis* infection [292]. Alqasim [57] discovered the frequency of β-lactamases produced by *E. coli.* They analysed the transportation of these isolates by suspicious antibiotic patterns at phenotypic and genotypic levels. A total of 100 *E. coli* urine isolates were collected at a tertiary health facility in Riyadh from January 2018 to March 2018. All antimicrobial isolates were tested for susceptibility. The synthesis of ESBL was separated in phenotypic and genotypic phases by double disc synergy testing and a polymerase chain response. A variety of ESBL variations were identified using DNA sequencing. Of the 100 *E. coli* isolates, 67 were associated with the phenotype of MDR. All isolates were tolerant to all the antibiotics that were used, which were all vulnerable to *imipenemia*. There were 33 phenotypic and genotypical isolates of ESBLs the in 100 isolates of *E. coli*. For all ESBL-positive *E. coli* isolates, CTX m was an ESBL (31/33 isolates; 93.94 percent). In all CTX m carriage isolates, the CTX-M-15 variant was included. The multiple ESBL carriage of 15–33 isolates (45.45%) was detected, with 11 (33.30%) ESBL isolates, compared to four isolates (12.12%) with three ESBLs. A vast range of forefront antibiotics were found by our study, which were frequently utilised for the treatment of UTI patients with a high antimicrobial toleration of *E. coli* that generated ESBL. Among the *E. coli* urinary isolates, the most common CTX m variants also had a high incidence of ESBL channels. This is a serious concern and needs to be investigated further to find better treatment options.

*M. morganii* isolates were highly resistant to many antibacterial agents and classified as MDR bacteria from Bahrain [315], all of which had ESBL. The antibiotics, meropenem and imipenem, were very effective against the *M morganii* isolates. In Jordan, 31 percent of *S. aureus* were multidrug-resistant (MDR) among the *S. aureus* isolates from wound samples [247]; however, they were vulnerable to chloramphenicol, linezolid, nitrofurantoin, rifampicin, and teicoplanin (>80%). In Qatar, a pulsed-field gel electrophoresis analysis of *P. aeruginosa* isolates from cystic fibrosis patients indicated a strong degree of resemblance, indicating a unique adaptation of these clones to cystic fibrosis-affected lungs. The non-cystic fibrosis, hospitalised cluster had a distinct clonal root with localised clustering and potential hospital-acquired *P. aeruginosa* infections [221]. *A. baumannii* isolates from Al-Thawra University hospital in Yemen were immune to almost all antibiotics examined, with a high minimum inhibitory concentrations of imipenem, amikacin, and ciprofloxacin [168]. All three *A. baumannii* strains were positive for the modified Hodge test, and no inhibition was observed on the activity of β-lactamases. The naturally occurring blaOXA-51-like gene, and the carbapenemase-encoding blaOXA-23-like gene, were observed in all three isolates and detected with 16S rRNA methylase armA gene. A search for aminuglycoside enzymes (AMEs)-producing genes identified the presence of acetyltransferase aac (6-Ib) in one *A. baumannii* isolate. Fluoroquinolone resistance-associated GyrA genes with one Ser83Leu variation were observed in all of the isolates. A multilocus sequence typing demonstrated that all the isolates were sequence type 2 [168]. A case study in pediatric patients from Saudi Arabia with combined immunodeficiency syndrome, reported treating pneumonia caused by multidrug-resistant *P. aeruginosa* using ceftolozane/tazobactam, which was recommended only for adult patients (>18 years) [241]. Peptide (AamAP1-Lysine) antibiotic (chloramphenicol, levofloxacin, rifampicin, ampicillin and erythromycin) combinations proved a strong synergistic activity, causing a 64-fold reduction in multidrug-resistant bacteria [344]. Colistin was effective and safe for treating MDR Acinetobacter neonatal sepsis in neonatal units in Amman, Jordan [33].

## 5. Mining Natural and Novel Antimicrobials against MDR

The antibacterial activities against the most common multidrug resistance micro-organisms associated with skin infections, were assayed using aqueous and ethanol extracts from five native medicinal plants, most of which showed an antibacterial and antifungal activity, with the highest activity found in the aqueous extract of *Arum discoridis* [345]. The experiments that characterised and measured the effect of honey on *P. aeruginosa* quorum sensing networks revealed that low concentrations of honey decreased the expression of exotoxin A (ETA), las and rhl glucons, as well as the corresponding virulence factors through the interruption of the quorum sensing system [346,347]. Edible plants (*Gundelie tournefortii* L. and *Pimpinella anisum* L.) and the propolis of *Apis mellifera* [348] were also considered as options for inhibiting the growth of MDR in combination with antibiotics [69]. Lavender essential oil used against carbapenemase-producing *K. pneumonia* [349], extracts of *Lawsania inermiss*, *Portulaca oleracea* used against *Candida albicans* [350], and *Trachyspermum ammi* extracts mediated the green production of metal-nanoparticles against MDR *Listeria monocytogenes* and *Serratia marcescens* [351] and were reported as suitable for the identification of novel antimicrobial agents against selective MDROs. 

Ultrashort antimicrobial peptides (novel pentapeptide, UP-5) had a strong to moderate efficacy against MDRO, with a low toxicity when used in conjunction with traditional antimicrobials [352]. Alyteserin-2a, a cationic α-helical peptide recovered from the skin secretions of *Alytes obstetricans,* showed an efficient activity against multidrug-resistant *Acinetobacter baumannii* and *Stenothrophomonas maltophilia* [172]. Cuminaldehyde, a key antibacterial ingredient of *Calligonum comosum* essential oil, was reported to have an antibacterial activity against MDROs in a study from the United Arab Emirates [353]. Actinomycetes, specifically Streptomyces, *Nocardiopsis* sp., and MK MSt033 were discovered to have an antimicrobial activity against a variety of MDROs [354]. Many peptides were tested against MDR *Acinetobacter baumannii* [355,356,357]. *Terminalia chebula, Terminalia bellirica*, and *Phyllanthus emblica* plants, traditionally used for treating microbial infections were tested against MDR bacteria and fungi [358].

Buffalo milk lactoperoxidase against *S. enterica and* L. monocytogenes [359]; colistin against MDR *Acinetobacter* [33]; lactic acid bacteria from raw camel milk [360]; peptides, including hybrid peptides and ultrashort peptides [343,344,352]; ultrashort antimicrobial peptide nanoparticles [356]; Isatin-benzoazine molecular hybrids [361]; chemical derivatives [362,363,364,365]; extracts of *Matricaria aurea* [366]; extracts of *Nepeta deflersiana* [223]; Saudi scorpion venoms [367]; artemisinin extract [368]; biocidal polymers [369]; microtubule depolymerizing agents [370]; ozonated water [250]; and marine *Streptomyces* sp [371] against MDRO were all tested in the study region. Nanomaterials, nanoparticles and nanoformulations [351,356,358,372,373], as well as silver nanomaterials derived from marine *Streptomyces* [374] were also tested against the growth of multidrug-resistant micro-organisms. Further studies are needed to understand the molecular mechanisms and future possibilities of identifying pharmaceutically active compounds with potential medicinal applications.

## 6. Conclusions and Future Perspectives

The active surveillance and constant preventive efforts to control MDR infections and associated diseases can improve healthcare facilities and the socio-economic status of patients [375]. Specifically, MDR *E. coli* is predominantly associated with urinary tract infections in pregnancy [54,61,102]. The emergence of multi drug-resistant *Candida albicans* is prevalent among pregnant and non-pregnant women with vaginitis [299]. *Listeria monocytogenes* from patients with spontaneous abortions [310] and multidrug-resistant clinical *Enterococcus faecalis* from pregnant women [292] highlight the need for more precautions and constant monitoring, with the appropriate identification of MDR and susceptibility tests prior to medication. The development of a candidate vaccine and the early diagnosis of MDR infection shall be a better option for protection against MDR infection. The most prevalent multidrug-resistant *Escherichia coli, Mycobacterium tuberculosis, Acinetobacter baumannii, Klebsiella pneumoniae, Pseudomonas aeruginosa, Staphylococcus aureus, Acinetobacter, Enterobacteriaceae, Providencia* spp., *Streptococcus pneumoniae*, methicillin-resistant *Staphylococcus aureus* (MRSA), *Klebsiella* spp., *Enterobacter, Proteus vulgaris, Candida auris* and *Enterococcus* in the Arabian Peninsula must be diagnosed accurately and rapidly using advanced molecular techniques by developing region-specific diagnostic tools. The control of infections by using specificity detecting systems is an important protection method against MDRO. One study develops a direct detecting system for *Candida auris* by using a novel multiplex real-time quantitative PCR that has a high degree of specificity in detecting different species-level of *Candida* [339]. However, in healthcare settings, more detection methods are needed to detect MDRO strains within the species in clinical samples; allowing a reduction in the evolving MDRO pathogenic species. The Arabian Peninsula is rich in religious traditions. Notably, every year, more than 10,000,000 pilgrims visit Saudi Arabia to perform the Umrah and Hajj. Multidrug-resistant *Streptococcus pneumoniae* [376], *Acinetobacter baumannii*, *Escherichia coli* [377], *Salmonella enterica* [308], and *Mycobacterium tuberculosis* [378] is observed among the pilgrims; however, it is relatively very low compared to the total number of pilgrims, which indicates the successful surveillance and management of these micro-organisms. International guidelines and recommendations can be implemented for the management and complete prevention of MDRO, such as guidelines for MDRO regarding mass gathering events. Networking MDRO research strategies are mandatory among the countries of the Arabian Peninsula to fight against the risk of MDRO infection and death. This research can enhance the sharing of information, overcome the limited data availability, and identify molecular and physiological mechanisms behind multidrug resistance. MDR co-infection with SARS-CoV-2 among COVID-19 patients shall also be considered to reduce the disease burden [379,380]. This review uncovered the huge requirement for developing novel, safe, and sustainable antimicrobials from natural and other resources to fight against MDRO. Food process parameters and the overuse of antimicrobials require active surveillance to reduce resistance modulators. The present cumulative observations will be informative for effective planning, designing preventive strategies, and prioritizing research goals to achieve MDRO-free zones.

## Figures and Tables

**Table 1:** Logical relation between sets of multidrug-resistant bacteria and fungi in countries of Arabian Peninsula.

Countries	Number of MDRO	Multidrug-Resistant Micro-Organism(s)
Bahrain, Iraq, Jordan, Kuwait, Oman, Qatar, Saudi Arabia, United Arab Emirates, Yemen	1	*Acinetobacter baumannii*
Bahrain, Iraq, Jordan, Kuwait, Oman, Qatar, Saudi Arabia, United Arab Emirates	1	*Escherichia coli*
Iraq, Jordan, Kuwait, Oman, Qatar, Saudi Arabia, United Arab Emirates, Yemen,	3	*Mycobacterium tuberculosis, Staphylococcus aureus, Pseudomonas aeruginosa*
Iraq, Kuwait, Oman, Qatar, Saudi Arabia, United Arab Emirates	1	*Klebsiella pneumoniae*
Kuwait, Oman, Qatar, Saudi Arabia, United Arab Emirates	2	*Enterobacter cloacae, Stenotrophomonas maltophilia*
Iraq, Kuwait, Oman, Qatar, Saudi Arabia	1	*Enterococcus faecium*
Iraq, Jordan, Kuwait, Qatar, Saudi Arabia	1	*Streptococcus pneumoniae*
Iraq, Jordan, Kuwait, Saudi Arabia	1	*Salmonella enterica*
Iraq, Qatar, Saudi Arabia, United Arab Emirates	1	*Salmonella typhi*
Kuwait, Saudi Arabia, United Arab Emirates	1	*Candida auris*
Iraq, Kuwait, Saudi Arabia, Oman	3	*Serratia marcescens, Proteus mirabilis, Klebsiella oxytoca*
Iraq, Saudi Arabia, United Arab Emirates, Oman	1	*Citrobacter freundii*
Iraq, Jordan, United Arab Emirates, Qatar	1	*Staphylococcus epidermis*
Kuwait, Saudi Arabia	1	*Candida glabrata*
Oman, Saudi Arabia	1	*Pantoea agglomerans*
Qatar, Saudi Arabia	1	*Vibrio vulnificus*
Iraq, Saudi Arabia	6	*Klebsiella aerogenes, Morganella morganii, Citrobacter koseri, Pantoea* spp., *Providencia* spp., *Staphylococcus saprophyticus*
Iraq, Saudi Arabia, Qatar		*Staphylococcus haemolyticus*
Kuwait,	1	*Enterococcus gallinarum*
Iraq, Jordan	2	*Listeria monocytogenes, Campylobacter jejuni*
Saudi Arabia, Qatar	4	*Staphylococcus hominis, Klebsiella* spp., *Salmonella* spp., *Streptococcus* spp.
Iraq, Qatar	1	*Enterobacter* spp.
Saudi Arabia	53	*Staphylococcus capitis, Staphylococcus caprae, Acinetobacter calcoaceticus baumannii, Pseudomonas, Bacillus subtilis, Leuconostoc mesenteroides, Candida albicans, Staphylococcus epidermidis, Proteus vulgaris, Proteus sp., Bacillus cereus, Lactobacillus plantarum, Bipolaris oryzae, Methicillin-resistant Staphylococcus aureus, Bacillus tequilensis, Staphylococcus, Enterococcus, Staphylococcus saccharolyticus, Leclercia adecarboxylata, Shigella flexnari, Staphylococcus* spp., *Pantoea eucrina, Enterobacteriaceae, Citrobacter youngae, Staphylococcus petrasii, Lactococcus garvieae, Escherichia fergusonii, Caldimonas manganoxidans, Chryseobacterium gleum, Citrobacter sp., Providencia stuartii, Vancomycin-resistant Enterococcus, Mycobacterium leprae, Clostridium* spp., *Stenotrophomonus maltophilia, Enterococcus faecalis, Acinetobacter, Non-typhoidal Salmonella, Enterobacter aerogenes, Acinetobacter lwoffii, A. baumannii complex, A. baumannii/haemolyticus, Acinetobacter haemolyticus, Pseudomonas luteola, Serratia fonticola, Penicillin-resistant Streptococcus pyogenes, Bacillus* spp., *Serratia* spp., *Candida* spp.
Kuwait	5	*Legionella pneumophila, Aeromonas hydrophila, Moraxella catarrhalis, Prevotella* spp., *Haemophilus influenzae*
Oman	5	*Pantoea dispersa, Burkholderia cepacia, Enterobacter asburiae, Enterobacter hormaechei Enterobacter ludwigii*
Qatar	7	*Campylobacter* spp., *Nocardia crassostreae, Chryseobacterium indologenes, Acinetobacter* spp., *Bacteroid* spp., *Coagulase negative Staphylococci, Enterococcus* spp.
Jordan	1	*Propionibacterium acnes*
Iraq	1	*Vibrio cholerae*

**Table 2:** Total number of studies that reported the MDR microbe in countries of the Arabian Peninsula.

S. No	MDR Microbe	Saudi Arabia	Bahrain	Kuwait	Oman	Qatar	United Arab Emirates	Jordan	Iraq	Yemen	Total Number of Reports *
1	*Acinetobacter baumannii*	59	1	8	2	2	5	6	12	1	96 ^#^
2	*Escherichia coli*	51	2	4	5	6	6	11	10		94
3	*Mycobacterium tuberculosis*	31		11	2	1	2	1	6	4	58
4	*Klebsiella pneumoniae*	36		3	4	4	4		7		58
5	*Pseudomonas aeruginosa*	36		4	1	4	1	1	9	1	57
6	*Staphylococcus aureus*	16		4	1	1	2	5	5	1	35
7	*Acinetobacter*	14		2							16
8	*Streptococcus pneumoniae*	7		1		1		2	1		12
9	*Stenotrophomonas maltophilia*	7			1	1	2				11
10	*Enterobacter cloacae*	6		1	2	1	1				11
11	*Candida auris*	4		6			1				11
12	*Enterobacteriaceae*	6		5							11
13	*MRSA*	11									11
14	*Stenotrophomonus maltophilia*	6		1							10
15	*Salmonella enterica*	2		1				2	4		9
16	*Proteus mirabilis*	8		1							9
17	*Serratia marcescens*	3		3	1				2		8
18	*Enterococcus faecalis*	8									8
19	*Citrobacter freundii*	4			1		1		1		7
20	*Enterococcus faecium*	6									6
21	*Klebsiella oxytoca*	2		2					2		6
22	*Listeria monocytogenes*							2	3		5
23	*Morganella morganii*	4							1		5
24	*Staphylococcus epidermidis*	3				1	1		1		5
25	*Salmonella* spp.	4				1			1		5
26	*Klebsiella* spp.	4		1		1					5
27	*Candida* spp.	4		1							5
28	*Streptococcus* spp.	2		3		1					5
29	*Enterobacter*	4		1							5
30	*Enterococcus*	3		2							5
31	*Staphylococcus* spp.	5									5
32	*Salmonella typhi*	2				1	1		1		5
33	*Enterobacter aerogenes*	5									5
34	*Staphylococcus hominis*	4				1					4
35	*Candida albicans*	4									4
36	*Non-typhoidal Salmonella*	2		2							4
37	*Citrobacter koseri*	2							1		4
38	*Klebsiella aerogenes*	4									4
39	*Enterococcus faecalis/faecium*			1	1				1		3
40	*Providencia* spp.	1							2		3
41	*Pantoea* spp.	1							2		3
42	*Providencia stuartii*	3									3
43	*Acinetobacter lwoffii*	3									3
44	*Serratia* spp.	3									3
45	*Campylobacter jejuni*							1	1		2
46	*Staphylococcus (MRSA)*			2							2
47	*Proteus sp.*	1		1							2
48	*Candida glabrata*	1		1							2
49	*Pantoea agglomerans*	1			1						2
50	*Bacillus subtilis*	2									2
51	*Staphylococcus capitis*	2									2
52	*Staphylococcus saprophyticus*	2									2

* MDR organisms reported more than once in the countries of Arabian Peninsula are listed: # includes *Acinetobacter calcoaceticus baumannii =* 2, *A. baumannii* complex = 1 and *A. baumannii/haemolyticus* = 1. MRSA: methicillin-resistant *Staphylococcus aureus*.

**Table 3:** Reports of mortality caused by MDR microbial infection from Qatar, Kuwait, Iraq and Yemen.

Country	Qatar	Qatar	Qatar	Qatar	Kuwait	Kuwait	Kuwait	Kuwait	Kuwait	Kuwait	Kuwait	Iraq	Yemen	Yemen
MDR/Reference	[4]	[5]	[6]	[7]	[8]	[9]	[10]	[11]	[12]	[13]	[14]	[15]	[16]	[17]
Total cases	48	10	239	452	61	71	14	15	49	11	1430	654	115	80
Median age years	48	43	49.1	45.98	58	59.5	63.9	56.6	55.6	49.8	57.5	18	45	45
Gender F		1	57	159	19	24	5	6	15	6	730	381	50	32
Gender M		9	182	293	42	47	9	9	34	5	700	273	65	48
No. of Discharged patients		5	174	350		34		6	46	9	1415	556	101	66
Total number of non-survivors	15	5	65	102	4	37	9	8	3	2	15	98	14	14
Number of non-survivors F				35	2		3	3		1				
Number of non-survivors M		5		67	2		6	5		1				
Multiple MDR pathogens				102			9		3	2	15			
*Acinetobacter baumannii*	15		65											
*Pseudomonas aeruginosa*														
*Staphylococcus aureus*												98		
*Mycobacterium tuberculosis*													14	14
*Escherichia coli*														
*Klebsiella pneumoniae*		5												
*Staphylococcus epidermidis*														
*Enterobacteria*														
*Candida auris*						37		8						
*Acinetobacter*														
*Enterobacteriaceae*														
*Proteus sp.*														
*Salmonella typhi*					1									
*Salmonella enterica*					1									

**Table 4:** Reports of mortality caused by MDR microbial infection from Saudi Arabia and Jordan.

Country	Saudi Arabia	Saudi Arabia	Saudi Arabia	Saudi Arabia	Saudi Arabia	Saudi Arabia	Saudi Arabia	Saudi Arabia	Saudi Arabia	Saudi Arabia	Saudi Arabia	Saudi Arabia	Saudi Arabia	Saudi Arabia	Jordan	Jordan	Jordan	Jordan
MDR/Reference	[18]	[19]	[20]	[21]	[22]	[23]	[24]	[25]	[26]	[27]	[28]	[29]	[30]	[31]	[32]	[33]	[34]	[34]
Total cases	49	83	60	71	2	90	5	7	19	713	86	3	38	146	457	21	119	56
Median age years	newborn	50	50.4	37.5	73.5	59.9	63.6	63	57	37	56	43	60.5	47.3	27	0.6	55.5	53.4
Gender F	11			23	0	32		1	10	294	31	3	14	42	62		64	22
Gender M	18		37	48	2	58	5	6	9	419	55		24	104	395		55	34
No. of Discharged patients	26	30				34					47	2			198	19	69	30
Total number of non-survivors	3	31	54	7	2	54	3	3	4	110	39	1	13	41	37	2	50	26
Number of non-survivors F	0									34	14	1					26	12
Number of non-survivors M	3									76	25						24	14
Multiple MDR pathogens		31									39			41	37			
*Acinetobacter baumannii*			54														50	26
*Pseudomonas aeruginosa*						54			4									
*Staphylococcus aureus*																		
*Mycobacterium tuberculosis*				7						110								
*Escherichia coli*																		
*K. pneumoniae*																		
*Staphylococcus epidermidis*	3																	
*Enterobacteria*																		
*Candida auris*					2		3	3				1						
*Acinetobacter*																2		
*Enterobacteriaceae*													13					
*Proteus sp.*																		
*Salmonella typhi*																		
*Salmonella Enteritidis*																		

**Table 5:** Number of multi drug-resistant strains reported in the Arabian Peninsula. Details of the number of strains are presented in the Supplementary Table S2.

MDRO	Total Strain	Reference
*Escherichia coli*	13254	[1,3,7,10,11,14,31,35,36,37,38,39,40,41,42,43,44,45,46,47,48,49,50,51,52,53,54,55,56,57,58,59,60,61,62,63,64,65,66,67,68,69,70,71,72,73,74,75,76,77,78,79,80,81,82,83,84,85,86,87,88,89,90,91,92,93,94,95,96,97,98,99,100,101,102,103,104,105,106,107,108,109,110,111]
*Mycobacterium tuberculosis*	13087	[16,17,21,27,38,50,51,56,99,112,113,114,115,116,117,118,119,120,121,122,123,124,125,126,127,128,129,130,131,132,133,134,135,136,137,138,139,140,141]
*Acinetobacter baumannii*	5600	[2,6,12,28,40,42,46,47,48,51,55,56,58,63,73,88,93,94,103,105,108,111,142,143,144,145,146,147,148,149,150,151,152,153,154,155,156,157,158,159,160,161,162,163,164,165,166,167,168,169,170,171,172,173,174,175,176,177,178,179,180,181,182,183,184,185,186,187,188,189,190,191,192,193,194,195,196,197,198,199]
*Klebsiella pneumoniae*	5480	[3,5,10,11,12,14,28,30,38,39,40,41,42,45,46,47,48,51,52,55,56,58,62,63,67,81,82,83,84,88,91,92,93,105,106,111,127,143,149,155,160,181,196,200,201,202,203,204,205,206,207,208,209,210,211,212,213,214,215,216,217,218,219,220]
*Pseudomonas aeruginosa*	3445	[11,12,14,23,26,28,31,38,41,42,45,46,47,48,50,51,52,55,56,58,62,63,81,82,83,88,90,91,93,94,109,111,143,149,160,162,180,181,184,190,194,195,196,221,222,223,224,225,226,227,228,229,230,231,232,233,234,235,236,237,238,239,240,241]
*Staphylococcus aureus*	3133	[7,11,12,15,42,46,56,58,62,65,73,90,108,143,149,242,243,244,245,246,247,248,249,250,251,252,253]
*Acinetobacter*	2033	[6,14,31,33,38,41,65,66,73,187,194,254,255,256]
*Enterobacteriaceae*	1411	[3,13,19,20,73,101,176,190,232,257,258,259]
*Providencia* spp.	1216	[19,232,260,261]
*Streptococcus pneumoniae*	1077	[56,262,263,264,265,266,267,268,269]
*Methicillin-resistant Staphylococcus aureus (MRSA)*	1000	[9,11,19,31,46,47,56,93,142,173,176,181,182,190,270,271,272,273,274,275,276,277,278]
*Klebsiella* spp.	927	[31,65,66,73,94,108,111,279]
*Enterobacter*	815	[11,31,41,48,50,65,66,94,280]
*Proteus vulgaris*	722	[40,42,232]
*Candida auris*	580	[9,11,22,24,25,29,281,282,283]
*Enterococcus*	553	[65,66,81,105,108,284,285]
*Pseudomonas* spp.	481	[46,65,66,73,83,108]
*Klebsiella oxytoca*	434	[12,40,42,46,50,105,143,205,232]
*Salmonella enterica*	413	[100,286,287,288]
*Serratia marcescens*	289	[12,40,41,42,46,47,51,88,111,143,149,289]
*Stenotrophomonus maltophilia*	257	[31,41,47,65,66,290]
*Enterococcus faecalis*	256	[42,47,58,93,143,181,291,292]
*Vancomycin resistant Enterococcus (VRE)*	255	[19,73,190,293]
*Non-typhoidal Salmonella*	248	[8,203,294]
*Vibrio vulnificus*	234	[295,296]
*Salmonella typhi*	231	[8,297,298]
*Proteus sp.*	220	[12,38,41,48,51,65,108,143]
*Candida* spp.	180	[65,66,299]
*Stenotrophomonas maltophilia*	149	[40,46,48,111,172,190,256]
*Proteus mirabilis*	137	[14,42,46,58,62,81,82,91,93,105,111,143,181,232]
*Campylobacter jejuni*	125	[250,300,301,302]
*Arcobacter butzleri*	100	[303]
*Pantoea* spp.	99	[143,304,305]
*salmonella* spp.	85	[46,98,105,232,306,307,308,309]
*Listeria monocytogenes*	83	[40,100,309,310,311]
*Staphylococcus* spp.	78	[46,74,108,291]
*Bacillus* spp.	77	[312]
*Citrobacter freundii*	66	[42,81,91,105,143,232,313]
*Serratia* spp.	66	[65,66,232]
*Staphylococcus epidermidis*	59	[18,46,55,314]
*Enterobacter cloacae*	54	[10,42,46,58,81,83,93,105,106,110,111,143]
*Morganella morganii*	54	[42,46,58,83,111,143,315]
*Citrobacter* spp.	54	[11,38,41,83,232]
*Enterobacter aerogenes*	40	[42,46,83,91,111,143]
*A. baumannii/haemolyticus*	32	[144]
*Streptococcus* spp.	23	[58,108]
*Vibrio vulnificus*	23	[296]
*Staphylococcus saprophyticus*	20	[42,51,88,143]
*Arcobacter cryaerophilus*	20	[303]
*Vibrio cholerae*	20	[316]
*A. baumannii complex*	19	[144]
*Clostridioides difficile*	18	[317]
*Enterococcus faecium*	17	[42,46,143,149,318]
*Salmonella Enteritidies*	15	[8]
*Prevotella* spp.	14	[319]
*Nocardia crassostreae*	13	[267,320]
*Candida albicans*	11	[190]
*Staphylococcus hominis*	11	[46,55]
*Acinetobacter lwoffii*	11	[42,143,144]
*Providencia stuartii*	10	[46,48,58]
*Acinetobacter calcoaceticus baumannii*	10	[321]
*Citrobacter koseri*	9	[46,58,111,155]
*Pseudomonas luteola*	9	[42]
*Pantoea agglomerans*	9	[42]
*Staphylococcus capitis*	4	[46]
*Acinetobacter haemolyticus*	4	[144]
*Serratia fonticola*	4	[42]
*Candida glabrata*	2	[322]
*methicillin-resistant Staphylococcus epidermidis (MRSE)*	1	[47]
*penicillin-resistant Streptococcus pyogenes*	1	[47]
*Leclercia adecarboxylata*	1	[323]
*Mycobacterium leprae*	1	[324]
*Chryseobacterium gleum*	1	[325]
*Enterococcus gallinarum*	1	[149]
*Klebsiella oxytoca*	1	[10]

## Data Availability

All data are available upon reasonable request from the corresponding author.

## Supplementary Materials

The following are available online at https://www.mdpi.com/article/10.3390/biology10111144/s1, Table S1: Country wise data for the number of articles from various database, Supplementary Table S2: Number of multi drug resistant strains reported from countries of Arabian Peninsula, Supplementary Table S3: Source of isolates and Number of MDR microbial strains reported from Arabian Peninsula.

## References

[1] Sonnevend Á., Ghazawi A., Alqahtani M., Shibl A., Jamal W., Hashmey R., Pal T. (2016). Plasmid-Mediated Colistin Resistance in Escherichia coli from the Arabian Peninsula. Int. J. Infect. Dis..

[2] Zowawi H.M., Sartor A.L., Sidjabat H.E., Balkhy H.H., Walsh T.R., Al Johani S.M., AlJindan R.Y., Alfaresi M., Ibrahim E., Al-Jardani A. (2015). Molecular Epidemiology of Carbapenem-Resistant Acinetobacter Baumannii Isolates in the Gulf Cooperation Council States: Dominance of OXA-23-Type Producers. J. Clin. Microbiol..

[3] Sonnevend Á., Ghazawi A.A., Hashmey R., Jamal W., Rotimi V.O., Shibl A.M., Al-Jardani A., Al-Abri S.S., Tariq W.U.Z., Weber S. (2015). Characterization of Carbapenem-Resistant Enterobacteriaceae with High Rate of Autochthonous Transmission in the Arabian Peninsula. PLoS ONE.

[4] Rolain J.-M., Loucif L., Al-Maslamani M., Elmagboul E., Al-Ansari N., Taj-Aldeen S., Shaukat A., Ahmedullah H., Hamed M. (2016). Emergence of Multidrug-Resistant Acinetobacter Baumannii Producing OXA-23 Carbapenemase in Qatar. New Microbes New Infect..

[5] Khan F.Y., Abukhattab M., AbuKamar M., Anand D. (2014). Adult K Lebsiella Pneumoniae Meningitis in Qatar: Clinical Pattern of Ten Cases. Asian Pac. J. Trop. Biomed..

[6] Al Samawi M.S., Khan F.Y., Eldeeb Y., Almaslamani M., Alkhal A., Alsoub H., Ghadban W., Howady F., Hashim S. (2016). *Acinetobacter* Infections among Adult Patients in Qatar: A 2-Year Hospital-Based Study. Can. J. Infect. Dis. Med. Microbiol..

[7] Khan F.Y., Elshafie S.S., Almaslamani M., Abu-Khattab M., El Hiday A.H., Errayes M., Almaslamani E. (2010). Epidemiology of Bacteraemia in Hamad General Hospital, Qatar: A One Year Hospital-Based Study. Travel Med. Infect. Dis..

[8] John Albert M., Bulach D., Alfouzan W., Izumiya H., Carter G., Alobaid K., Alatar F., Sheikh A.R., Poirel L. (2019). Non-Typhoidal Salmonella Blood Stream Infection in Kuwait: Clinical and Microbiological Characteristics. PLoS Negl. Trop. Dis..

[9] Alfouzan W., Ahmad S., Dhar R., Asadzadeh M., Almerdasi N., Abdo N.M., Joseph L., de Groot T., Alali W.Q., Khan Z. (2020). Molecular Epidemiology of Candida Auris Outbreak in a Major Secondary-Care Hospital in Kuwait. J. Fungi.

[10] Jamal W., Rotimi V.O., Albert M.J., Khodakhast F., Nordmann P., Poirel L. (2013). High Prevalence of VIM-4 and NDM-1 Metallo-β-Lactamase among Carbapenem-Resistant Enterobacteriaceae. J. Med. Microbiol..

[11] Khan Z., Ahmad S., Al-Sweih N., Joseph L., Alfouzan W., Asadzadeh M. (2018). Increasing Prevalence, Molecular Characterization and Antifungal Drug Susceptibility of Serial Candida Auris Isolates in Kuwait. PLoS ONE.

[12] Jamal W., Al Roomi E., AbdulAziz L.R., Rotimi V.O. (2014). Evaluation of Curetis Unyvero, a Multiplex PCR-Based Testing System, for Rapid Detection of Bacteria and Antibiotic Resistance and Impact of the Assay on Management of Severe Nosocomial Pneumonia. J. Clin. Microbiol..

[13] Jamal W.Y., Albert M.J., Khodakhast F., Poirel L., Rotimi V.O. (2015). Emergence of New Sequence Type OXA-48 Carbapenemase-Producing *Enterobacteriaceae* in Kuwait. Microb. Drug Resist..

[14] Ibrahim M.M., Abuelmatty A.M., Mohamed G.H., Nasr M.A., Hussein A.K., Ebaed M.E.D., Sarhan H.A. (2018). Best Tigecycline Dosing for Treatment of Infections Caused by Multidrug-Resistant Pathogens in Critically Ill Patients with Different Body Weights. Drug Des. Dev. Ther..

[15] Babakir-Mina M., Othman N., Najmuldeen H.H., Noori C.K., Fatah C.F., Perno C.-F., Ciotti M. (2012). Antibiotic Susceptibility of Vancomyin and Nitrofurantoin in Staphylococcus Aureus Isolated from Burnt Patients in Sulaimaniyah, Iraqi Kurdistan. New Microbiol..

[16] Jaber A.A.S., Ibrahim B. (2019). Evaluation of Risk Factors Associated with Drug-Resistant Tuberculosis in Yemen: Data from Centres with High Drug Resistance. BMC Infect. Dis..

[17] Jaber A.A.S., Ibrahim B. (2019). Health-Related Quality of Life of Patients with Multidrug-Resistant Tuberculosis in Yemen: Prospective Study. Health Qual. Life Outcomes.

[18] Abd El Hafez M., Khalaf N.G., El Ahmady M., Abd El Aziz A., Hashim A.E.G. (2011). An Outbreak of Methicillin Resistant Staphylococcus EpidermiDis. among Neonates in a Hospital in Saudi Arabia. J. Infect. Dev. Ctries.

[19] Al Mayahi Z., Kamel S., Amer H., Beatty M. (2019). Outbreak of Colistin-Resistant Organisms at a Tertiary Hospital in Riyadh, Saudi Arabia, 2016. Pan Afr. Med. J..

[20] Khan U., Huerga H., Khan A.J., Mitnick C.D., Hewison C., Varaine F., Bastard M., Rich M., Franke M.F., Atwood S. (2019). The EndTB Observational Study Protocol: Treatment of MDR-TB with Bedaquiline or Delamanid Containing Regimens. BMC Infect. Dis..

[21] Al-Ghafli H., Kohl T.A., Merker M., Varghese B., Halees A., Niemann S., Al-Hajoj S. (2018). Drug-Resistance Profiling and Transmission Dynamics of Multidrug-Resistant Mycobacterium Tuberculosis in Saudi Arabia Revealed by Whole Genome Sequencing. Infect. Drug Resist..

[22] Al-Jindan R., Al-Eraky D. (2021). Two Cases of the Emerging Candida Auris in a University Hospital from Saudi Arabia. Saudi J. Med. Med. Sci..

[23] Alhussain F.A., Yenugadhati N., Al Eidan F.A., Al Johani S., Badri M. (2021). Risk Factors, Antimicrobial Susceptibility Pattern and Patient Outcomes of Pseudomonas Aeruginosa Infection: A Matched Case-Control Study. J. Infect. Public Health.

[24] AlJindan R., AlEraky D.M., Mahmoud N., Abdalhamid B., Almustafa M., AbdulAzeez S., Borgio J.F. (2020). Drug Resistance-Associated Mutations in ERG11 of Multidrug-Resistant Candida Auris in a Tertiary Care Hospital of Eastern Saudi Arabia. J. Fungi.

[25] Almaghrabi R.S., Albalawi R., Mutabagani M., Atienza E., Aljumaah S., Gade L., Forsberg K., Litvintseva A., Althawadi S. (2020). Molecular Characterisation and Clinical Outcomes of *Candida Auris* Infection: Single-centre Experience in Saudi Arabia. Mycoses.

[26] Bosaeed M., Ahmad A., Alali A., Mahmoud E., Alswidan L., Alsaedy A., Aljuhani S., Alalwan B., Alshamrani M., Alothman A. (2020). Experience With Ceftolozane-Tazobactam for the Treatment of Serious *Pseudomonas Aeruginosa* Infections in Saudi Tertiary Care Center. Infect. Dis..

[27] Aljadani R., Ahmed A.E., AL-Jahdali H. (2019). Tuberculosis Mortality and Associated Factors at King Abdulaziz Medical City Hospital. BMC Infect. Dis..

[28] Almangour T.A., Alenazi B., Ghonem L., Alhifany A.A., Aldakheel B.A., Alruwaili A. (2020). Inhaled Colistin for the Treatment of Nosocomial Pneumonia Due to Multidrug-Resistant Gram-Negative Bacteria: A Real-Life Experience in Tertiary Care Hospitals in Saudi Arabia. Saudi Pharm. J..

[29] Abdalhamid B., Almaghrabi R., Althawadi S., Omrani A. (2018). First Report of Candida Auris Infections from Saudi Arabia. J. Infect. Public Health.

[30] Alraddadi B.M., Saeedi M., Qutub M., Alshukairi A., Hassanien A., Wali G. (2019). Efficacy of Ceftazidime-Avibactam in the Treatment of Infections Due to Carbapenem-Resistant Enterobacteriaceae. BMC Infect. Dis..

[31] Balkhy H., El-Saed A., Maghraby R., Khan R., Rishu A., Al-Dorzi H., Arabi Y. (2014). Drug-Resistant Ventilator Associated Pneumonia in a Tertiary Care Hospital in Saudi Arabia. Ann. Thorac. Med..

[32] Älgå A., Wong S., Shoaib M., Lundgren K., Giske C.G., von Schreeb J., Malmstedt J. (2018). Infection with High Proportion of Multidrug-Resistant Bacteria in Conflict-Related Injuries is Associated with Poor Outcomes and Excess Resource Consumption: A Cohort Study of Syrian Patients Treated in Jordan. BMC Infect. Dis..

[33] Al-lawama M., Aljbour H., Tanash A., Badran E. (2016). Intravenous Colistin in the Treatment of Multidrug-Resistant Acinetobacter in Neonates. Ann. Clin. Microbiol. Antimicrob..

[34] Almomani B.A., McCullough A., Gharaibeh R., Samrah S., Mahasneh F. (2015). Incidence and Predictors of 14-Day Mortality in Multidrug-Resistant Acinetobacter Baumannii in Ventilator-Associated Pneumonia. J. Infect. Dev. Ctries.

[35] Abd El Ghany M., Sharaf H., Al-agamy M.H., Shibl A., Hill-Cawthorne G.A., Hong P.-Y. (2018). Genomic Characterization of NDM-1 and 5, and OXA-181 Carbapenemases in Uropathogenic Escherichia coli Isolates from Riyadh, Saudi Arabia. PLoS ONE.

[36] Abdel-Halim H., Al Dajani A., Abdelhalim A., Abdelmalek S. (2019). The Search of Potential Inhibitors of the AcrAB–TolC System of Multidrug-Resistant Escherichia coli: An in Silico Approach. Appl. Microbiol. Biotechnol..

[37] Abed A.R., Khudhair A.M., Hussein I.M. (2020). Effects of Misuse of Antibiotics on the Resistance of Escherichia coli Isolated from the Intestines of Broiler Chickens. Int. J. Drug Deliv. Technol..

[38] Ahmad S., Al-Mutairi N.M., Mokaddas E. (2012). Variations in the Occurrence of Specific RpoB Mutations in Rifampicin- Resistant Mycobacterium Tuberculosis Isolates from Patients of Different Ethnic Groups in Kuwait. Indian J. Med. Res..

[39] Ahmad Hamad P., Khadija K.M. (2019). Prevalence of blaTEM, blaSHV, and blaCTX-M Genes among ESBL-Producing Klebsiella pneumoniae and Escherichia coli Isolated from Thalassemia Patients in Erbil, Iraq. Mediterr. J. Hematol. Infect. Dis..

[40] Ahmed M.U., Farooq R., Hawashim N.A., Ahmed M., Yiannakou N., Sayeed F., Sayed A.R., Lutfullah S. (2015). Sensitive, Resistant and Multi-Drug Resistant Acinetobacter Baumanii at Saudi Arabia Hospital Eastern Region. Pak. J. Pharm. Sci..

[41] Al Johani S.M., Akhter J., Balkhy H., El-Saed A., Younan M., Memish Z. (2010). Prevalence of Antimicrobial Resistance among Gram-Negative Isolates in an Adult Intensive Care Unit at a Tertiary Care Center in Saudi Arabia. Ann. Saudi Med..

[42] Al Wutayd O., Al Nafeesah A., Adam I., Babikir I. (2018). The Antibiotic Susceptibility Patterns of Uropathogens Isolated in Qassim, Saudi Arabia. J. Infect. Dev. Ctries.

[43] Alaa A.R., Solhan M.A., Lalan R.M., Hiwa L.I., Mhamad N.R., Niga K.M., Awat J.N., Salar A.I., Brwa H.Q., Daryan K.K.H. (2020). The antibiotic resistance pattern and molecular characterization of blactx and blatem genes of e. Coli isolated from different hosts based on the rate of antibiotic consumption in Sulaymaniyah/Iraq. Appl. Ecol. Environ. Res..

[44] Alanazi M.Q., Alqahtani F.Y., Aleanizy F.S. (2018). An Evaluation of E. coli in Urinary Tract Infection in Emergency Department at KAMC in Riyadh, Saudi Arabia: Retrospective Study. Ann. Clin. Microbiol. Antimicrob..

[45] Alatoom A., Elsayed H., Lawlor K., AbdelWareth L., El-Lababidi R., Cardona L., Mooty M., Bonilla M.-F., Nusair A., Mirza I. (2017). Comparison of Antimicrobial Activity between Ceftolozane–Tazobactam and Ceftazidime–Avibactam against Multidrug-Resistant Isolates of Escherichia coli, Klebsiella Pneumoniae, and Pseudomonas Aeruginosa. Int. J. Infect. Dis..

[46] Alavudeen S.S., Vigneshwaran E., Asiri S.A.A., Alahmari M.H.A., Mohammed M.A., Algahtani T., Khan N.A. (2017). Distribution of Multi-Resistant Bacterial Isolates from Clinical Specimens in a Hospital Environment of Kingdom of Saudi Arabia. J. Young Pharm..

[47] Al-Ayed M.S.Z., Asaad A.M., Qureshi M.A., Attia H.G., AlMarrani A.H. (2016). Antibacterial Activity of *Salvadora Persica* L. (Miswak) Extracts against Multidrug Resistant Bacterial Clinical Isolates. Evid.-Based Complementary Altern. Med..

[48] Albukhari T.A.M., Nafady-Hego H., Elgendy H., Abd Elmoneim H.M., Nafady A., Alzahrani A.M. (2019). Analysis of Bacterial and Fungal Infections after Cytoreduction Surgery and Hyperthermic Intraperitoneal Chemotherapy: An Observational Single-Centre Study. Int. J. Microbiol..

[49] Al-Guranie D.R., Al-Mayahie S.M. (2020). Prevalence of E. coli ST131 among Uropathogenic E. coli Isolates from Iraqi Patients in Wasit Province, Iraq. Int. J. Microbiol..

[50] Ali F.A., Hussen B.M., Zaki S.M. (2020). Molecular detection of blactx-m gene among pseudomonas aeruginosa strains isolated from different clinical samples in erbil city. Ann. Trop. Med. Public Health.

[51] Aljanaby A.A.J. (2018). Antibacterial Activity of an Aqueous Extracts of Alkanna Tinctoria Roots against Drug Resistant Aerobic Pathogenic Bacteria Isolated from Patients with Burns Infections. Russ. Open Med. J..

[52] Aljanaby A.A.J., Tuwaij N.S.S., Al-khilkhali H.J.B. (2018). Antimicrobial Susceptibility Patterns of Klebsiella Pneumoniae Isolated from Older Smokers and Non-Smokers of Inpatients in Intensive Care Unit Infected with Chronic Pneumonia in AL-Najaf Hospital, Iraq. J. Pharm. Sci..

[53] Alm’amoori K., Hadi Z., Almohana A. (2020). Molecular Investigation of Plasmid–Mediated Quinolone Resistant Genes among Aminoglycoside-Resistant Uropathogenic Escherichia coli Isolates from Babylon Hospitals, Iraq. Indian J. Forensic Med. Toxicol..

[54] Al-Mayahie S.M. (2013). Phenotypic and Genotypic Comparison of ESBL Production by Vaginal Escherichia coli Isolates from Pregnant and Non-Pregnant Women. Ann. Clin. Microbiol. Antimicrob..

[55] Al-Mulla N., Elshafie S.S., Janahi M., Al-Nasser A., Chandra P., Taj-Aldeen S.J. (2014). Bacterial Bloodstream Infections and Antimicrobial Susceptibility Pattern in Pediatric Hematology/Oncology Patients after Anticancer Chemotherapy. Infect. Drug Resist..

[56] AlOtair H.A., Hussein M.A., Elhoseny M.A., Alzeer A.H., Khan M.F. (2015). Severe Pneumonia Requiring ICU Admission: Revisited. J. Taibah Univ. Med. Sci..

[57] Alqasim A., Abu Jaffal A., Alyousef A.A. (2018). Prevalence of Multidrug Resistance and Extended-Spectrum *β* -Lactamase Carriage of Clinical Uropathogenic *Escherichia coli* Isolates in Riyadh, Saudi Arabia. Int. J. Microbiol..

[58] Alshareef H., Alfahad W., Albaadani A., Alyazid H., Talib R.B. (2020). Impact of Antibiotic De-Escalation on Hospitalized Patients with Urinary Tract Infections: A Retrospective Cohort Single Center Study. J. Infect. Public Health.

[59] Alshukairi A.N., Moalim H.M., Alsaedi A., Almansouri W.Y., Al-Zahrani M., Aljuaid A., Alraddadi B.M., Altorkistani H.H., Alrajhi A.A., Al-Hajoj S.A. (2020). Family Cluster of Multi-Drug Resistant Tuberculosis in Kingdom of Saudi Arabia. J. Infect. Public Health.

[60] Altalhi A.D., Gherbawy Y.A., Hassan S.A. (2010). Antibiotic Resistance in *Escherichia coli* Isolated from Retail Raw Chicken Meat in Taif, Saudi Arabia. Foodborne Pathog. Dis..

[61] Awayid H.S., Sahar B.R., Jalil I.S., Nouman K.T. (2019). Immunization against Multi Drug Resistance Uropathogenic *E. coli* Isolate from Urinary Tract Infection in Pregnancy. Res. J. Pharm. Technol..

[62] Azab K.S.M., Abdel-Rahman M.A., El-Sheikh H.H., Azab E., Gobouri A.A., Farag M.M.S. (2021). Distribution of Extended-Spectrum β-Lactamase (ESBL)-Encoding Genes among Multidrug-Resistant Gram-Negative Pathogens Collected from Three Different Countries. Antibiotics.

[63] Azim N.S.A., Al-Harbi M.A., Al-Zaban M.I., Nofal M.Y., Somily A.M. (2019). Prevalence and Antibiotic Susceptibility among Gram Negative Bacteria Isolated from Intensive Care Units at a Tertiary Care Hospital in Riyadh, Saudi Arabia. J. Pure Appl. Microbiol..

[64] Badran E.F., Din R.A.Q., Shehabi A.A. (2016). Low Intestinal Colonization of Escherichia coli Clone ST131 Producing CTX-M-15 in Jordanian Infants. J. Med. Microbiol..

[65] Balkhy H.H., El-Saed A., Alshamrani M.M., Alsaedi A., Al Nasser W., El Gammal A., Aljohany S.M., Almunif S., Arabi Y., Alqahtani S. (2020). Ten-Year Resistance Trends in Pathogens Causing Healthcare-Associated Infections; Reflection of Infection Control Interventions at a Multi-Hospital Healthcare System in Saudi Arabia, 2007–2016. Antimicrob. Resist. Infect. Control.

[66] Balkhy H.H., El-Saed A., Alshamrani M.M., Alsaedi A., Nasser W.A., Gammal A.E., Aljohany S.M., Arabi Y., Alqahtani S., Bonnie H.B. (2020). High Burden of Resistant Gram Negative Pathogens Causing Device-Associated Healthcare Infections in a Tertiary Care Setting in Saudi Arabia, 2008–2016. J. Glob. Antimicrob. Resist..

[67] Bindayna K.M., Khanfar H.S., Senok A.C., Botta G.A. (2010). Predominance of CTX-M Genotype among Extended Spectrum Beta Lactamase Isolates in a Tertiary Hospital in Saudi Arabia. Saudi Med. J..

[68] Burjaq S.Z., Shehabi A. (2013). Fresh Leafy Green Vegetables Associated with Multidrug Resistant E. coli. Int. ARABIC J. Antimicrob. Agents.

[69] Darwish R.M., Aburjai T.A. (2010). Effect of Ethnomedicinal Plants Used in Folklore Medicine in Jordan as Antibiotic Resistant Inhibitors on Escherichia coli. BMC Complement Altern. Med..

[70] Dashti A.A., Vali L., El-Shazly S., Jadaon M.M. (2014). The Characterization and Antibiotic Resistance Profiles of Clinical Escherichia coli O25b-B2-ST131 Isolates in Kuwait. BMC Microbiol..

[71] Dawood W.S. (2020). Molecular and Susceptibility Study of Antibiotic Resistance Genes in E. coli Isolated from Selected Iraqi Patients. Syst. Rev. Pharm..

[72] El-Ghareeb W.R., Abdel-Raheem S.M., Al-Marri T.M., Alaql F.A., Fayez M.M. (2020). Isolation and Identification of Extended Spectrum β-Lactamases (ESBLs) Escherichia coli from Minced Camel Meat in Eastern Province, Saudi Arabia. Thai J. Vet. Med..

[73] El-Saed A., Balkhy H.H., Alshamrani M.M., Aljohani S., Alsaedi A., Al Nasser W., El Gammal A., Almohrij S.A., Alyousef Z., Almunif S. (2020). High Contribution and Impact of Resistant Gram Negative Pathogens Causing Surgical Site Infections at a Multi-Hospital Healthcare System in Saudi Arabia, 2007–2016. BMC Infect. Dis..

[74] Elsohaby I., Samy A., Elmoslemany A., Alorabi M., Alkafafy M., Aldoweriej A., Al-Marri T., Elbehiry A., Fayez M. (2021). Migratory Wild Birds as a Potential Disseminator of Antimicrobial-Resistant Bacteria around Al-Asfar Lake, Eastern Saudi Arabia. Antibiotics.

[75] Eltai N.O., Abdfarag E.A., Al-Romaihi H., Wehedy E., Mahmoud M.H., Alawad O.K., Al-Hajri M.M., Al Thani A.A., Yassine H.M. (2017). Antibiotic Resistance Profile of Commensal Escherichia coli Isolated from Broiler Chickens in Qatar. J. Food Prot..

[76] Eltai N.O., Al Thani A.A., Al Hadidi S.H., Al Ansari K., Yassine H.M. (2020). Antibiotic Resistance and Virulence Patterns of Pathogenic Escherichia coli Strains Associated with Acute Gastroenteritis among Children in Qatar. BMC Microbiol..

[77] Fadlelmula A., Al-Hamam N.A., Al-Dughaym A.M. (2016). A Potential Camel Reservoir for Extended-Spectrum β-Lactamase-Producing Escherichia coli Causing Human Infection in Saudi Arabia. Trop. Anim. Health Prod..

[78] Garcell H.G., Arias A.V., Pancorbo Sandoval C.A., García E.G., Valle Gamboa M.E., Sado A.B., Alfonso Serrano R.N. (2017). Incidence and Etiology of Surgical Site Infections in Appendectomies: A 3-Year Prospective Study. Oman Med. J..

[79] Ghanem B., Haddadin R.N. (2018). Multiple Drug Resistance and Biocide Resistance in Escherichia coli Environmental Isolates from Hospital and Household Settings. Antimicrob. Resist. Infect. Control.

[80] Haddadin R.N., Assaf A.M., Homsi A., Collier P.J., Shehabi A. (2019). Investigating Possible Association between Multidrug Resistance and Isolate Origin with Some Virulence Factors of *Escherichia coli* Strains Isolated from Infant Faeces and Fresh Green Vegetables. J. Appl. Microbiol..

[81] Hameed T., Al Nafeesah A., Chishti S., Al Shaalan M., Al Fakeeh K. (2019). Community-Acquired Urinary Tract Infections in Children: Resistance Patterns of Uropathogens in a Tertiary Care Center in Saudi Arabia. Int. J. Pediatrics Adolesc. Med..

[82] Hammoudi A. (2019). Antibiotics Susceptibility Pattern of Some Enterobacteriaceae Isolates from Different Clinical Infectious Sources. Int. J. Res. Pharm. Sci..

[83] Hassan H., Abdalhamid B. (2014). Molecular Characterization of Extended-Spectrum Beta-Lactamase Producing Enterobacteriaceae in a Saudi Arabian Tertiary Hospital. J. Infect. Dev. Ctries.

[84] Huang X.-Z., Frye J.G., Chahine M.A., Glenn L.M., Ake J.A., Su W., Nikolich M.P., Lesho E.P. (2012). Characteristics of Plasmids in Multi-Drug-Resistant Enterobacteriaceae Isolated during Prospective Surveillance of a Newly Opened Hospital in Iraq. PLoS ONE.

[85] Ibrahim R.A., Cryer T.L., Lafi S.Q., Basha E.-A., Good L., Tarazi Y.H. (2019). Identification of Escherichia coli from Broiler Chickens in Jordan, Their Antimicrobial Resistance, Gene Characterization and the Associated Risk Factors. BMC Vet. Res..

[86] Ibrahim I., Al- Shwaikh R., Ismaeil M. (2014). Virulence and Antimicrobial Resistance of Escherichia coli Isolated from Tigris River and Children Diarrhea. Infect. Drug Resist..

[87] Ibrahim M.E., Bilal N.E., Hamid M.E. (2014). Comparison of Phenotypic Characteristics and Antimicrobial Resistance Patterns of Clinical Escherichia coli Collected From Two Unrelated Geographical Areas. GJHS.

[88] Jaloob Aljanaby A.A., Aljanaby I.A.J. (2018). Antimicrobial Sensitivity Pattern of Pathogenic Bacteria Isolated from Older Women with Asymptomatic Bacteriuria. Biomed. Res..

[89] Karomi A.S.A. (2020). Screening Study for Some Strains of E. coli Collected from Five Regions in Kurdistan-Iraq for Its Sensitivity, Resistance and MDR against Thirteen Antibiotics. Med. Leg. Update.

[90] Khairy G.A., Kambal A.M., Al-Dohayan A.A., Al-Shehri M.Y., Zubaidi A.M., Al-Naami M.Y., AlSaif F.A., Al-Obaid O.A., Al-Saif A.A., El-Farouk O.Y. (2011). Surgical Site Infection in a Teaching Hospital: A Prospective Study. J. Taibah Univ. Med. Sci..

[91] Majeed H.T., Aljanaby A.A.J. (2019). Antibiotic Susceptibility Patterns and Prevalence of Some Extended Spectrum Beta- Lactamases Genes in Gram-Negative Bacteria Isolated from Patients Infected with Urinary Tract Infections in Al-Najaf City, Iraq. Avicenna J. Med. Biotechnol..

[92] Marie M.A., John J., Krishnappa L.G., Gopalkrishnan S. (2013). Molecular Characterization of the β-Lactamases in *Escherichia coli* and *Klebsiella Pneumoniae* from a Tertiary Care Hospital in Riyadh, Saudi Arabia: β-Lactamases in *Enterobacteriaceae*. Microbiol. Immunol..

[93] Mazi W., Begum Z., Abdulla D., Hesham A., Maghari S., Assiri A., Senok A. (2014). Central Line–Associated Bloodstream Infection in a Trauma Intensive Care Unit: Impact of Implementation of Society for Healthcare Epidemiology of America/Infectious Diseases Society of America Practice Guidelines. Am. J. Infect. Control.

[94] Memish Z.A., Assiri A., Almasri M., Roshdy H., Hathout H., Kaase M., Gatermann S.G., Yezli S. (2015). Molecular Characterization of Carbapenemase Production Among Gram-Negative Bacteria in Saudi Arabia. Microb. Drug Resist..

[95] Mutti M., Sonnevend Á., Pál T., Junttila S., Ekker H., Galik B., Gyenesei A., Nagy G., Nagy E., Szijártó V. (2018). Complete Genome Sequence of *Escherichia coli* 81009, a Representative of the Sequence Type 131 C1-M27 Clade with a Multidrug-Resistant Phenotype. Genome Announc..

[96] Nairoukh Y.R., Mahafzah A.M., Irshaid A., Shehabi A.A. (2018). Molecular Characterization of Multidrug Resistant Uropathogenic *E. coli* Isolates from Jordanian Patients. Open Microbiol. J..

[97] Narchi H., Al-Hamdani M. (2010). Uropathogen Resistance to Antibiotic Prophylaxis in Urinary Tract Infections. Microb. Drug Resist..

[98] Nimri L., Abu AL- Dahab F., Batchoun R. (2014). Foodborne Bacterial Pathogens Recovered from Contaminated Shawarma Meat in Northern Jordan. J. Infect. Dev. Ctries.

[99] Nimri L., Samara H., Batchoun R. (2011). Detection of Mutations Associated with Multidrug-Resistant *Mycobacterium Tuberculosis* Clinical Isolates. FEMS Immunol. Med. Microbiol..

[100] Obaidat M.M. (2020). Prevalence and Antimicrobial Resistance of Listeria Monocytogenes, Salmonella Enterica and Escherichia coli O157:H7 in Imported Beef Cattle in Jordan. Comp. Immunol. Microbiol. Infect. Dis..

[101] Pál T., Ghazawi A., Darwish D., Villa L., Carattoli A., Hashmey R., Aldeesi Z., Jamal W., Rotimi V., Al-Jardani A. (2017). Characterization of NDM-7 Carbapenemase-Producing *Escherichia coli* Isolates in the Arabian Peninsula. Microb. Drug Resist..

[102] Radwan Ali M., Khudhair A.M. (2018). Detection of Colony Adhesion Factors and Genetic Background of Adhesion Genes Among Multidrug-Resistant Uropathogenic Escherichia coli Isolated in Iraq. J. Pure Appl. Microbiol..

[103] Rana M.A., Abd El Rahaman B., Mady A.F., Al Odat M., Al Harthy A., Ramadan O.E.S., Mumtaz S.A., Omrani A.S. (2014). Intra-Pleural Colistin Methanesulfonate Therapy for Pleural Infection Caused by Carbapenem-Resistant Acinetobacter Baumannii: A Successful Case Report. Infect. Dis. Rep..

[104] Salah M., Badran E., Shehabi A. (2014). High Incidence of Multidrug Resistant Escherichia coli Producing CTX-M-Type ESBLs Colonizing the Intestine of Jordanian Infants. Int. Arab. J. Antimicrob. Agents.

[105] Shobrak M.Y., Hassan S.A., Stiévenart C., El-Deeb B.A., Gherbawy Y.A. (2013). Prevalence and Antibiotic Resistance Profile of Intestinal Bacteria Isolated from Captive Adult Houbara Bustards ( ) Exposed to Natural Weather Conditions in Saudi Arabia. Escherichia coli. Glob. Vet..

[106] Sonnevend A., Yahfoufi N., Ghazawi A., Jamal W., Rotimi V., Pal T. (2017). Contribution of Horizontal Gene Transfer to the Emergence of VIM-4 Carbapenemase Producer Enterobacteriaceae in Kuwait. Infect. Drug Resist..

[107] Taha M.M.E., Homeida H.E., Dafalla O.M.E., Abdelwahab S.I. (2018). Multidrug Resistance, Prevalence and Phylogenetic Analysis of Genes Encoding Class II and III Integrons in Clinically Isolated Escherichia coli. Cell. Mol. Biol..

[108] Taher I., Almaeen A., Aljourfi H., Bohassan E., Helmy A., El-Masry E., Saleh B., Aljaber N. (2020). Surveillance of Antibiotic Resistance among Uropathogens in Aljouf Region Northern Saudi Arabia. Iran. J. Microbiol..

[109] Tarazi Y.H., Abu-Basha E.A., Ismail Z.B., Tailony R.A. (2020). In Vitro and in Vivo Efficacy Study of Cefepime, Doripenem, Tigecycline, and Tetracycline against Extended-Spectrum Beta-Lactamases Escherichia coli in Chickens. Vet. World.

[110] Thani A.S.B. (2019). Characterization of Previously Identified Novel DNA Fragment Associated with Pathogenicity Island III536 Reveals New Bla Gene. Infect. Genet. Evol..

[111] Saeed N.K., Kambal A.M., El-Khizzi N.A. (2010). Antimicrobial-Resistant Bacteria in a General Intensive Care Unit in Saudi Arabia. Saudi Med. J..

[112] Ahmad S., Mokaddas E., Al-Mutairi N., Eldeen H.S., Mohammadi S. (2016). Discordance across Phenotypic and Molecular Methods for Drug Susceptibility Testing of Drug-Resistant Mycobacterium Tuberculosis Isolates in a Low TB Incidence Country. PLoS ONE.

[113] Ahmed M.M., Mohammed S.H., Nasurallah H.A.A., Ali M.M., Couvin D., Rastogi N. (2014). Snapshot of the Genetic Diversity of Mycobacterium Tuberculosis Isolates in Iraq. Int. J. Mycobacteriol..

[114] Ahmed-Abakur E., Saad Alnour T. (2019). Detection of Multidrug Resistant Mycobacterium Tuberculosis in Tabuk, Saudi Arabia, Using Genotype MTBDRplus. Int. J. Mycobacteriol..

[115] Al Mahbashi A.A., Mukhtar M.M., Mahgoub E.S. (2013). Molecular Typing of Mycobacterium spp. Isolates from Yemeni Tuberculosis Patients. East Mediterr. Health J..

[116] AL Qurainees G.I., Tufenkeji H.T. (2016). A Child with Complicated Mycobacterium Tuberculosis. Int. J. Pediatrics Adolesc. Med..

[117] Alateah S.M., Othman M.W., Ahmed M., Al Amro M.S., Al Sherbini N., Ajlan H.H. (2020). A Retrospective Study of Tuberculosis Prevalence amongst Patients Attending a Tertiary Hospital in Riyadh, Saudi Arabia. J. Clin. Tuberc. Other Mycobact. Dis..

[118] Al-Hajoj S., Varghese B., Shoukri M.M., Al-Omari R., Al-Herbwai M., AlRabiah F., Alrajhi A.A., Abuljadayel N., Al-Thawadi S., Zumla A. (2013). Epidemiology of Antituberculosis Drug Resistance in Saudi Arabia: Findings of the First National Survey. Antimicrob. Agents Chemother..

[119] Al-Hajoj S., Shoukri M., Memish Z., AlHakeem R., AlRabiah F., Varghese B. (2015). Exploring the Sociodemographic and Clinical Features of Extrapulmonary Tuberculosis in Saudi Arabia. PLoS ONE.

[120] Ali M., Howady F., Munir W., Karim H., Al-Suwaidi Z., Al-Maslamani M., Alkhal A., Elmaki N., Ziglam H. (2020). Drug-Resistant Tuberculosis: An Experience from Qatar. Libyan J. Med..

[121] Ali Chaudhry L., Rambhala N., Al-Shammri A.S., Al-Tawfiq J.A. (2011). Patterns of Antituberculous Drug Resistance in Eastern Saudi Arabia: A 7-Year Surveillance Study from 1/2003 to 6/2010. J. Epidemiol. Glob. Health.

[122] Al-Mutairi N.M., Ahmad S., Mokaddas E., Eldeen H.S., Joseph S. (2019). Occurrence of Disputed RpoB Mutations among Mycobacterium Tuberculosis Isolates Phenotypically Susceptible to Rifampicin in a Country with a Low Incidence of Multidrug-Resistant Tuberculosis. BMC Infect. Dis..

[123] Al-Mutairi N.M., Ahmad S., Mokaddas E. (2011). First Report of Molecular Detection of Fluoroquinolone Resistance-Associated GyrA Mutations in Multidrug-Resistant Clinical Mycobacterium Tuberculosis Isolates in Kuwait. BMC Res. Notes.

[124] Al-Mutairi N.M., Ahmad S., Mokaddas E. (2018). Molecular Screening Versus Phenotypic Susceptibility Testing of Multidrug-Resistant *Mycobacterium Tuberculosis* Isolates for Streptomycin and Ethambutol. Microb. Drug Resist..

[125] Al-Mutairi N.M., Ahmad S., Mokaddas E.M. (2020). Correction to: Molecular Characterization of Multidrug-Resistant Mycobacterium Tuberculosis (MDR-TB) Isolates Identifies Local Transmission of Infection in Kuwait, a Country with a Low Incidence of TB and MDR-TB. Eur. J. Med. Res..

[126] Al-Rubaye D.S., Henihan G., Al-Abasly A.K.A., Seagar A.-L., Al-Attraqchi A.A.F., Schulze H., Hashim D.S., Kamil J.K., Laurenson I.F., Bachmann T.T. (2016). Genotypic Assessment of Drug-Resistant Tuberculosis in Baghdad and Other Iraqi Provinces Using Low-Cost and Low-Density DNA Microarrays. J. Med. Microbiol..

[127] Altuwaijri T.A., Alhindi G.K., Al-Qattan N.M., Alkharashi S.K., Somily A.M., Altoijry A.H. (2020). Occurrence of Venous Thromboembolism in Hospitalized Patients with Tuberculosis in Saudi Arabia: A Retrospective Cohort Study. Int. J. Mycobacteriol..

[128] Alyamani E.J., Marcus S.A., Ramirez-Busby S.M., Hansen C., Rashid J., El-kholy A., Spalink D., Valafar F., Almehdar H.A., Jiman-Fatani A.A. (2019). Genomic Analysis of the Emergence of Drug-Resistant Strains of Mycobacterium Tuberculosis in the Middle East. Sci. Rep..

[129] Al-Zarouni M., Dash N., Al Ali M., Al-Shehhi F., Panigrahi D. (2010). Tuberculosis and MDR-TB in the Northern Emirates of United Arab Emirates: A 5-Year Study. Southeast Asian J. Trop Med. Public Health.

[130] Asaad A.M., Alqahtani J.M. (2012). Primary Anti-Tuberculous Drugs Resistance of Pulmonary Tuberculosis in Southwestern Saudi Arabia. J. Infect. Public Health.

[131] El Mahalli A.A., Al-Qahtani M.F. (2015). Predictors of Drug Resistance in Tuberculosis Patients in the Eastern Province, Saudi Arabia. J. Egypt. Public Health Assoc..

[132] Flaifel D.K., Al-Azawi I.H. (2020). The Role of IL-6 Gene Polymorphism in Multidrug-Resistant Tuberculosis Patients in Iraq. Int. J. Drug Deliv. Technol..

[133] Habous M., Elimam M., AlDabal L., Chidambaran B., AlDeesi Z. (2020). Pattern of Primary Tuberculosis Drug Resistance and Associated Risk Factors at Dubai Health Authority in Dubai. Int. J. Mycobacteriol..

[134] Kareem P.A., Alsammak E.G.H., Abdullah Y.J., Bdaiwi Q.M. (2019). Estimation of Antibacterial Activity of Zinc Oxide, Titanium Dioxide, and Silver Nanoparticles against Multidrug-Resistant Bacteria Isolated from Clinical Cases in Amara City, Iraq. Drug Invent. Today.

[135] Elhassan M.M., Hemeg H.A., Elmekki M.A., Turkistani K.A., Abdul-Aziz A.A. (2017). Burden of Multidrug Resistant Mycobacterium Tuberculosis Among New Cases in Al-Madinah Al-Monawarah, Saudi Arabia. Infect. Disord.-Drug Targets.

[136] Merza M.A., Farnia P., Salih A.M., Masjedi M.R., Velayati A.A. (2011). First Insight into the Drug Resistance Pattern of Mycobacterium Tuberculosis in Dohuk, Iraq: Using Spoligotyping and MIRU-VNTR to Characterize Multidrug Resistant Strains. J. Infect. Public Health.

[137] Mokaddas E., Ahmad S., Eldeen H.S., Al-Mutairi N. (2015). Discordance between Xpert MTB/RIF Assay and Bactec MGIT 960 Culture System for Detection of Rifampin-Resistant Mycobacterium Tuberculosis Isolates in a Country with a Low Tuberculosis (TB) Incidence. J. Clin. Microbiol..

[138] Sambas M.F.M.K., Rabbani U., Al-Gethamy M.M.M., Surbaya S.H., Alharbi F.F.I., Ahmad R.G.A., Qul H.K.H., Nassar S.M.S., Maddah A.K.M.A., Darweesh B.A.K. (2020). Prevalence and Determinants of Multidrug-Resistant Tuberculosis in Makkah, Saudi Arabia. Infect. Drug Resist..

[139] Somily A.M., Naeem T., Habib H.A., Sarwar M.S., Kunimoto D.Y., Kambal A.M. (2014). Changing Epidemiology of Tuberculosis Detected by an 8-Year Retrospective Laboratory Study in a Tertiary Teaching Hospital in Central Saudi Arabia. Saudi Med. J..

[140] Varghese B., Supply P., Allix-Béguec C., Shoukri M., Al-Omari R., Herbawi M., Al-Hajoj S. (2013). Admixed Phylogenetic Distribution of Drug Resistant Mycobacterium Tuberculosis in Saudi Arabia. PLoS ONE.

[141] Varghese B., Al-Hajoj S. (2017). First Insight Into the Fluoroquinolone and Aminoglycoside Resistance of Multidrug-Resistant Mycobacterium Tuberculosis in Saudi Arabia. Am. J. Trop. Med. Hyg..

[142] Abulhasan Y.B., Abdullah A.A., Shetty S.A., Ramadan M.A., Yousef W., Mokaddas E.M. (2020). Health Care-Associated Infections in a Neurocritical Care Unit of a Developing Country. Neurocrit. Care.

[143] Ahmed S.S., Shariq A., Alsalloom A.A., Babikir I.H., Alhomoud B.N. (2019). Uropathogens and Their Antimicrobial Resistance Patterns: Relationship with Urinary Tract Infections. Int. J. Health Sci..

[144] Al Bshabshe A., Joseph M.R.P., Al Hussein A., Haimour W., Hamid M.E. (2016). Multidrug Resistance Acinetobacter Species at the Intensive Care Unit, Aseer Central Hospital, Saudi Arabia: A One Year Analysis. Asian Pac. J. Trop. Med..

[145] Al-Agamy M.H., Jeannot K., El-Mahdy T.S., Shibl A.M., Kattan W., Plésiat P., Courvalin P. (2017). First Detection of GES-5 Carbapenemase-Producing *Acinetobacter Baumannii* Isolate. Microb. Drug Resist..

[146] Alamri A.M., Alsultan A.A., Ansari M.A., Alnimr A.M. (2020). Biofilm-Formation in Clonally Unrelated Multidrug-Resistant Acinetobacter Baumannii Isolates. Pathogens.

[147] Al-Anazi K.A., Abdalhamid B., Alshibani Z., Awad K., Alzayed A., Hassan H., Alsayiegh M. (2012). *Acinetobacter Baumannii* Septicemia in a Recipient of an Allogeneic Hematopoietic Stem Cell Transplantation. Case Rep. Transplant..

[148] Al-Dabaibah N., Obeidat N., Shehabi A. (2012). Epidemiology Features of Acinetobacter Baumannii Colonizing Respiratory Tracts of ICU Patients. Int. Arab. J. Antimicrob. Agents.

[149] ALfadli M., EL-sehsah E.M., Ramadan M.A.-M. (2018). Risk Factors and Distribution of MDROs among Patients with Healthcare Associated Burn Wound Infection. Germs.

[150] Alhaddad M., AlBarjas A., Alhammar L., Al Rashed A., Badger-Emeka L. (2018). Molecular Characterization and Antibiotic Susceptibility Pattern of Acinetobacter Baumannii Isolated in Intensive Care Unit Patients in Al-Hassa, Kingdom of Saudi Arabia. Int. J. Appl. Basic Med. Res..

[151] Al-Hamad A., Pal T., Leskafi H., Abbas H., Hejles H., Alsubikhy F., Darwish D., Ghazawi A., Sonnevend A. (2020). Molecular Characterization of Clinical and Environmental Carbapenem Resistant Acinetobacter Baumannii Isolates in a Hospital of the Eastern Region of Saudi Arabia. J. Infect. Public Health.

[152] Alharbi A., Alshami I. (2015). In Vitro Effects of Tigecycline in Combination with Other Antimicrobials against Multidrug-Resistant Acinetobacter Baumannii Isolates. J. Pure Appl. Microbiol..

[153] AL-Harmoosh R., Jarallah E., AL-Shamari A. (2016). Coexistence of the BlaIMP and BlaSIM Genes in Clinical Isolates of Acinetobacter baumanniiIN Babylon Hospitals Iraq. Int. J. PharmTech Res..

[154] Ali H.M., Salem M.Z.M., El-Shikh M.S., Megeed A.A., Alogaibi Y.A., Talea I.A. (2017). Investigation of the Virulence Factors and Molecular Characterization of the Clonal Relations of Multidrug-Resistant Acinetobacter Baumannii Isolates. J. AOAC Int..

[155] Ali K.M., Al-Jaff B.M.A. (2021). Source and Antibiotic Susceptibility of Gram-Negative Bacteria Causing Superficial Incisional Surgical Site Infections. Int. J. Surg. Open.

[156] Ali M., Marie M., Gowda Krishnappa L., Alzahrani A.J., Mubaraki M.A., Alyousef A.A. (2015). A Prospective Evaluation of Synergistic Effect of Sulbactam and Tazobactam Combination with Meropenem or Colistin against Multidrug Resistant Acinetobacter Baumannii. Bosn. J. Basic Med. Sci..

[157] Aljindan R., Bukharie H., Alomar A., Abdalhamid B. (2015). Prevalence of Digestive Tract Colonization of Carbapenem-Resistant Acinetobacter Baumannii in Hospitals in Saudi Arabia. J. Med. Microbiol..

[158] AL-Kadmy I., Ali A., Salman I., Khazaal S. (2017). Molecular Characterization of Acinetobacter Baumannii Isolated from Iraqi Hospital Environment. New Microbes New Infect..

[159] Almaghrabi M.K., Joseph M.R.P., Assiry M.M., Hamid M.E. (2018). Multidrug-Resistant *Acinetobacter Baumannii*: An Emerging Health Threat in Aseer Region, Kingdom of Saudi Arabia. Can. J. Infect. Dis. Med. Microbiol..

[160] Almutairy R., Aljrarri W., Noor A., Elsamadisi P., Shamas N., Qureshi M., Ismail S. (2020). Impact of Colistin Dosing on the Incidence of Nephrotoxicity in a Tertiary Care Hospital in Saudi Arabia. Antibiotics.

[161] Al-Obeid S., Jabri L., Al-Agamy M., Al-Omari A., Shibl A. (2015). Epidemiology of Extensive Drug Resistant *Acinetobacter Baumannii* (XDRAB) at Security Forces Hospital (SFH) in Kingdom of Saudi Arabia (KSA). J. Chemother..

[162] Al-Ouqaili M.T.S., Jal’oot A.S., Badawy A.S. (2018). Identification of an OprD and Bla IMP Gene-Mediated Carbapenem Resistance in Acinetobacter Baumannii and Pseudomonas Aeruginosa among Patients with Wound Infections in Iraq. Asian J. Pharm..

[163] Al-Sweih N.A., Al-Hubail M.A., Rotimi V.O. (2011). Emergence of Tigecycline and Colistin Resistance in *Acinetobacter* Species Isolated from Patients in Kuwait Hospitals. J. Chemother..

[164] Al-Sweih N.A., Al-Hubail M., Rotimi V.O. (2012). Three Distinct Clones of Carbapenem-Resistant Acinetobacter Baumannii with High Diversity of Carbapenemases Isolated from Patients in Two Hospitals in Kuwait. J. Infect. Public Health.

[165] Aly M., Tayeb H.T., Al Johani S.M., Alyamani E.J., Aldughaishem F., Alabdulkarim I., Balkhy H.H. (2014). Genetic Diversity of OXA-51-like Genes among Multidrug-Resistant Acinetobacter Baumannii in Riyadh, Saudi Arabia. Eur. J. Clin. Microbiol. Infect. Dis..

[166] Aly M.M., Abu Alsoud N.M., Elrobh M.S., Al Johani S.M., Balkhy H.H. (2016). High Prevalence of the PER-1 Gene among Carbapenem-Resistant Acinetobacter Baumannii in Riyadh, Saudi Arabia. Eur. J. Clin. Microbiol. Infect. Dis..

[167] Azeez Z.F., Hatite Al-Daraghi W.A. (2019). Isolation of Lytic Acinetobacter Baumannii Phage VB_Acib_C_A10 from Iraq Pond Waters and Comparing Its Antibacterial Effect with Cefotaxime Antibiotic. Int. J. Pharm. Qual. Assur..

[168] Bakour S., Alsharapy S.A., Touati A., Rolain J.-M. (2014). Characterization of *Acinetobacter Baumannii* Clinical Isolates Carrying *Bla*
_OXA-23_ Carbapenemase and 16S RRNA Methylase *ArmA* Genes in Yemen. Microb. Drug Resist..

[169] Batarseh A., Al-Sarhan A., Maayteh M., Al-Khatirei S., Alarmouti M. (2015). Antibiogram of Multidrug Resistant Acinetobacter Baumannii Isolated from Clinical Specimens at King Hussein Medical Centre, Jordan: A Retrospective Analysis. Easter Mediterr. Health J..

[170] Conlon J.M., Ahmed E., Pal T., Sonnevend A. (2010). Potent and Rapid Bactericidal Action of Alyteserin-1c and Its [E4K] Analog against Multidrug-Resistant Strains of Acinetobacter Baumannii. Peptides.

[171] Conlon J.M., Mechkarska M., Arafat K., Attoub S., Sonnevend A. (2012). Analogues of the Frog Skin Peptide Alyteserin-2a with Enhanced Antimicrobial Activities against Gram-Negative Bacteria: ALYTESERIN-2: STRUCTURE-ACTIVITY. J. Pept. Sci..

[172] Conlon J.M., Sonnevend A., Pál T., Vila-Farrés X. (2012). Efficacy of Six Frog Skin-Derived Antimicrobial Peptides against Colistin-Resistant Strains of the Acinetobacter Baumannii Group. Int. J. Antimicrob. Agents.

[173] El-Ageery S.M., Abo-Shadi M.A., Elgendy A.M., Alghaithy A.A., Kandeel A.Y. (2011). The Role of Health Care Workers and Environment on Transmission of Methicillin–Resistant Staphylococcus Aureus among Patients in a Medical Intensive Care Unit in a Saudi Hospital. Appl. Microbiol..

[174] Ghaima K., Saadedin S., Jassim K. (2016). Prevalence of BlaOXA like Carbapenemase Genes in Multidrug Resistant Acinetobacter Baumannii Isolated from Burns and Wounds in Baghdad Hospitals. Res. J. Pharm. Biol. Chem. Sci..

[175] Gowda K.L., Marie M.A.M., Al-Sheikh Y.A., John J., Gopalkrishnan S., Shashidhar P.C., Dabwan K.H.M. (2014). A 6-Year Surveillance of Antimicrobial Resistance Patterns of *Acinetobacter Baumannii* Bacteremia Isolates from a Tertiary Care Hospital in Saudi Arabia during 2005–2010. Libyan J. Med..

[176] Hoang V.-T., Dao T.-L., Ly T.D.A., Gouriet F., Hadjadj L., Belhouchat K., Chaht K.L., Yezli S., Alotaibi B., Raoult D. (2021). Acquisition of Multidrug-Resistant Bacteria and Encoding Genes among French Pilgrims during the 2017 and 2018 Hajj. Eur. J. Clin. Microbiol. Infect. Dis..

[177] Kareem S.M., Al-Kadmy I.M.S., Al-Kaabi M.H., Aziz S.N., Ahmad M. (2017). Acinetobacter Baumannii Virulence Is Enhanced by the Combined Presence of Virulence Factors Genes Phospholipase C (PlcN) and Elastase (LasB). Microb. Pathog..

[178] Kusradze I., Diene S.M., Goderdzishvili M., Rolain J.-M. (2011). Molecular Detection of OXA Carbapenemase Genes in Multidrug-Resistant Acinetobacter Baumannii Isolates from Iraq and Georgia. Int. J. Antimicrob. Agents.

[179] Lopes B.S., Al-Agamy M.H., Ismail M.A., Shibl A.M., Al-Qahtani A.A., Al-Ahdal M.N., Forbes K.J. (2015). The Transferability of BlaOXA-23 Gene in Multidrug-Resistant Acinetobacter Baumannii Isolates from Saudi Arabia and Egypt. Int. J. Med. Microbiol..

[180] Mahdi L., Mahdi N., Al-kakei S., Musafer H., Al-Joofy I., Essa R., Zwain L., Salman I., Mater H., Al-Alak S. (2018). Treatment Strategy by Lactoperoxidase and Lactoferrin Combination: Immunomodulatory and Antibacterial Activity against Multidrug-Resistant Acinetobacter Baumannii. Microb. Pathog..

[181] Mazi W., Alshammari F., Yu J., Saeed A. (2020). A Descriptive Analysis of PVL-Positive Multidrug-Resistant Staphylococcus Aureus in Hospital-Associated Infections in Saudi Arabia. Bioinformation.

[182] Mechkarska M., Prajeep M., Radosavljevic G.D., Jovanovic I.P., Baloushi A.A., Sonnevend A., Lukic M.L., Conlon J.M. (2013). An Analog of the Host-Defense Peptide Hymenochirin-1B with Potent Broad-Spectrum Activity against Multidrug-Resistant Bacteria and Immunomodulatory Properties. Peptides.

[183] Muslim S.N., Al-Kadmy I.M.S., Auda I.G., Ali A.N.M., Al-Jubori S.S. (2018). A Novel Genetic Determination of a Lectin Gene in Iraqi Acinetobacter Baumannii Isolates and Use of Purified Lectin as an Antibiofilm Agent. J. AOAC Int..

[184] Nasser K., Mustafa A.S., Khan M.W., Purohit P., Al-Obaid I., Dhar R., Al-Fouzan W. (2018). Draft Genome Sequences of Six Multidrug-Resistant Clinical Strains of Acinetobacter Baumannii, Isolated at Two Major Hospitals in Kuwait. Genome Announc..

[185] Obeidat N., Jawdat F., Al-Bakri A.G., Shehabi A.A. (2014). Major Biologic Characteristics of Acinetobacter Baumannii Isolates from Hospital Environmental and Patients’ Respiratory Tract Sources. Am. J. Infect. Control.

[186] Qasim Z.J., Kadhim H.S., Abdulamir A.S. (2019). Identification of Antibiotic Resistance Genes in Multi-Drug Resistant Acinetobacter Baumannii Clinical Isolates of Iraqi Patients (Zq Strains), Using Whole-Genome Sequencing. Int. J. Pharm. Qual. Assur..

[187] Rabaan A.A., Saunar J.V., Bazzi A.M., Raslan W.F., Taylor D.R., Al-Tawfiq J.A. (2017). Epidemiology and Detection of Acinetobacter Using Conventional Culture and In-House Developed PCR Based Methods. J. Infect. Public Health.

[188] Radhi S.H., Al-Charrakh A.H. (2019). Occurrence of MBLs and Carbapenemases among MDR and XDR *Acinetobacter Baumannii* Isolated from Hospitals in Iraq. Ind. J. Publ. Health Res. Dev..

[189] Ridha D., Ali M., Jassim K. (2019). Occurrence of Metallo-β-Lactamase Genes among Acinetobacter Baumannii Isolated from Different Clinical Samples. J. Pure Appl. Microbiol..

[190] Salahuddin N., Amer L., Joseph M., El Hazmi A., Hawa H., Maghrabi K. (2016). Determinants of Deescalation Failure in Critically Ill Patients with Sepsis: A Prospective Cohort Study. Crit. Care Res. Pract..

[191] Samrah S., Bashtawi Y., Hayajneh W., Almomani B., Momany S., Khader Y. (2016). Impact of Colistin-Initiation Delay on Mortality of Ventilator-Associated Pneumonia Caused by A. Baumannii. J. Infect. Dev. Ctries.

[192] Senok A., Garaween G., Raji A., Khubnani H., Kim Sing G., Shibl A. (2015). Genetic Relatedness of Clinical and Environmental Acinetobacter Baumanii Isolates from an Intensive Care Unit Outbreak. J. Infect. Dev. Ctries.

[193] Shah M.W., Yasir M., Farman M., Jiman-Fatani A.A., Almasaudi S.B., Alawi M., El-Hossary D., Azhar E.I. (2019). Antimicrobial Susceptibility and Molecular Characterization of Clinical Strains of *Acinetobacter Baumannii* in Western Saudi Arabia. Microb. Drug Resist..

[194] Somily A.M. (2010). Comparison of E-Test and Disc Diffusion Methods for the in Vitro Evaluation of the Antimicrobial Activity of Colistin in Multi-Drug Resistant Gram-Negative Bacilli. Saudi Med. J..

[195] Somily A.M., Absar M.M., Arshad M.Z., Al Aska A.I., Shakoor Z.A., Fatani A.J., Siddiqui Y.M., Murray T.S. (2012). Antimicrobial Susceptibility Patterns of Multidrug-Resistant Pseudomonas Aeruginosa and Acinetobacter Baumannii against Carbapenems, Colistin, and Tigecycline. Saudi Med. J..

[196] Vijayakumar R., Sandle T., Al-Aboody M.S., AlFonaisan M.K., Alturaiki W., Mickymaray S., Premanathan M., Alsagaby S.A. (2018). Distribution of Biocide Resistant Genes and Biocides Susceptibility in Multidrug-Resistant Klebsiella Pneumoniae, Pseudomonas Aeruginosa and Acinetobacter Baumannii—A First Report from the Kingdom of Saudi Arabia. J. Infect. Public Health.

[197] Wahaab I.T.A., Almaroof S.Q.M., Yaseen Z.T. (2021). Causative Microorganisms and Antibiotics Susceptibility in Neonatal Sepsis at Neonatal Intensive Care Unit: A Longitudinal Study from Diyala Governorate in Iraq. Indian J. Forensic Med. Toxicol..

[198] Wibberg D., Salto I.P., Eikmeyer F.G., Maus I., Winkler A., Nordmann P., Pühler A., Poirel L., Schlüter A. (2018). Complete Genome Sequencing of Acinetobacter Baumannii Strain K50 Discloses the Large Conjugative Plasmid PK50a Encoding Carbapenemase OXA-23 and Extended-Spectrum β-Lactamase GES-11. Antimicrob. Agents Chemother..

[199] Yasir M., Shah M.W., Jiman-Fatani A.A., Almasaudi S.B., Ahmad H., Alawi M., Azhar E.I. (2019). Draft Genome Sequence of a Clinical Acinetobacter Baumannii Isolate of New Sequence Type ST1688 from Saudi Arabia. J. Glob. Antimicrob. Resist..

[200] Al-Agamy M.H., El-Mahdy T.S., Radwan H.H., Poirel L. (2019). Cooccurrence of NDM-1, ESBL, RmtC, AAC(6′)-Ib, and QnrB in Clonally Related *Klebsiella Pneumoniae* Isolates Together with Coexistence of CMY-4 and AAC(6′)-Ib in *Enterobacter Cloacae* Isolates from Saudi Arabia. BioMed Res. Int..

[201] Alatoom A., Sartawi M., Lawlor K., AbdelWareth L., Thomsen J., Nusair A., Mirza I. (2018). Persistent Candidemia despite Appropriate Fungal Therapy: First Case of Candida Auris from the United Arab Emirates. Int. J. Infect. Dis..

[202] Al-Baloushi A.E., Pál T., Ghazawi A., Sonnevend A. (2018). Genetic Support of Carbapenemases in Double Carbapenemase Producer Klebsiella Pneumoniae Isolated in the Arabian Peninsula. Acta Microbiol. Immunol. Hung..

[203] Alghoribi M.F., Binkhamis K., Alswaji A.A., Alhijji A., Alsharidi A., Balkhy H.H., Doumith M., Somily A. (2020). Genomic Analysis of the First KPC-Producing Klebsiella Pneumoniae Isolated from a Patient in Riyadh: A New Public Health Concern in Saudi Arabia. J. Infect. Public Health.

[204] Algowaihi R., Ashgar S., Sirag B., Shalam S., Nassir A., Ahmed A. (2016). Draft Genome Sequence of a Multidrug-Resistant Klebsiella Pneumoniae Strain Isolated from King Abdullah Medical City, Makkah, Saudi Arabia. Genome Announc..

[205] AL-Khikani F.H.O., Abadi R.M., Ayit A.S. (2020). Emerging Carbapenemase Klebsiella Oxytoca with Multidrug Resistance Implicated in Urinary Tract Infection. Biomed. Biotechnol. Res. J..

[206] AL-Muqdadi B.M.J., AL-Saadi B.Q.H. (2020). Detection Of Arma Gene, Kpc Enzyme And Molecular Typing Of K. Pneumoniae Clinical Isolate From Public Hospitals In Baghdad City, Iraq. Biochem. Cell. Arch..

[207] Al-Qahtani A.A., Al-Agamy M.H., Ali M.S., Al-Ahdal M.N., Aljohi M.A., Shibl A.M. (2014). Characterization of Extended-Spectrum Beta-Lactamase-Producing *Klebsiella Pneumoniae* from Riyadh, Saudi Arabia. J. Chemother..

[208] Alsanie W.F. (2020). Molecular Diversity and Profile Analysis of Virulence-Associated Genes in Some Klebsiella Pneumoniae Isolates. Pract. Lab. Med..

[209] Alyousef A.A., Khazaal S.S., Ali A.N.M., Hussein N.H., Hussein S.M.A. (2017). Detection of Prevalent Mechanism of Extended Spectrum β-Lactamases, Metallo β-Lactamases, and AmpC β Lactamases-Producing Klebsiella Pneumoniae in the Tertiary Care Hospital. Rev. Med. Microbiol..

[210] Salman R.A., Ghaima K.K. (2018). Prevalence of ESBL genes in ESBL producing Klebsiella pneumoniae isolated from patients with urinary tract infections in Baghdad, Iraq. Biosci. Res..

[211] El Nekidy W.S., Mooty M.Y., Attallah N., Cardona L., Bonilla M.F., Ghazi I.M. (2017). Successful Treatment of Multidrug Resistant Klebsiella Pneumoniae Using Dual Carbapenem Regimen in Immunocompromised Patient. IDCases.

[212] Hassan M.I., Alkharsah K.R., Alzahrani A.J., Obeid O.E., Khamis A.H., Diab A. (2013). Detection of Extended Spectrum Beta-Lactamases-Producing Isolates and Effect of AmpC Overlapping. J. Infect. Dev. Ctries.

[213] Lagha R., Ben Abdallah F., ALKhammash A.A.H., Amor N., Hassan M.M., Mabrouk I., Alhomrani M., Gaber A. (2021). Molecular Characterization of Multidrug Resistant Klebsiella Pneumoniae Clinical Isolates Recovered from King Abdulaziz Specialist Hospital at Taif City, Saudi Arabia. J. Infect. Public Health.

[214] Poirel L., Al Maskari Z., Al Rashdi F., Bernabeu S., Nordmann P. (2011). NDM-1-Producing Klebsiella Pneumoniae Isolated in the Sultanate of Oman. J. Antimicrob. Chemother..

[215] Saaid Tuwaij N.S., Al-khilkhali H.J.B., Mohsen H.M. (2020). Prevalence of SUL(1,2), GYR(A, B) and OXA Genes among Multidrug Resistance Klebsiella Pneumoniae Isolates Recovered from Women Suffering Urinary Tract Infection. Int. J. Res. Pharm. Sci..

[216] Shibl A., Al-Agamy M., Memish Z., Senok A., Khader S.A., Assiri A. (2013). The Emergence of OXA-48- and NDM-1-Positive Klebsiella Pneumoniae in Riyadh, Saudi Arabia. Int. J. Infect. Dis..

[217] Shlash A.A.A., Tuwaij N.S.S. (2018). Molecular Dissemination Of Ambler Class A And C Β- Lactamase Genes Among Ceftriaxone Resistant Klebsiella Pneumoniae Infection In Najaf City, Iraq. Biochem. Cell. Arch..

[218] Vali L., Dashti A.A., Jadaon M.M., El-Shazly S. (2015). The Emergence of Plasmid Mediated Quinolone Resistance QnrA2 in Extended Spectrum β-Lactamase Producing Klebsiella Pneumoniae in the Middle East. DARU J. Pharm Sci..

[219] uz Zaman T., Alrodayyan M., Albladi M., Aldrees M., Siddique M.I., Aljohani S., Balkhy H.H. (2018). Clonal Diversity and Genetic Profiling of Antibiotic Resistance among Multidrug/Carbapenem-Resistant Klebsiella Pneumoniae Isolates from a Tertiary Care Hospital in Saudi Arabia. BMC Infect. Dis..

[220] uz Zaman T., Aldrees M., Al Johani S.M., Alrodayyan M., Aldughashem F.A., Balkhy H.H. (2014). Multi-Drug Carbapenem-Resistant Klebsiella Pneumoniae Infection Carrying the OXA-48 Gene and Showing Variations in Outer Membrane Protein 36 Causing an Outbreak in a Tertiary Care Hospital in Riyadh, Saudi Arabia. Int. J. Infect. Dis..

[221] AbdulWahab A., Taj-Aldeen S.J., Ibrahim E.B., Hussain S., Muhammed R., Ahmed I., Abdeen Y., Sadek O., Abu-Madi M. (2014). Genetic Relatedness and Host Specificity of Pseudomonas Aeruginosa Isolates from Cystic Fibrosis and Non-Cystic Fibrosis Patients. Infect. Drug Resist..

[222] AbdulWahab A., Zahraldin K., Ahmed M.A., Jarir S.A., Muneer M., Mohamed S.F., Hamid J.M., Hassan A.A., Ibrahim E.B. (2017). The Emergence of Multidrug-Resistant Pseudomonas Aeruginosa in Cystic Fibrosis Patients on Inhaled Antibiotics. Lung India.

[223] Ahmad I., Irfan S., Abohashrh M., Wahab S., Abullais S.S., Javali M.A., Nisar N., Alam M.M., Srivastava S., Saleem M. (2021). Inhibitory Effect of Nepeta Deflersiana on Climax Bacterial Community Isolated from the Oral Plaque of Patients with Periodontal Disease. Molecules.

[224] Alamri A.M., Alfifi S., Aljehani Y., Alnimr A. (2020). Whole Genome Sequencing of Ceftolozane-Tazobactam and Ceftazidime-Avibactam Resistant Pseudomonas Aeruginosa Isolated from a Blood Stream Infection Reveals VEB and Chromosomal Metallo-Beta Lactamases as Genetic Determinants: A Case Report. Infect. Drug Resist..

[225] Al-Delaimi M.S., Yacoob Aldosky H.Y. (2020). Amending the Efficiency of Antimicrobials against Multidrug-Resistant Pseudomonas Aeruginosa by Low-Frequency Magnetic Fields. Bull. Exp. Biol. Med..

[226] Alhamdani R.J.M., Al-Luaibi Y.Y.Y. (2020). Detection of exoA, nan1 genes, the biofilm production with the effect of Oyster shell and two plant extracts on Pseudomonas aeroginosa isolated from burn’ patient and their surrounding environment. Syst. Rev. Pharm..

[227] Al-Zahrani I.A., Al-Ahmadi B.M. (2021). Dissemination of VIM-Producing *Pseudomonas Aeruginosa* Associated with High-Risk Clone ST654 in a Tertiary and Quaternary Hospital in Makkah, Saudi Arabia. J. Chemother..

[228] Asghar A. (2012). Antimicrobial Susceptibility and Metallo-β-Lactamase Production among Pseudomonas Aeruginosa Isolated from Makkah Hospitals. Pak. J. Med. Sci..

[229] Behbahani M.R., Keshavarzi A., Pirbonyeh N., Javanmardi F., Khoob F., Emami A. (2019). Plasmid- related β-Lactamase Genes in Pseudomonas Aeruginosa Isolates: A Molecular Study in Burn Patients. J. Med. Microbiol..

[230] Bourghli A., Boissiere L., Obeid I. (2019). Thoracic Kyphotic Deformity Secondary to Old *Pseudomonas Aeruginosa* Spondylodiscitis in an Immunocompromised Patient With Persistent Infection Foci—A Case Report. Int. J. Spine Surg..

[231] Hassan S., Najati A., Abass K. (2019). Isolation and Identification of Multi-Drug Resistant “Pseudomonas Aeruginosa” from Burn Wound Infection in Kirkuk City, Iraq. Eurasian J. Biosci..

[232] Hassan S.A., Shobrak M.Y. (2014). Prevalence and Antimicrobial Resistance Characteristics of Gram-Negative Bacteria Associated with Wild Animals Presenting at Live Animal Market, Taif, Western Saudi Arabia. Antimicrob. Resist..

[233] Jaaffar A.I. (2019). Detection of BlaIMP Gene among Pseudomonas Aeruginosa Isolated from Different Clinical Samples. Drug Invent. Today.

[234] Khan R., Al-Dorzi H.M., Tamim H.M., Rishu A.H., Balkhy H., El-Saed A., Arabi Y.M. (2016). The Impact of Onset Time on the Isolated Pathogens and Outcomes in Ventilator Associated Pneumonia. J. Infect. Public Health.

[235] Khan M.A., Faiz A. (2016). Antimicrobial Resistance Patterns of *Pseudomonas Aeruginosa* in Tertiary Care Hospitals of Makkah and Jeddah. Ann. Saudi Med..

[236] Mahdi L.H., Jabbar H.S., Auda I.G. (2019). Antibacterial Immunomodulatory and Antibiofilm Triple Effect of Salivaricin LHM against Pseudomonas Aeruginosa Urinary Tract Infection Model. Int. J. Biol. Macromol..

[237] Nasser M., Gayen S., Kharat A.S. (2020). Prevalence of β-Lactamase and Antibiotic-Resistant Pseudomonas Aeruginosa in the Arab Region. J. Glob. Antimicrob. Resist..

[238] Nasser M., Kharat A.S. (2019). Phenotypic Demonstration of SS-Lactamase (ESßLs, MßLs, and Amp-C) among MDR Pseudomonas Aeruginosa Isolates Obtained From Burn Wound Infected in Yemen. J. Appl. Biol. Biotechnol..

[239] Sid Ahmed M.A., Khan F.A., Sultan A.A., Söderquist B., Ibrahim E.B., Jass J., Omrani A.S. (2020). β-Lactamase-Mediated Resistance in MDR-Pseudomonas Aeruginosa from Qatar. Antimicrob. Resist. Infect. Control.

[240] Tarazi Y.H., Abu-Basha E., Ismail Z.B., Al-Jawasreh S.I. (2021). Antimicrobial Susceptibility of Multidrug-Resistant Pseudomonas Aeruginosa Isolated from Drinking Water and Hospitalized Patients in Jordan. Acta Trop..

[241] Zikri A., El Masri K. (2019). Use of Ceftolozane/Tazobactam for the Treatment of Multidrug-Resistant Pseudomonas Aeruginosa Pneumonia in a Pediatric Patient with Combined Immunodeficiency (CID): A Case Report from a Tertiary Hospital in Saudi Arabia. Antibiotics.

[242] Abulreesh H.H., Organji S.R., Osman G.E.H., Elbanna K., Almalki M.H.K., Ahmad I. (2017). Prevalence of Antibiotic Resistance and Virulence Factors Encoding Genes in Clinical Staphylococcus Aureus Isolates in Saudi Arabia. Clin. Epidemiol. Glob. Health.

[243] Abulreesh H.H., Organji S.R. (2011). The Prevalence of Multidrug-Resistant Staphylococci in Food and the Environment of Makkah, Saudi Arabia. Res. J. Microbiol..

[244] Al Zebary M.K., Yousif S.Y., Assafi M.S. (2017). The Prevalence, Molecular Characterization and Antimicrobial Susceptibility of S. Aureus Isolated from Impetigo Cases in Duhok, Iraq. Open Dermatol. J..

[245] AlFouzan W., Al-Haddad A., Udo E., Mathew B., Dhar R. (2013). Frequency and Clinical Association of Panton-Valentine Leukocidin-Positive *Staphylococcus Aureus* Isolates: A Study from Kuwait. Med Princ. Pract..

[246] Alghizzi M.J., Alansari M., Shami A. (2021). The Prevalence of Staphylococcus Aureus and Methicillin Resistant Staphylococcus Aureus in Processed Food Samples in Riyadh, Saudi Arabia. J. Pure Appl. Microbiol..

[247] Al-Zoubi M.S., Al-Tayyar I.A., Hussein E., Jabali A.A., Khudairat S. (2015). Antimicrobial Susceptibility Pattern of Staphylococcus Aureus Isolated from Clinical Specimens in Northern Area of Jordan. Iran. J. Microbiol..

[248] Hamid M.E. (2011). Prevalence of Bacterial Pathogens in Aseer Region, Kingdom of Saudi Arabia: Emphasis on Antimicrobial Susceptibility of Staphylococcus Aureus. Oman Med. J..

[249] Ismail Z.B. (2017). Molecular Characteristics, Antibiogram and Prevalence of Multi-Drug Resistant Staphylococcus Aureus (MDRSA) Isolated from Milk Obtained from Culled Dairy Cows and from Cows with Acute Clinical Mastitis. Asian Pac. J. Trop. Biomed..

[250] Kanaan M.H.G. (2018). Antibacterial Effect of Ozonated Water against Methicillin-Resistant Staphylococcus Aureus Contaminating Chicken Meat in Wasit Province, Iraq. Vet. World.

[251] Kanaan M.H.G., Al-Isawi A.J.O. (2019). Prevalence Of Methicillin Or Multiple Drug-Resistant Staphylococcus Aureus In Cattle Meat Marketed In Wasit Province. Biochem. Cell. Arch..

[252] Obaidat M.M., Bani Salman A.E., Lafi S.Q. (2015). Prevalence of Staphylococcus Aureus in Imported Fish and Correlations between Antibiotic Resistance and Enterotoxigenicity. J. Food Prot..

[253] Vellappally S., Divakar D.D., Al Kheraif A.A., Ramakrishnaiah R., Alqahtani A., Dalati M.H.N., Anil S., Khan A.A., Harikrishna Varma P.R. (2017). Occurrence of Vancomycin-Resistant Staphylococcus Aureus in the Oral Cavity of Patients with Dental Caries. Acta Microbiol. Et Immunol. Hung..

[254] Abdalla N.M., Osman A.A., Haimour W.O., Sarhan M.A., Mohammed M.N., Zyad E.M., Al-ghtani A.M. (2013). Antimicrobial Susceptibility Pattern in Nosocomial Infections Caused by Acinetobacter Species in Asir Region, Saudi Arabia. Pak. J. Biol. Sci..

[255] Balkhy H.H., Bawazeer M.S., Kattan R.F., Tamim H.M., Johani S.M., Aldughashem F.A., Al Alem H.A., Adlan A., Herwaldt L.A. (2012). Epidemiology of Acinetobacter spp.-Associated Healthcare Infections and Colonization among Children at a Tertiary-Care Hospital in Saud Arabia: A 6-Year Retrospective Cohort Study. Eur. J. Clin. Microbiol. Infect. Dis..

[256] Somily A.M., Al-Khattaf A.S., Kambal A.M. (2010). Antimicrobial Activity of Tigecycline against Bacterial Isolates from Intensive Care Units in a Teaching Hospital in Central Saudi Arabia. Saudi Med. J..

[257] Al-Kharousi Z.S., Guizani N., Al-Sadi A.M., Al-Bulushi I.M. (2019). Antibiotic Resistance of Enterobacteriaceae Isolated from Fresh Fruits and Vegetables and Characterization of Their AmpC β-Lactamases. J. Food Prot..

[258] Alkofide H., Alhammad A.M., Alruwaili A., Aldemerdash A., Almangour T.A., Alsuwayegh A., Almoqbel D., Albati A., Alsaud A., Enani M. (2020). Multidrug-Resistant and Extensively Drug-Resistant Enterobacteriaceae: Prevalence, Treatments, and Outcomes—A Retrospective Cohort Study. Infect. Drug Resist..

[259] Jamal W.Y., Albert M.J., Rotimi V.O. (2016). High Prevalence of New Delhi Metallo-β-Lactamase-1 (NDM-1) Producers among Carbapenem-Resistant Enterobacteriaceae in Kuwait. PLoS ONE.

[260] Al-Mayahi F.S.A., Ali R.H. (2018). Preliminary study of emergence MDR of Providencia spp. isolates producing ESBL, AmpC and MBL among patients with RTI and in wastewater in Al-Diwaniya city, Iraq. Biochem. Cell. Arch..

[261] Ayyal Al-Gburi N.M. (2020). Isolation and Molecular Identification and Antimicrobial Susceptibility of Providencia spp. from Raw Cow’s Milk in Baghdad, Iraq. Vet. Med. Int..

[262] Al-Lahham A., Qayyas J.A. (2018). The Impact of the 7-Valent Pneumococcal Conjugate Vaccine on Nasopharyngeal Carriage of Streptococcus Pneumoniae in Infants of Ajlun Governorate in Jordan. Jordan J. Biol. Sci..

[263] Al-Mazrou K.A., Shibl A.M., Kandeil W., Pirçon J.-Y., Marano C. (2014). A Prospective, Observational, Epidemiological Evaluation of the Aetiology and Antimicrobial Susceptibility of Acute Otitis Media in Saudi Children Younger than 5 Years of Age. J. Epidemiol. Glob. Health.

[264] Almazrou Y., Shibl A.M., Alkhlaif R., Pirçon J.-Y., Anis S., Kandeil W., Hausdorff W.P. (2015). Epidemiology of Invasive Pneumococcal Disease in Saudi Arabian Children Younger than 5 Years of Age. J. Epidemiol. Glob. Health.

[265] Alnimr A.M., Farhat M. (2017). Phenotypic and Molecular Study of Pneumococci Causing Respiratory Tract Infections: A 3-Year Prospective Cohort. Saudi Med. J..

[266] Al-Sa’ady A.T., Hussein F.H. (2020). Nanomedical Applications of Titanium Dioxide Nanoparticles as Antibacterial Agent against Multi-Drug Resistant Streptococcus Pneumoniae. Syst. Rev. Pharm..

[267] Elshafie S., Taj-Aldeen S.J. (2016). Emerging Resistant Serotypes of Invasive Streptococcus Pneumoniae. Infect. Drug Resist..

[268] Sallam M. (2019). Trends in Antimicrobial Drug Resistance of Streptococcus Pneumoniae Isolates at Jordan University Hospital (2000–2018). Antibiotics.

[269] Shin J., Baek J.Y., Kim S.H., Song J.-H., Ko K.S. (2011). Predominance of ST320 among Streptococcus Pneumoniae Serotype 19A Isolates from 10 Asian Countries. J. Antimicrob. Chemother..

[270] Al Baidani A., Elshoni W., Shawa T. (2011). Antibiotic Susceptibility Pattern of Methicillin-Resistant Staphylococcus Aureus in Three Hospitals at Hodeidah City, Yemen. Glob. J. Pharmacol..

[271] Albarrag A., Shami A., Almutairi A., Alsudairi S., Aldakeel S., Al-Amodi A. (2020). Prevalence and Molecular Genetics of Methicillin-Resistant *Staphylococcus Aureus* Colonization in Nursing Homes in Saudi Arabia. Can. J. Infect. Dis. Med. Microbiol..

[272] Alfouzan W., Udo E.E., Modhaffer A., Alosaimi A. (2019). Molecular Characterization of Methicillin- Resistant Staphylococcus Aureus in a Tertiary Care Hospital in Kuwait. Sci. Rep..

[273] Alhussaini M.S. (2016). Methicillin-Resistant Staphylococcus Aureus Nasal Carriage Among Patients Admitted at Shaqra General Hospital in Saudi Arabia. Pak. J. Biol. Sci..

[274] Alkharsah K.R., Rehman S., Alkhamis F., Alnimr A., Diab A., Al-Ali A.K. (2018). Comparative and Molecular Analysis of MRSA Isolates from Infection Sites and Carrier Colonization Sites. Ann. Clin. Microbiol. Antimicrob..

[275] Aziz Z.S., Hassan M.A. (2019). Phenotypic and Molecular Study of *MecA* Gene in MRSA Isolated from Clinical Cases in Misan Province/Iraq. Indian J. Public Health.

[276] Helmi N.R., Zaman R.M., Aly M.M. (2013). Prevalence Of Gram-Positive Bacteria In Jeddah, Kingdom Of Saudi Arabia: Study Of Antimicrobial Resistance Patterns And Molecular Typing. Int. J. Pharma Biol. Sci..

[277] Obaidat M.M., Bani Salman A.E., Roess A.A. (2018). High Prevalence and Antimicrobial Resistance of MecA Staphylococcus Aureus in Dairy Cattle, Sheep, and Goat Bulk Tank Milk in Jordan. Trop. Anim. Health Prod..

[278] Udo E.E., Boswihi S.S. (2017). Antibiotic Resistance Trends in Methicillin-Resistant *Staphylococcus aureus* Isolated in Kuwait Hospitals: 2011–2015. Med Princ. Pract..

[279] Hala S., Antony C.P., Alshehri M., Althaqafi A.O., Alsaedi A., Mufti A., Kaaki M., Alhaj-Hussein B.T., Zowawi H.M., Al-Amri A. (2019). First Report of Klebsiella Quasipneumoniae Harboring BlaKPC-2 in Saudi Arabia. Antimicrob. Resist. Infect. Control.

[280] Ejaz H., Alzahrani B., Hamad M.F.S., Abosalif K.O.A., Junaid K., Abdalla A.E., Elamir M.Y.M., Aljaber N.J., Hamam S.S.M., Younas S. (2020). Molecular Analysis of the Antibiotic Resistant NDM-1 Gene in Clinical Isolates of Enterobacteriaceae. Clin. Lab..

[281] Ahmad S., Khan Z., Al-Sweih N., Alfouzan W., Joseph L. (2020). *Candida Auris* in Various Hospitals across Kuwait and Their Susceptibility and Molecular Basis of Resistance to Antifungal Drugs. Mycoses.

[282] Emara M., Ahmad S., Khan Z., Joseph L., Al-Obaid I., Purohit P., Bafna R. (2015). *Candida Auris* Candidemia in Kuwait, 2014. Emerg. Infect. Dis..

[283] Khan Z., Ahmad S., Benwan K., Purohit P., Al-Obaid I., Bafna R., Emara M., Mokaddas E., Abdullah A.A., Al-Obaid K. (2018). Invasive Candida Auris Infections in Kuwait Hospitals: Epidemiology, Antifungal Treatment and Outcome. Infection.

[284] Hassan M.M., Belal E.-S.B. (2016). Antibiotic Resistance and Virulence Genes in Enterococcus Strains Isolated from Different Hospitals in Saudi Arabia. Biotechnol. Biotechnol. Equip..

[285] Salem-Bekhit M., Moussa I., Muharram M., Alanazy F., Hefni H. (2012). Prevalence and Antimicrobial Resistance Pattern of Multidrug-Resistant Enterococci Isolated from Clinical Specimens. Indian J. Med. Microbiol..

[286] Dibby H., Shlash R. (2020). The Problem of Multidrug Resistance Bacterial Strains in Daily Clinical Practice in Dealing with Typhoid Fever in Mid-Euphrates Region of Iraq: A Cross Sectional Study. Indian J. Forensic Med. Toxicol..

[287] El-Tayeb M.A., Ibrahim A.S.S., Al-Salamah A.A., Almaary K.S., Elbadawi Y.B. (2017). Prevalence, Serotyping and Antimicrobials Resistance Mechanism of Salmonella Enterica Isolated from Clinical and Environmental Samples in Saudi Arabia. Braz. J. Microbiol..

[288] Harb A., O’Dea M., Hanan Z.K., Abraham S., Habib I. (2017). Prevalence, Risk Factors and Antimicrobial Resistance of *Salmonella* Diarrhoeal Infection among Children in Thi-Qar Governorate, Iraq. Epidemiol. Infect..

[289] Sahan Z.A., Hamed S.L. (2020). Molecular Detection of Extended-Spectrum β-Lactamases- Producer Serratia Marcescens Causing Neonatal Sepsis in Iraq. Int. J. Res. Pharm. Sci..

[290] Abdel-Haleem A.M., Rchiad Z., Khan B.K., Abdallah A.M., Naeem R., Nikhat Sheerin S., Solovyev V., Ahmed A., Pain A. (2015). Genome Sequence of a Multidrug-Resistant Strain of Stenotrophomonas Maltophilia with Carbapenem Resistance, Isolated from King Abdullah Medical City, Makkah, Saudi Arabia. Genome Announc..

[291] El-Kersh T.A., Marie M.A., Al-Sheikh Y.A., Al-Agamy M.H., Al-Bloushy A.A. (2016). Prevalence and Risk Factors of Early Fecal Carriage of *Enterococcus Faecalis* and *Staphylococcus* spp. and Their Antimicrobial Resistant Patterns among Healthy Neonates Born in a Hospital Setting in Central Saudi Arabia. Saudi Med. J..

[292] Farman M., Yasir M., Al-Hindi R.R., Farraj S.A., Jiman-Fatani A.A., Alawi M., Azhar E.I. (2019). Genomic Analysis of Multidrug-Resistant Clinical Enterococcus Faecalis Isolates for Antimicrobial Resistance Genes and Virulence Factors from the Western Region of Saudi Arabia. Antimicrob. Resist. Infect. Control.

[293] George S.K., Suseela M.R., El Safi S., Elnagi E.A., Al-Naam Y.A., Adam A.A., Jacob A.M., Al-Maqati T., Ks H.K. (2021). Molecular Determination of van Genes among Clinical Isolates of Enterococci at a Hospital Setting. Saudi J. Biol. Sci..

[294] Garaween G., Somily A., Raji A., Braun S., Al-Kattan W., Shibl A., Ehricht R., Senok A. (2016). Serogenotyping and Emergence of Extended-Spectrum β-Lactamase Genes in Non-Typhoidal Salmonella: First Report from Saudi Arabia. J. Med. Microbiol..

[295] Alsalem Z., Elhadi N., Aljeldah M., Alzahrani F., Nishibuchi M. (2018). Characterization of Vibrio Vulnificus Isolated from the Coastal Areas in the Eastern Province of Saudi Arabia. J. Pure Appl. Microbiol..

[296] M Kurdi Al-Dulaimi M., Abd Mutalib S., Abd Ghani M., Mohd Zaini N.A., Ariffin A.A. (2019). Multiple Antibiotic Resistance (MAR), Plasmid Profiles, and DNA Polymorphisms among Vibrio Vulnificus Isolates. Antibiotics.

[297] Abro A.H. (2011). Salmonella Typhi: Antibiotic Sensitivity Pattern in Dubai, United Arab Emirates. Pak. J. Med. Sci..

[298] AL-Fatlawy H.N.K., AL-Hadrawi H.A.N. (2020). Molecular Profiling of Class I Integron Gene in MDR Salmonella Typhi Isolates. J. Pure Appl. Microbiol..

[299] Yassin M.T., Mostafa A.A., Al-Askar A.A., Bdeer R. (2020). In Vitro Antifungal Resistance Profile of Candida Strains Isolated from Saudi Women Suffering from Vulvovaginitis. Eur. J. Med. Res..

[300] Alaboudi A.R., Malkawi I.M., Osaili T.M., Abu-Basha E.A., Guitian J. (2020). Prevalence, Antibiotic Resistance and Genotypes of Campylobacter Jejuni and Campylobacter Coli Isolated from Chickens in Irbid Governorate, Jordan. Int. J. Food Microbiol..

[301] Ghunaim H., Behnke J.M., Aigha I., Sharma A., Doiphode S.H., Deshmukh A., Abu-Madi M.M. (2015). Analysis of Resistance to Antimicrobials and Presence of Virulence/Stress Response Genes in Campylobacter Isolates from Patients with Severe Diarrhoea. PLoS ONE.

[302] Kanaan M.H.G., Mohammed F.A. (2020). Antimicrobial Resistance of Campylobacter Jejuni from Poultry Meat in Local Markets of Iraq. Plant Arch..

[303] Jasim S.A., Al-abodi H.R., Ali W.S. (2021). Resistance Rate and Novel Virulence Factor Determinants of Arcobacter spp., from Cattle Fresh Meat Products from Iraq. Microb. Pathog..

[304] AbdAlhussen L.S., Darweesh M.F. (2016). Prevelance and Antibiotic Susceptibility Patterns of Pantoea spp.. Int. J. ChemTech Res..

[305] Fazaa S.A., Darweesh M.F. (2020). Prevalence of Pantoea spp. among Recurrent UTI Patients with Emphasis on Risk Factors and Antibiotic Resistance Patterns in Al-Qadisiyah Hospitals, Iraq. Ann. Trop. Med. Public Health.

[306] Burjaq S.Z., Abu-Romman S.M. (2020). Prevalence and Antimicrobial Resistance of Salmonella spp. From Irrigation Water in Two Major Sources in Jordan. Curr. Microbiol..

[307] Jassim A.A., Al-Gburi N.M. (2020). Virulence Genes And Antimicrobial Resistance Of Salmonella Isolated From Milk In Wasit Province, Iraq. Plant Arch..

[308] Olaitan A.O., Dia N.M., Gautret P., Benkouiten S., Belhouchat K., Drali T., Parola P., Brouqui P., Memish Z., Raoult D. (2015). Acquisition of Extended-Spectrum Cephalosporin- and Colistin-Resistant Salmonella Enterica Subsp. Enterica Serotype Newport by Pilgrims during Hajj. Int. J. Antimicrob. Agents.

[309] Osaili T.M., Al-Nabulsi A.A., Shaker R.R., Jaradat Z.W., Taha M., Al-Kherasha M., Meherat M., Holley R. (2014). Prevalence of Salmonella Serovars, Listeria Monocytogenes, and Escherichia coli O157:H7 in Mediterranean Ready-to-Eat Meat Products in Jordan. J. Food Prot..

[310] Abd Al-Mayahi F.S., Jaber S.M. (2020). Multiple Drug Resistance of Listeria Monocytogenes Isolated from Aborted Women by Using Serological and Molecular Techniques in Diwaniyah City/Iraq. Iran. J. Microbiol..

[311] Ahmed M.S., Taha Z.M.A., Omer L.T. (2015). Isolation and Molecular Identification with Resistant Profile Determination of Listeria Monocytogenes from Imported Chicken Carcasses in Duhok, Kurdistan Region, Iraq. J. Pure Appl. Microbiol..

[312] Alanber M.N., Alharbi N.S., Khaled J.M. (2020). Evaluation of Multidrug-Resistant Bacillus Strains Causing Public Health Risks in Powdered Infant Milk Formulas. J. Infect. Public Health.

[313] Hayder T., Aljanaby A.A.J.J. (2019). Antibiotics Susceptibility Patterns of Citrobacter Freundii Isolated from Pa-Tients with Urinary Tract Infection in Al-Najaf Governorate—Iraq. Int. J. Res. Pharm. Sci..

[314] Talat A., Khalid S., Majeed H.A.R., Khan A.U. (2020). Whole-Genome Sequence Analysis of Multidrug-Resistant Staphylococcus EpidermiDis. ST35 Strain Isolated from Human Ear Infection of an Iraqi Patient. J. Glob. Antimicrob. Resist..

[315] Al-Muhanna A.S., Al-Muhanna S., Alzuhairi M.A. (2016). Molecular Investigation of Extended-Spectrum Beta-Lactamase Genes and Potential Drug Resistance in Clinical Isolates of *Morganella Morganii*. Ann. Saudi Med..

[316] Hanan Z.K. (2019). Molecular Detection of Cholera Infection during the Outbreak in Thi-Qar Province/Iraq in 2015–2016. J. Phys. Conf. Ser..

[317] AlJindan R., AlEraky D.M., Borgio J.F., AbdulAzeez S., Abdalhamid B., Mahmoud N., Farhat M. (2021). Diagnostic Deficiencies of C. Difficile Infection among Patients in a Tertiary Hospital in Saudi Arabia: A Laboratory-Based Case Series. Saudi J. Biol. Sci..

[318] Al-Sa’ady A.T. (2019). Detection of Vancomycin Resistance in Multidrug-Resistant Enterococcus Faecalis Isolated from Burn Infections. Drug Invent. Today.

[319] Ulger Toprak N., Veloo A.C.M., Urban E., Wybo I., Justesen U.S., Jean-Pierre H., Morris T., Akgul O., Kulekci G., Soyletir G. (2018). A Multicenter Survey of Antimicrobial Susceptibility of Prevotella Species as Determined by Etest Methodology. Anaerobe.

[320] Taj-Aldeen S.J., Deshmukh A., Doiphode S., Abdul Wahab A., Allangawi M., AlMuzrkchi A., Klaassen C.H., Meis J.F. (2013). Molecular Identification and Susceptibility Pattern of Clinical *Nocardia* Species: Emergence of *Nocardia Crassostreae* as an Agent of Invasive Nocardiosis. Can. J. Infect. Dis. Med. Microbiol..

[321] El-Mahdy T.S., Al-Agamy M.H., Al-Qahtani A.A., Shibl A.M. (2017). Detection of *Bla*
_OXA-23-like_ and *Bla*
_NDM-1_ in *Acinetobacter Baumannii* from the Eastern Region, Saudi Arabia. Microb. Drug Resist..

[322] Khan Z., Ahmad S., Joseph L., Al-Obaid K. (2014). Isolation of Cholesterol-Dependent, Multidrug-Resistant Candida Glabratastrains from Blood Cultures of a Candidemia Patient in Kuwait. BMC Infect. Dis..

[323] Alosaimi R.S., Kaaki M.M. (2020). Catheter-Related ESBL-Producing *Leclercia Adecarboxylata* Septicemia in Hemodialysis Patient: An Emerging Pathogen?. Case Rep. Infect. Dis..

[324] Guan Q., Almutairi T.S., Alhalouli T., Pain A., Alasmari F. (2020). Metagenomics of Imported Multidrug-Resistant *Mycobacterium Leprae*, Saudi Arabia, 2017. Emerg. Infect. Dis..

[325] Abdalhamid B., Elhadi N., Alsamman K., Aljindan R. (2016). Chryseobacterium Gleum Pneumonia in an Infant with Nephrotic Syndrome. IDCases.

[326] Aljindan R., Elhadi N. (2018). Genetic Relationship of Multi-Resistant Acinetobacter Baumannii Isolates in Kingdom of Saudi Arabia. J. Pure Appl. Microbiol..

[327] Yasir M., Farman M., Shah M.W., Jiman-Fatani A.A., Othman N.A., Almasaudi S.B., Alawi M., Shakil S., Al-Abdullah N., Ismaeel N.A. (2020). Genomic and Antimicrobial Resistance Genes Diversity in Multidrug-Resistant CTX-M-Positive Isolates of Escherichia coli at a Health Care Facility in Jeddah. J. Infect. Public Health.

[328] Johani K., Abualsaud D., Costa D.M., Hu H., Whiteley G., Deva A., Vickery K. (2018). Characterization of Microbial Community Composition, Antimicrobial Resistance and Biofilm on Intensive Care Surfaces. J. Infect. Public Health.

[329] Hamza W., Salama M., Morsi S., Abdo N., Al-Fadhli M. (2018). Benchmarking for Surgical Site Infections among Gastrointestinal Surgeries and Related Risk Factors: Multicenter Study in Kuwait. Infect. Drug Resist..

[330] Balkhair A., Al-Farsi Y.M., Al-Muharrmi Z., Al-Rashdi R., Al-Jabri M., Neilson F., Al-Adawi S.S., El-Beeli M., Al-Adawi S. (2014). Epidemiology of Multi-Drug Resistant Organisms in a Teaching Hospital in Oman: A One-Year Hospital-Based Study. Sci. World J..

[331] Al Awaidy S.T. (2018). Tuberculosis Elimination in Oman: Winning the War on the Disease. ERJ Open Res..

[332] Metry A.M., Al Salmi I., Al-Abri S., Al Ismaili F., Al Mahrouqi Y., Hola A., Shaheen F.A.M. (2017). Epidemiology and Outcome of Tuberculosis in Immunocompromised Patients. Saudi J. Kidney Dis. Transpl..

[333] Hasan M.R., Sundaram M.S., Sundararaju S., Tsui K.-M., Karim M.Y., Roscoe D., Imam O., Janahi M.A., Thomas E., Dobson S. (2020). Unusual Accumulation of a Wide Array of Antimicrobial Resistance Mechanisms in a Patient with Cytomegalovirus-Associated Hemophagocytic Lymphohistiocytosis: A Case Report. BMC Infect. Dis..

[334] Alkhawaja E., Hammadi S., Abdelmalek M., Mahasneh N., Alkhawaja B., Abdelmalek S.M. (2020). Antibiotic Resistant Cutibacterium Acnes among Acne Patients in Jordan: A Cross Sectional Study. BMC Dermatol..

[335] Al-Shamahy H.A., Sabrah A.A., Al-Robasi A.B., Naser S.M. (2012). Types of Bacteria Associated with Neonatal Sepsis in Al-Thawra University Hospital, Sana’a, Yemen, and Their Antimicrobial Profile. Sultan Qaboos Univ. Med J..

[336] Kordalewska M., Guerrero K.D., Garcia-Rubio R., Jiménez-Ortigosa C., Mediavilla J.R., Cunningham M.H., Hollis F., Hong T., Chow K.F., Kreiswirth B.N. (2021). Antifungal Drug Susceptibility and Genetic Characterization of Fungi Recovered from COVID-19 Patients. J. Fungi.

[337] Al-Mutairi N.M., Ahmad S., Mokaddas E. (2011). Performance Comparison of Four Methods for Detecting Multidrug-Resistant Mycobacterium Tuberculosis Strains. Int. J. Tuberc. Lung Dis..

[338] Ali R.M., Alsudani A.A. (2016). Discordance between GeneXpert Assay and Conventional Drug-Susceptibility Testing in Detecting Rifampicin-Resistant Tuberculosis: A Perspective of the Line Probe Assay. Int. J. Mycobacteriol..

[339] Arastehfar A., Fang W., Daneshnia F., Al-Hatmi A.M., Liao W., Pan W., Khan Z., Ahmad S., Rosam K., Lackner M. (2019). Novel Multiplex Real-Time Quantitative PCR Detecting System Approach for Direct Detection of *Candida Auris* and Its Relatives in Spiked Serum Samples. Future Microbiol..

[340] Alabdullatif M., Alrehaili J. (2020). Three Years of Evaluation to Determine Reduction of Antibiotic Resistance in Gram-Negative Bacteria by the Saudi National Action Plan. Infect. Drug Resist..

[341] Amoudy H.A., Safar H.A., Mustafa A.S. (2016). Development of Escherichia coli and Mycobacterium Smegmatis Recombinants Expressing Major Mycobacterium Tuberculosis-Specific Antigenic Proteins. Int. J. Mycobacteriol..

[342] Lesho E., Lin X., Clifford R., Snesrud E., Onmus-Leone F., Appalla L., Ong A., Maybank R., Nielsen L., Kwak Y. (2016). From the Battlefield to the Bedside: Supporting Warfighter and Civilian Health With the “ART” of Whole Genome Sequencing for Antibiotic Resistance and Outbreak Investigations. Mil. Med..

[343] Al Tall Y., Abualhaijaa A., Alsaggar M., Almaaytah A., Masadeh M., Alzoubi K.H. (2019). Design and Characterization of a New Hybrid Peptide from LL-37 and BMAP-27. Infect. Drug Resist..

[344] Almaaytah A., Abualhaijaa A., Alqudah O. (2019). The Evaluation of the Synergistic Antimicrobial and Antibiofilm Activity of AamAP1-Lysine with Conventional Antibiotics against Representative Resistant Strains of Both Gram-Positive and Gram-Negative Bacteria. Infect. Drug Resist..

[345] Obeidat M., Shatnawi M., Al-alawi M., Al-Zu‘bi E., Al-Dmoor H., Al-Qudah M., El-Qudah J., Otri I. (2012). Antimicrobial Activity of Crude Extracts of Some Plant Leaves. Res. J. Microbiol..

[346] Ahmed A.A., Salih F.A. (2019). Low Concentrations of Local Honey Modulate ETA Expression, and Quorum Sensing Related Virulence in Drug-Resistant Pseudomonas Aeruginosa Recovered from Infected Burn Wounds. Iran. J. Basic Med. Sci..

[347] Al-Somat M., Al-Adhal A., Al-Arwali A., Al K., Al-Moyed K., Shopit A., Karbane M.E., AL-Kamarany M.A. (2014). Sensitivity of Clinical Bacterial Isolates to Honey Marketed in Yemen. Int. J. Pharm. Sci. Rev. Res..

[348] Darwish R.M., Fares R.J.A., Zarga M.H.A., Nazer I.K. (2010). Antibacterial Effect of Jordanian Propolis and Isolated Flavonoids against Human Pathogenic Bacteria. Afr. J. Biotechnol..

[349] Yang S.-K., Yusoff K., Thomas W., Akseer R., Alhosani M.S., Abushelaibi A., Lim S.-H.-E., Lai K.-S. (2020). Lavender Essential Oil Induces Oxidative Stress Which Modifies the Bacterial Membrane Permeability of Carbapenemase Producing Klebsiella Pneumoniae. Sci. Rep..

[350] Soliman S.S.M., Semreen M.H., El-Keblawy A.A., Abdullah A., Uppuluri P., Ibrahim A.S. (2017). Assessment of Herbal Drugs for Promising Anti-Candida Activity. BMC Complement Altern Med..

[351] Perveen K., Husain F.M., Qais F.A., Khan A., Razak S., Afsar T., Alam P., Almajwal A.M., Abulmeaty M.M.A. (2021). Microwave-Assisted Rapid Green Synthesis of Gold Nanoparticles Using Seed Extract of Trachyspermum Ammi: ROS Mediated Biofilm Inhibition and Anticancer Activity. Biomolecules.

[352] Almaaytah A., Qaoud M., Khalil Mohammed G., Abualhaijaa A., Knappe D., Hoffmann R., Al-Balas Q. (2018). Antimicrobial and Antibiofilm Activity of UP-5, an Ultrashort Antimicrobial Peptide Designed Using Only Arginine and Biphenylalanine. Pharmaceuticals.

[353] Soliman S.S.M., Saeed B.Q., Elseginy S.A., Al-Marzooq F., Ahmady I.M., El-Keblawy A.A., Hamdy R. (2021). Critical Discovery and Synthesis of Novel Antibacterial and Resistance-Modifying Agents Inspired by Plant Phytochemical Defense Mechanisms. Chem.-Biol. Interact..

[354] Muthukrishnan P., Chithra Devi D., Mostafa A.A., Alsamhary K.I., Abdel-Raouf N., Nageh Sholkamy E. (2020). Antimicrobial Efficacy of Nocardiopsis Sp. MK_MSt033 against Selected Multidrug Resistant Clinical Microbial Pathogens. J. Infect. Public Health.

[355] Almaaytah A., Ajingi Y., Abualhaijaa A., Tarazi S., Alshar’i N., Al-Balas Q. (2017). Peptide Consensus Sequence Determination for the Enhancement of the Antimicrobial Activity and Selectivity of Antimicrobial Peptides. Infect. Drug Resist..

[356] Almaaytah A., Mohammed G., Abualhaijaa A., Al-Balas Q. (2017). Development of Novel Ultrashort Antimicrobial Peptide Nanoparticles with Potent Antimicrobial and Antibiofilm Activities against Multidrug-Resistant Bacteria. Drug Des. Dev. Ther..

[357] Mechkarska M., Ahmed E., Coquet L., Leprince J., Jouenne T., Vaudry H., King J.D., Conlon J.M. (2010). Antimicrobial Peptides with Therapeutic Potential from Skin Secretions of the Marsabit Clawed Frog Xenopus Borealis (Pipidae). Comp. Biochem. Physiol. Part C Toxicol. Pharmacol..

[358] Omran Z., Bader A., Porta A., Vandamme T., Anton N., Alehaideb Z., El-Said H., Faidah H., Essa A., Vassallo A. (2020). Evaluation of Antimicrobial Activity of Triphala Constituents and Nanoformulation. Evid.-Based Complementary Altern. Med..

[359] Mahdi L., Musafer H., Zwain L., Salman I., Al-Joofy I., Rasool K., Mussa A., Al-kakei S., Al-Oqaili R., Al-Alak S. (2017). Two Novel Roles of Buffalo Milk Lactoperoxidase, Antibiofilm Agent and Immunomodulator against Multidrug Resistant Salmonella Enterica Serovar Typhi and Listeria Monocytogenes. Microb. Pathog..

[360] Rahmeh R., Akbar A., Kishk M., Al-Onaizi T., Al-Azmi A., Al-Shatti A., Shajan A., Al-Mutairi S., Akbar B. (2019). Distribution and Antimicrobial Activity of Lactic Acid Bacteria from Raw Camel Milk. New Microbes New Infect..

[361] Abdel-Aziz H., Eldehna W., Keeton A., Piazza G., Kadi A., Attwa M., Abdelhameed A., Attia M. (2017). Isatin-Benzoazine Molecular Hybrids as Potential Antiproliferative Agents: Synthesis and in Vitro Pharmacological Profiling. Drug Des. Dev. Ther..

[362] Aboul-Fadl T., Bin-Jubair F.A.S., Aboul-Wafa O. (2010). Schiff Bases of Indoline-2,3-Dione (Isatin) Derivatives and Nalidixic Acid Carbohydrazide, Synthesis, Antitubercular Activity and Pharmacophoric Model Building. Eur. J. Med. Chem..

[363] Ahamed A., Arif I.A., Mateen M., Surendra Kumar R., Idhayadhulla A. (2018). Antimicrobial, Anticoagulant, and Cytotoxic Evaluation of Multidrug Resistance of New 1,4-Dihydropyridine Derivatives. Saudi J. Biol. Sci..

[364] Bogue A.L., Panmanee W., McDaniel C.T., Mortensen J.E., Kamau E., Actis L.A., Johannigman J.A., Schurr M.J., Satish L., Kotagiri N. (2021). AB569, a Non-Toxic Combination of Acidified Nitrite and EDTA, is Effective at Killing the Notorious Iraq/Afghanistan Combat Wound Pathogens, Multi-Drug Resistant Acinetobacter Baumannii and Acinetobacter spp.. PLoS ONE.

[365] Venugopala K.N., Uppar V., Chandrashekharappa S., Abdallah H.H., Pillay M., Deb P.K., Morsy M.A., Aldhubiab B.E., Attimarad M., Nair A.B. (2020). Cytotoxicity and Antimycobacterial Properties of Pyrrolo[1,2-a]Quinoline Derivatives: Molecular Target Identification and Molecular Docking Studies. Antibiotics.

[366] Ahmad I., Wahab S., Nisar N., Dera A.A., Alshahrani M.Y., Abullias S.S., Irfan S., Alam M.M., Srivastava S. (2020). Evaluation of Antibacterial Properties of Matricaria Aurea on Clinical Isolates of Periodontitis Patients with Special Reference to Red Complex Bacteria. Saudi Pharm. J..

[367] Al-Asmari A.K., Alamri M.A., Almasoudi A.S., Abbasmanthiri R., Mahfoud M. (2017). Evaluation of the in Vitro Antimicrobial Activity of Selected Saudi Scorpion Venoms Tested against Multidrug-Resistant Micro-Organisms. J. Glob. Antimicrob. Resist..

[368] Alkhedaide A.Q., Ismail T.A., Alotaibi S.H., Nassan M.A., Shehri Z.S.A. (2018). Preventive Effect of Artemisinin Extract against Cholestasis Induced via Lithocholic Acid Exposure. Biosci. Rep..

[369] El-Newehy M.H., Moydeen A. M., Aldalbahi A.K., Thamer B.M., Mahmoud Y.A.-G., El-Hamshary H. (2020). Biocidal Polymers: Synthesis, Characterization and Antimicrobial Activity of Bis-Quaternary Onium Salts of Poly(Aspartate-Co-Succinimide). Polymers.

[370] Manzoor S., Bilal A., Khan S., Ullah R., Iftikhar S., Emwas A.-H., Alazmi M., Gao X., Jawaid A., Saleem R.S.Z. (2018). Identification and Characterization of SSE15206, a Microtubule Depolymerizing Agent That Overcomes Multidrug Resistance. Sci. Rep..

[371] Al-Dhabi N.A., Ghilan A.-K.M., Esmail G.A., Valan Arasu M., Duraipandiyan V., Ponmurugan K. (2019). Bioactivity Assessment of the Saudi Arabian Marine Streptomyces Sp. Al-Dhabi-90, Metabolic Profiling and Its in Vitro Inhibitory Property against Multidrug Resistant and Extended-Spectrum Beta-Lactamase Clinical Bacterial Pathogens. J. Infect. Public Health.

[372] Alekish M., Ismail Z.B., Albiss B., Nawasrah S. (2018). In Vitro Antibacterial Effects of Zinc Oxide Nanoparticles on Multiple Drug-Resistant Strains of Staphylococcus Aureus and Escherichia coli: An Alternative Approach for Antibacterial Therapy of Mastitis in Sheep. Vet. World.

[373] Alsamhary K.I. (2020). Eco-Friendly Synthesis of Silver Nanoparticles by Bacillus Subtilis and Their Antibacterial Activity. Saudi J. Biol. Sci..

[374] Al-Dhabi N., Ghilan A.-K.M., Arasu M. (2018). Characterization of Silver Nanomaterials Derived from Marine Streptomyces Sp. Al-Dhabi-87 and Its In Vitro Application against Multidrug Resistant and Extended-Spectrum Beta-Lactamase Clinical Pathogens. Nanomaterials.

[375] Abdulmughni J., Mahyoub E.M., Alaghbari A.T., Al Serouri A.A., Khader Y. (2019). Performance of Multidrug-Resistant Tuberculosis Surveillance in Yemen: Interview Study. JMIR Public Health Surveill.

[376] Ganaie F., Nagaraj G., Govindan V., Basha R., Hussain M., Ashraf N., Ahmed S., Ravi Kumar K.L. (2018). Impact of Hajj on the S. Pneumoniae Carriage among Indian Pilgrims during 2016- a Longitudinal Molecular Surveillance Study. Travel Med. Infect. Dis..

[377] Leangapichart T., Gautret P., Griffiths K., Belhouchat K., Memish Z., Raoult D., Rolain J.-M. (2016). Acquisition of a High Diversity of Bacteria during the Hajj Pilgrimage, Including Acinetobacter Baumannii with *Bla*
_OXA-72_ and Escherichia coli with *Bla*
_NDM-5_ Carbapenemase Genes. Antimicrob. Agents Chemother..

[378] Zumla A., Saeed A.B., Alotaibi B., Yezli S., Dar O., Bieh K., Bates M., Tayeb T., Mwaba P., Shafi S. (2016). Tuberculosis and Mass Gatherings—Opportunities for Defining Burden, Transmission Risk, and the Optimal Surveillance, Prevention, and Control Measures at the Annual Hajj Pilgrimage. Int. J. Infect. Dis..

[379] Aurilio C., Sansone P., Paladini A., Barbarisi M., Coppolino F., Pota V., Pace M.C. (2021). Multidrug Resistence Prevalence in COVID Area. Life.

[380] Vaillancourt M., Jorth P. (2020). The Unrecognized Threat of Secondary Bacterial Infections with COVID-19. mBio.

