# Supplementary material for: Emerging Status of Multidrug-Resistant Bacteria and Fungi in the Arabian Peninsula
Source: Biology (Basel). 2021 Nov 6;10(11):1144. doi: 10.3390/biology10111144 (PMC8614875; doi:10.3390/biology10111144)
Supplement: Supplementary file 1 [file biology-10-01144-s001.zip › biology-1439421-supplementary.pdf]

biology

MDPI

Systematic Review

Emerging Status of Multidrug-Resistant Bacteria and Fungi in the Arabian Peninsula

J. Francis Borgio, Alia Saeed Rasdan, Bayan Sonbol, Galyah Alhamid, Noor B. Almandiland Sayed AbdulAzeez

Table S1. Country wise data for the number of articles from various database.

Country	Scopus	Pubmed	Google scholar	Scopus after 2010	Pubmed from >2010	Number of article screened	excluded	Full-text articles assessed for eligibility	Full-text articles excluded, with reasons	Final
Saudi Arabia	400	547	17360	312	498	622	296	326	128	198
Bahrain		7	979	17	3	3	0	3	0	3
Kuwait	138	98	5710	92	65	66	25	41	3	38
Oman	40	46	4170	36	42	42	33	9	0	9
Qatar	59	59	2069	51	54	52	35	17	0	17
United Arab Emirates	53	81	3090	38	71	70	51	19	4	15
Jordan	92	136	22400	78	115	81	44	37	6	31
Iraq	278	124	9410	223	78	107	26	81	6	75
Yemen	38	19	2269	30	17	14	6	8	2	6
	**1098**	**857**	**58047**	**877**	**828**	**1057**	**516**	**541**	**149**	**392***

* There were three papers reporting MDR microorganisms multiple countries. Sonnevend et al., 2016: Bahrain, Saudi Arabia and United Arab Emirates; Zowawi et al., 2015: Saudi Arabia, United Arab Emirates, Oman, Qatar, Bahrain, and Kuwait; Sonnevend et al., 2015: Saudi Arabia, Kuwait, Oman and United Arab Emirates.

Table S2. Number of multi drug resistant strains reported from countries of Arabian Peninsula.

S. No	Name of the MDR microbe	Saudi Arabia	Bahrain	Kuwait	Oman	Qatar	UAE	Jordan	Iraq	Yamen	Total Number of Reports
1	*Acinetobacter baumannii*	3787	8	410	224	426	16	372	461	3	85
2	*Escherichia coli*	10972	3	92	78	223	61	886	1017		90
3	*Klebsiella pneumoniae*	4092		222	520	24	55		668		63
4	*Mycobacterium tuberculosis*	9829		1176		223	1116	100	403	240	45
5	*Pseudomonas aeruginosa*	2594		18	27	178	31	31	528	65	60
6	*Staphylococcus aureus*	986		302	35	34		629	1182		25
7	*Streptococcus pneumoniae*	148				134		785	10		9
8	*Candida auris*	19		561							9
9	*Enterobacter cloacae*	49		5	8						13
10	*Enterobacteriaceae*	715		149			73		38		14
11	*Methicillin-resistant Staphylococcus aureus (MRSA)*	695		7138			6	140	99	60	23
12	*Stenotrophomonas maltophilia*	148			25		1				7
13	*Proteus mirabilis*	127							10		15
14	*Salmonella enterica*	33						287	93		4
15	*Serratia marcescens*	224		4					61		11
16	*Citrobacter freundii*	24			1				42		7
17	*Enterococcus faecium*	10		2	2				5		5
18	*Enterobacter*	812							3		9
19	*Enterococcus*	553									7
20	*Klebsiella oxytoca*	428		3					4		10
21	*Klebsiella spp*	927									8
22	*Listeria monocytogenes*							74	9		4
23	*Morganella morganii*	37							17		7
24	*salmonella spp*	10						71	4		8
25	*Salmonella typhi*			12					231		3
26	*Staphylococcus epidermidis*	32				26			1		4
27	*Staphylococcus spp*	78									5
28	*Candida albicans*	11									1
29	*Citrobacter koseri*	8							1		4
30	*Klebsiella aerogenes*										
31	*Staphylococcus hominis*	2				9					2
32	*Stenotrophomonus maltophilia*	257									6
33	*Enterococcus faecalis*	256									8
34	*Providencia stuartii*	10									3
35	*Streptococcus spp*	23									2
36	*Acinetobacter*	2014						19			15
37	*Acinetobacter calcoaceticus baumannii*	10									1
38	*Bacillus subtilis*										
39	*Campylobacter jejuni*					73		32	20		3
40	*Candida glabrata*			2							1
41	*Non-typhoidal Salmonella*	248									2
42	*Pantoea spp*	4							95		3

43	*Proteus sp.*	217	1		2	8
44	*Providencia spp*	3			1213	3
45	*Staphylococcus capitis*	4				1
46	*Enterobacter aerogenes*	32			8	6
47	*Acinetobacter lwoffii*	11				3
48	*Staphylococcus saprophyticus*	12			8	3
49	*A. baumannii complex*	19				1
50	*A. baumannii/haemolyticus*	32				1
51	*Acinetobacter haemolyticus*	4				1
52	*Vancomycin resistant Enterococcus (VRE)*	255				4
53	*Proteus vulgaris*	722				3
54	*Pseudomonas luteola*	9				1
55	*Pantoea agglomerans*	9				1
56	*Serratia fonticola*	4				1
57	*methicillin-resistant Staphylococcus epidermidis (MRSE)*	1				1
58	*penicillin-resistant Streptococcus pyogenes*	1				1
59	*Bacillus spp.*	77				1
60	*Leclercia adecarboxylata*	1				1
61	*Pseudomonas spp.*	481				6
62	*Serratia spp.*	66				3
63	*Candida Spp.*	180				4
64	*Citrobacter spp.*	54				5
65	*Mycobacterium leprae*	1				1
66	*Chryseobacterium gleum*	1				1
67	*Vibrio vulnificus*	234				1
68	*Clostridioides difficile*	18				1
69	*Arcobacter butzleri*				100	1
70	*Arcobacter cryaerophilus*				20	1
71	*Prevotella spp*		14			1
72	*Vibrio cholerae*				20	1
73	*Nocardia crassostreae*			13		1
74	*Vibrio vulnificus*			23		1
75	*Enterococcus gallinarum*		1			1
76	*Salmonella Enteritidies*		15			2

Table S3. Source of isolates and Number of MDR microbial strains reported from Arabian Peninsula. .

Text citation	Number of Strain	Source of Isolates
Saudi Arabia		
Ahmed-Abakur et al., 2019	*61 Mycobacterium tuberculosis*	human/Sputum
AlJindan et al., 2021	*18 Clostridioides difficile*	human/ stool
Al-Zahrani et al., 2021	*35 Carbapenem-resistant Pseudomonas aeruginosa (CRPA)*	human
Alhaddad et al., 2018	*11 Acinetobacter baumannii*	human/(ICU) samples
Ejaz et al., 2020	*370 Enterobacteria*	human
El Mahalli et al., 2015	*80 M. tuberculosis*	human
Salem-Bekhit et al., 2012	*206 Enterococcus spp.*	human
Shah et al., 2019	*135 Acinetobacter baumannii*	human
Abd El Hafez et al., 2011	*29 Staphylococcus epidermidis*	human/blood culture, umbilical wound swabs and endotracheal aspirate specimens of neonates
Ahmad et al., 2015	*52 K. pneumoniae*	human/blood, urine, wound swabs and sputum
Khan et al., 2016	*121 P aeruginosa strains*	human/ Respiratory, urine, Surgical, blood, Genital, eye, ear, burn,
Ahmed et al., 2019	*89 Organism isolated; Escherichia coli 24, Klebsiella pneumoniae 11, Proteus mirabilis 4, Pseudomonas aeruginosa 4, Enterobacter cloacae 5, Morganella morganii 4, Pantoea agglomerans 4, Citrobacter freundii 4, Klebsiella oxytoca 3, Proteus vulgaris 2, Acinetobacter baumannii 3, Enterobacter aerogenes 3, Acinetobacter lwoffii 3, Serratia marcescens 3, Enterococcus faecalis 5, Staphylococcus saprophyticus 3, Enterococcus faecium 2, Staphylococcus aureus 2*	human/urine
Al Bshabshe et al., 2016	*105 Acinetobacter species; A. baumannii accounted for 49 , A. baumanniicomplex 19, A. baumannii/haemolyticus 32, Acinetobacter haemolyticus 4, Acinetobater lwoffii 1*	human/Upper respiratory tract,lower respiratory tract,subcutaneous tissue,skin,gastrointestinal tract,wound swab,swab,endotrachael tube,eyes,bone & bone marrow,body fluids,cerebrospinal fluid,abscess,genito-urinary tract,miscellaneous specimens
Al Mayahi et al., 2019	*131*	human/sputum
AL Qurainees et al., 2016	*1 M. tuberculosis*	human/sputum
Al Wutayd et al., 2018	*418*	human/urine
Al-Agamy et al., 2017	*27 Acinetobacter baumannii*	human
Al-Anazi et al., 2012	*1 A. baumannii*	human/blood culture
Hoang et al., 2021	*81 strains were isolated (1 carbapenem-resistant Acinetobacter baumannii, 12 MRSA, and 68 ESBL-E).*	human/ respiratory and rectal samples
Al-Ayed et al., 2016	*10*	human/blood,urinary tract,ventilator-associated pneumonia
Al-Dorzi et al., 2015	*9 Acinetobacter bacteremia*	human/ blood culture.
Al-Ghafli et al., 2018	*71 MDR-TB strains*	human/smear
Al-Obeid et al., 2015	*12 XDRAB strains Acinetobacter baumannii*	human
Al-Jindan et al., 2021	*2 C. auris*	human/urine culture

(Alamri et al., 2020)	*1 drug-resistant strain of P. aeruginosa was isolated*	human/blood culture
(Alamri et al., 2020)	*207 multi-drug-resistant A. baumannii strains isolated*	human/respiratory specimens,skin and soft tissue specimens,Blood,urine and sterile body fluids.
(AlAmri et al., 2020)	*103 MDR A. baumannii isolates*	human/respiratory specime(endotracheal tube aspirates, bronchial aspirates,sputum, and throat specimens),wound swabs, rectal swabs, blood,urine & other strains were collected from(catheter tips, skin, soft tissue, and body fluids)
Alanber et al., 2020	*77 MDR Bacillus strain*	nonhuman/milk samples
Al-Hamad et al., 2020	*51 CRAb straines*	nonhuman/various surfaces
Altalhi et al., 2010	*119 E.coli strains*	nonhuman/chicken
Abdel-Haleem et al., 2015	*1 (MDR) Stenotrophomonas maltophilia strain isolated*	human
Alateah et al., 2020	*1123 isolates Mycobacterium tuberculosis.*	human/ sputum, bronchoalveolar lavage, tracheal aspirate, tissues and biopsies, fine needle aspiration, abscesses, abdominal fluids, breast fluid, cerebrospinal fluid, spinal fluid, urine, knee fluid and synovial fluid, pericardial fluid, peritoneal dialysis, pleural fluid.
Albarrag et al., 2020	*188 MRSA strains*	human/nasal swabs
Albukhari et al., 2019	*45 bacterial and fungal pathogens were isolated*	human
Aldawsari et al., 2020	*12 K. pneumoniae, S. aureus , A.baumannii , and P. aeruginosa(2016)/ 12 E. coli, K. pneumoniae, S. aureus, and P. aeruginosa (2017)*	human
Algowaihi et al., 2016	*1 MDR Klebsiella pneumoniae*	human/ urinary tract infection
Alhussain et al., 2021	*90 P. aeruginosa*	human/ respiratory, urine, wound, blood and fluid
Alhussaini et al., 2016	*46 MRSA isolates*	human/ nasal swabs
Ali et al., 2017	*32 MDR A. baumannii*	human/ blood and wound infections
Alkofide et al., 2020	*130 MDR Enterobacteriaceae*	human.
Aljindan et al., 2015	*47 Acinetobacer baumannii strains*	human/ rectal swab
AlJindan et al., 2020	*6 Candida auris*	human/ blood, urine, ear swab, groin screening samples
Almaghrabi et al., 2020	*8 Candida auris*	human/ Pleural tissue,urine,wound swab,Surgical,Tracheal aspirate
Almaghrabi et al., 2018	*94 A. baumannii*	human
Khan et al., 2019	*120 Enterobacteriaceae isolates*	human/ blood, Pus, Stool, Urine, Sputum, Endotracheal tube, Vaginal swab, Peritoneal fluid.
Hassan et al., 2014	*382 Enterobacteriaceae clinical isolates*	human/ urine, wounds , blood, central venous lines, sputum , sterile body fluids, ear infections
Hassan et al., 2013	*236 isolates*	human/ wound swab, CSF, Sputum, blood, urine, other
Farman et al., 2019	*155 Enterococcus faecalis isolates*	human
Alosaimi et al., 2020	*1 multidrug-resistant strain of Leclercia adecarboxylata*	human
Alqasim et al., 2018	*100 E. coli isolates*	human/ urine
Alqasim et al., 2021	*40 E. coli isolates*	human/ urine
Alsanie et al., 2020	*23 K. pneumoniae strains*	human
Alshareef et al., 2020	*91*	human/ urine culture
Alshukairi et al., 2020	*4 MDR-TB*	human/ sputum
Altuwaijri et al., 2020	*103 TB*	human

Alyamani et al., 2019	*69 M. tuberculosis isolates*	human/ sputum
Elhassan et al., 2017	*622 Mycobacterium tuberculosis isolates*	human/ sputum
Almutairy et al., 2020	*121*	human
Aly et al., 2016	*503 A. baumannii isolates*	human/ Tracheal, Unk, blood, urine, Sputum, Bronchial, Tissue.
El-Kersh et al., 2016	*Enterococcus faecalis (n=73) and Staphylococcus spp. (n=18)*	human/ faeces
EL-AGEERY et al., 2012	*MRAB isolates (15 patient samples and 3 environmental samples)*	human/ blood,Wound swab, Sputum, Abdominal drainfluid, Tracheal aspirate; environmental samples/ Suction switch, Ventilator button
El Ghany et al., 2018	*10 MDR Escherichia coli isolates*	human
BOURGHLI et al., 2019	*1 multidrug-resistant Pseudomonas aeruginosa.*	human
(Balkhy et al., 2020)	*1544*	human
Bosaeed et al., 2020	*19 MDR P aeruginosa*	human/ Respiratory, Skin tissue, Urine, Intra-abdominal fluid, Bone tissue, blood.
(Balkhy et al., 2020)	*1260*	human
El-Saed et al., 2020	*84*	human
Lagha et al., 2021	*30 K. pneumoniae isolates*	human/ blood,Wound swab, Sputum, urine.
Lopes et al., 2015	*46 A. baumannii isolated*	human/ Respiratory secretions, sputum, urine, Endotracheal tube aspirate, Wound swab, Tracheal swab.
Marie et al., 2013	*4250 isolates*	human/ blood, urine, wounds, sputum and other body fluids.
Marie et al., 2015	*54 A. baumannii isolates.*	human/ blood and respiratory samples.
Mazi et al., 2014	*20 isolates*	human
Mazi et al., 2020	*28 MRSA isolates*	human/ skin soft tissue, blood, Nasal, BURN, PNEU, SSI.
Rana et al., 2014	*2 MDR strains of E. coli and A. baumannii*	human/ blood, urine, Pleural fluid.
Salahuddin et al., 2016	*41*	human
Sambas et al., 2020	*8 MDR TB*	human/ sputum
Senok et al., 2015	*12 MDR A. baumannii isolates*	human/ urine, wound swab, sputum, endotracheal tube aspirates.
Shibl et al., 2013	*60 K. pneumoniae isolates*	human/blood, rectal swabs , sputum, urine, wound, and suction swab.
Somily et al., 2012	*117 MDR strains (84 isolates of A. baumannii and 33 isolates of P. aeruginosa)*	human/ blood/sterile body fluids and tissue, Superficial swabs and catheter tips, Urine, Wounds , sputum, bronchoalveolar lavage and tracheal aspirates.
Sonnevend et al., 2016	*1 E. coli isolates*	human/ blood
Taha et al., 2018	*76 MDR E. coli isolates*	human/ wound, urine, stool and blood.
Taher et al., 2019	*415 isolates*	human/ urine.
Varghese et al., 2013	*98 TB isolates*	human
Varghese et al., 2014	*135 MDR TB*	human
Varghese et al., 2017	*83 MDR-TB isolates*	human
Vellappally et al., 2017	*150 S. aureus isolates*	human/ dental caries samples.
Vijayakumar et al., 2018	*44 MDR*	human
Zikri et al., 2019	*1 MDR Pseudomonas aeruginosa*	human
Yasir et al., 2019	*1 Acinetobacter baumannii isolate*	human
(Yasir et al., 2020) Genomic and antimicrobial resistance genes diversity in	*19 ESBL-positive E. coli isolates*	human/ urine and tissue swabs.

multidrug-resistant CTX-M-positive isolates of Escherichia coli at a health care facility in Jeddah.		
Yasir et al., 2018	*211 ESBL-producing E. coli isolates*	human/ urine, urine catheters, wound swabs, blood samples.
Rabaan et al., 2017	*9 MDR Acinetobacter*	human/ nose swab, wound swab.
Olaitan et al., 2015	*5 MDR Salmonella spp.*	human/ rectal swabs.
Shin et al., 2011	*9 S. pneumoniae isolates*	human
Varghese et al., 2012	*151 M. tuberculosis isolates*	human
Bindayna et al., 2010	*100 isolates; E. coli =84, K. pneumoniae = 16*	human/ blood, Wound, urine, other body sites.
Aly et al., 2014	*253 isolates (MDR) Acinetobacter baumannii strains*	human/ blood, Wound, urine, burn, respiratory, others.
Aljadani et al., 2019	*2 MDR-TB*	human
Al-Hajoj et al., 2013	*1,609 isolates TB*	human
Almazrou et al., 2016	*46 S. pneumoniae isolates*	human/ Blood, Cerebro-spinal fluid, Pleural fluid
Hamid et al., 2011	*210 S.aureus isolates*	human
Hameed et al., 2019	*202 isolates*	human/ urine.
Al Johani et al., 2010	*4192 isolates*	human/ Respiratory, urine sample.
Khan et al., 2016 (The impact of onset time on the isolated pathogens and outcomes in ventilator associated pneumonia)	*293 VAP isolated*	human/ endotracheal aspiration sampling.
Guan et al., 2020	*1 MDR Mycobacterium leprae*	human
Almangour et al., 2020	*86 isolates*	human/ bronchoalveolar lavage, mini bronchoalveolar lavage, protected specimenbrush, endotracheal aspirate, sputum induction, nasotracheal suctioning.
Abdelfattah et al., 2019	*4 organisms isolated*	human/ blood culture.
Abulreesh et al., 2017	*50 S. aureus isolates*	human/ blood cultures, wound swabs, urine sample, nasal swabs, and sputum.
Alavudeen et al., 2017	*15 strains isolated*	human/ urine, blood, sputum, Endotracheal tube, Wound, Tracheal secretion, Throat swab, Abscess, Bedsore swab, Nasal swab, Rectal swab,Skin swab, Stool.
Albarrag et al., 2020	*17 MRSA isolates*	human/ nasal swabs.
Alghizzi et al.,2021	*94 S. aureus isolates*	non-human / Turkey, Salami, Sausages.
Alghoribi et al., 2019	*200 isolates Non-typhoidal Salmonella*	human/ Stool, Urine, Tissue, Abdominal fluid, Wound, Blood.
Alharbi et al., 2015	*18 A. baumannii strains*	human
AlOtair et al., 2015	*71 isolates*	human/ sputum
Asaad et al., 2012	*68 M. tuberculosis isolates*	human/ sputum, wound swabs, urine, ENT swabs, blood, trachial swab, genital swab, body fluid, tip catheter.
Alsanie et al.,2018	*30 MDR isolates*	human/ urine and stool swabs
Alyamani et al 2016	*1 E.coli strain*	human
Asghar et al., 2012	*478 Pseudomonas aeruginosa isolates*	human/ sputum, wound swabs, urine, ENT swabs, blood, trachial swab, genital swab, body fluid, tip catheter.
Azab et al., 2021	*162 isolates*	human/ urine, vaginal swab, ear swab, blood, abscess, endotracheal tube, sputum, throat swab, nasal swab, urethral swab.
Balkhy et al., 2012	*177 isolates Acinetobacter spp.*	human

El-Ahmady et al., 2016	*130 actinomycete straines*	nonhuman/ soil sample
Elsohaby et al., 2021	*20 isolates*	nonhuman/ birds
George et al., 2021	*246 isolates of enterococci (VRE)*	human/ urine, pus, rectal swab
Gowda et al., 2014	*380 A. baumannii isolates*	human / blood
Hassan et al., 2014	*154 isolates*	nonhuman/ animals fecal samples
Ibrahim et al., 2018	*290 Gram-negative isolates*	human/ Wound swabs, sputum, tracheal aspirates, umbilical swabs, throat and eye swabs, urine, blood.
Khan et al., 2018	*157 strains isolated*	human and hospital environment/ swabs were collected from a range of body sites from patients, staff and from the hospital environment like walls, beds, taps, computer keyboards and ventilator surfaces.
Marie et al., 2013	*4250 isolates*	human/ blood, urine, wounds, sputum and other body fluids.
Mazi et al., 2021	*15 isolates*	human
Sambas et al., 2020	*158 TB*	human
Varghese et al., 2013	*1904 isolates MDR-TB*	human
Yassin et al., 2020	*3 Candida strains*	human/ vaginal swabs
Zowawi ey al., 2015	*80 A. baumannii isolates*	human/ Blood, urine, swab, sputum.
Abdalhamid et al., 2016	*1 Chryseobacterium gleum*	human/ Blood.
Abdalla et al., 2013	*150 acinetobacter isolates*	human/ nasal swab, blood.
Ahmad et al., 2012	*916 gram-negative bacilli isolated*	human/ urine, pus, sputum, blood
Ahmed et al., 2015	*906 Acinetobacter baumanii isolates, 3626 other isolates*	human/ sputum,blood,urine, Throat Swab, Rectal Swab, Wound Swab, Tracheal Aspirates, Endo, BAL, eye, ear, tissue, fluid, abscess.
Al-Agamy et al., 2019	*6 strains isolated: (4) being Klebsiella pneumoniae and (2) Enterobacter cloacae.*	human/ blood, sputum,wound.
Al-Baloushi et al., 2018	*1 K. pneumoniae isolate*	human/ Endotracheal aspirate.
Al-Hajoj et al., 2015	*381 TB isolates*	human
Al-Mazrou et al., 2014	*(6) S. pneumoniae isolates, (8) H. influenzae isolates*	human/ Middle ear fluid.
Al-Qahtani et al., 2014	*98 Klebsiella pneumoniae isolates*	human/ blood, sputum, Wound swab, urine, HVS.
Alabdullatif et al., 2020	*4760 isolated gram-negative bacteria*	human/ sputum, vaginal, urine, blood, trachial,wound, throat.
**Al-Waili et al., 2012**	*S. aureus, E. coli and C.albicans isolates*	human
Alanazi et al., 2018	*101 E. coli isolates*	human/ urine.
Alghoribi et al., 2020	*1 K. pneumoniae*	human/ urine.
Aljindan et al., 2018	*60 isolates of A. baumannii*	human/ Abscess, blood, urine, Wound swab, Throat swab, Transtracheal, aspiration, Intravenous, catheter tip, Nasal Swab, Peritoneal fluid, Pleural fluid, Rectal swab, Skin swab, sputum.
Alkharsah et al., 2018	*106 MRSA isolates*	human/ Scalp abscess, Endotracheal aspirate, Nasal swab, Breast abscess, Skin swab (cellulitis), urine, wound swab.
Alnimr et al., 2017	*77 S. pneumoniae isolate*	human
Alraddadi et al., 2019	*10 Klebsiella pneumoniae, 28 Escherichia coli.*	human
Alsalem et al., 2018	*234 isolates of V. vulnificus.*	nonhuman/ seawater samples
Azim et al., 2019	*70 isolates/ (25) P.aeruginosa, (23) K. pneumoniae, (16) E. coli, (6) A. baumannii.*	human

Balkhy et al., 2014	*248 isolates; Acinetobacter spp.(n = 87), P. aeruginosa (n = 63), Staphylococcus aureus including methicillin-resistantStaphylococcus aureus (MRSA) (n = 43), Klebsiella spp.(n = 15), Enterobacter spp.(n = 10), Haemophilus spp.(n = 9), Stenotrophomonas maltophilia (n = 8),Coagulase-negative staphylococci (n = 7) and Escherichia coli(n = 6)*	human
Chaudhry et al., 2012	*1681 M. tuberculosis isolates*	human
Eed et al., 2019	*143 H. pylori*	human
El-Ageery et al., 2011	*154 MRSA isolates*	human and environmental sites.
El-Ghareeb et al., 2020	*17 E. coli isolates*	nonhuman/ minced meat samples
El-Mahdy et al., 2017	*10 A. calcoaceticusbaumannii isolates*	human
El-Tayeb et al., 2017	*33 S. enterica strains*	(human/ stools, urine and blood samples) & (nonhuman/ Sewage Treatment Plant)
Fadlelmula et al., 2016	*59 E. coli isolates*	human & nonhuman (camel)
Garaween et al., 2016	*48 salmonella isolates*	human/stool, blood, perianal abscess, urine,wound, chest drain and bone marrow.
Hala et al., 2019	*286 MDR Klebsiella spp. isolates*	human
Hassan et al., 2016	*12 isolates of Enterococcus spp.*	human
Helmi et al., 2013	*98 Gram-positive isolates (MRSA)*	human/ urine, blood, wound and respiratory secretion.
Ibrahim et al., 2014	*120 MDR E. coli isolates*	huamn/ urine, Wound pus, Vaginal swab, Blood culture, Stool, Ear swab.
Khairy et al., 2011	*5 isolates*	human
Memish et al., 2015	*242 isolates Gram-negative bacteria (Enterobacteriaceae, P. aeruginosa and A. baumannii)*	human
Pal et al., 2017	*32 Escherichia coli Isolates*	human
Saeed et al., 2010	*319 isolates*	human
Shobrak et al., 2013	*118 bacterial isolates*	nonhuman/ birds
(Somily et al., 2010)	*83 gram positive isolates & 254 MDR gram negative isolates .*	human
(Somily et al., 2010)	*273 isolates*	human
Somily et al., 2014	*568 Mycobacterium tuberculosis isolated.*	human
Varghese et al., 2013	*322 drug-resistant TB isolates*	human
(Varghese et al., 2013)	*524 Mycobacterium tuberculosis isolates*	human
Zaman et al., 2014	*23 Klebsiella pneumoniae isolates*	human
Zaman et al., 2018	*71 CRKP isolates*	human
Abulreesh et al., 2011	*37 staphyloccoccus aureus*	nonhuman/ food and environment in makkah
Nasser et al., 2020	*39 P. aeruginosa isolates*	human
Abdalhamid., 2018	*3 C. auris*	human
Oman		
Sonnevend et al., 2016	-	Human / blood/ urine/ wound

Balkhair et al., 2015	*total =329; 107 Acinetobacter baumannii ; 60 Escherichia coli (ESBL); 35 Methicillin-resistant Staphylococcus aureus; 34 Klebsiella pneumoniae (ESBL); 27 Pseudomonas aeruginosa; 26 Klebsiella pneumoniae (CRE); 25 Stenotrophomonas maltophilia; 5 Escherichia coli (CRE); 5 Burkholderia cepacia; 4 Enterobacter cloacae; 2 Enterococcus faecalis/faecium (VRE)*	Human / Bloodstream infection/ Pneumonia/ Urinary tract infection/ Surgical infection
Alkharousi et al., 2019	*14 enterobacteria/ 6 E.coli, 4 Enterobacter cloacae, one of each isolate each of Citrobacter freundii, Enterobacter asburiae, Enterobacter hormaechei, and Enterobacter ludwigii*	Non-Human / fresh fruits and vegetables.
Sonnevend et al., 2015	*Strains No.1, 2, 4, 6, 13, 15 K. pneumoniae and 3 E. coli*	Human / respiratory/ blood / urine/ screeing
Zowawi et al.,2015	*117 A. baumannii*	Human
Poirel et al.,2010	*419 Klebsiella pneumoniaes .*	Human / urinary catheter
Alwaidy et al., 2018	-	Human
pal et al., 2017	*4 MDR E.coli strain*	Human
Metry et al, 2017	-	Human
Qatar		
Taj Aldeen et al., 2013	*13 Nocardia isolates (from 2006 to June 2010.)*	Human / pus aspirate / sputum/ corneal/ scraping/ swab/ blood
Elshafie at al., 2016	*134 Streptococcus pneumoniae*	Human / blood and cerebrospinal fluid specimens
Garcell et al., 2016	*12 beta-lactamase producers (ESBL) Escherichia coli, 4 Pseudomonas aeruginosa, and 1 E. coli, 1 Enterococcus faecalis, 1 Klebsiella pneumoniae (ESBL) and 1 group B Streptococcus*	Human
Alsamawi et al., 2016	*372 A. baumannii*	Human / Blood/ Respiratory tract/ Urine
O. Eltal et al., 2018	*90 E.coli*	Non-Human / chicken cloacal samples
Khan et al., 2014	*10 Klebsiella pneumoniae.*	Human / sputum, blood, wound , urine
Ahmed et al., 2020	*75 MDR-P. aeruginosa*	Human
Rolain et al., 2016	*48 MDR A. baumannii*	Human
AbdulWahab et al., 2014	*16,11, 12,3 P. aeruginosa.*	Human / sputum or deep-pharyngeal swab samples from CF patients and non CF patients and other Environmental .
Ali et al., 2020	*223 MDR-TB. (patients number)*	human
AbdulWahab et al., 2017	*61 Pseudomonas aeruginosa*	Human / lower respiratory samples of pediatric and adult CF patients
Al mulla et al., 2014	*116 isolated strains of Gram-positive and Gram-negative bacteria.*	Human / blood
O. Eltai et al., 2020	*17 E. coli*	Human / diarrheagenic stool positive samples
Khan et al., 2010	*167 total of gram postive and gram negative Bactria; 12 Acinetobacterspp, 21 Enterobacter, 97 Escherichia coli, 39 Klebsiella spp., 21 Pseudomonasaeruginosa, 39 SalmonellaTyphi, 13 Salmonellaspp, 53 Staphylococcusaureus, 33 CoagulasenegativeStaphylococci, 34 Streptococcuspneumoniae, 22 Streptococcispp, 19 Enterococcusspp*	Human / IV catheter/Genitourinary system/ Respiratory system/ Gastrointestinal system/ Wounds/ Nervous system/Skin/soft tissue/ Surgical sites
Ghunaim et al., 2015	*total 174, only 73 Campylobacter*	Human

Al-Dulaimi et al., 2019	*60 V. vulnificus only 23 V. vulnificus isolates from Qatar*	non- human / from Mercenaria mercenaria
Hasan et al., 2020	-	human/ blood culture and urine culture
United Arab Emirates		
Alatoom et al., 2017	*23 ESBL E. coli; 6 K. pneumoniae; 31 P. aeruginosa ;49 K. pneumoniae ; 11 E. coli*	Human / blood, and urine, and other body tissues and fluids including wounds and abdomen
Alatoom et al., 2018	-	Human / blood
Mechkarska et al., 2010	*6 strains of methicillin-resistant S. aureus (MRSA) strains and 5 multidrug-resistant Acinetobacter baumannii (MDRAB) strains*	Non-Human/ skin secretions of the tetraploid frog Xenopus borealis Parker(frog)
Nekidy et al., 2017	-	Human/ Urine culture
Narchi et al., 2010	*26 E.coli*	Human/ urine by bag specimen
Mechkarska et al., 2013	*6 MRSA/ 5 A. baumannii strains / 8 independent carbapenemase-producing isolates*	human / wounds of patients
Conlon et al., 2011	*5 independent colistinsusceptible A. baumannii strains*	non- human/ frog skin
Habous et al., 2021	*1116 M. tuberculosis*	Human
Alzarouni et al., 2010	-	Human
Conlon et al., 2010	*5 independent A. baumannii strains*	Non- human/ skin secretions of the midwife toad Alytes obstetricans
Sonnevend et al., 2016	*4 E. coli isolates ; 1 from UAE*	Human
Conlon et al., 2012	*Acinetobacter baumannii strain NM8; Stenotrophomonas maltophilia B32/4 strain/ RP62A/1 S. epidermidis, and RP62A/1 S. epidermidis*	Non- Human / from skin secretions of the midwife toad, Alytes obstetricans
pal et al., 2017	*41 NDM-producing Enterobacteriaceae*	Human
Abro et al., 2017	-	Human/ blood culture
Mutti et al., 2017	-	Human
Kuwait		
Abulhasan et al., 2020	*84 were Enterobacteriaceae/ 15 P aeruginosa/ 11 were Enterococcus faecalis/ Seven of the 15 Staphylococcus aureus isolates were MRSA*	Human / respiratory infection , wound infections , polymicrobial infections
Ahmad et al., 2011	*39 M. tuberculosis from the Middle Eastern*	Human
Al-Mutairi et al., 2018	*60 MDR-TB*	Human
Al-Mutair et al., 2019. - Occurrence of disputed rpoB	*242 M. tuberculosis*	Human / pulmonary , extrapulmonary, pleural fluid, lymph node, tissue, cerebrospinal fluid, and gastric aspirate, specimens collected from TB patients
Al-Mutairi et al., 2010	*125 M. tuberculosis*	Human
Al-Sweih et al., 2010	*94 Acinetobacter baumannii*	Human / respiratory tract infections, bloodstream infections, wound infections, urinary tract infections ,and miscellaneous infections.
Al-Mutair et al., 2019- Molecular characterization	*93 MDR-TB*	Human/ pulmonary and extra-pulmonary specimens
Albert et al., 2019	*9 Enteritidies and 11s.enteritidis, 6 S.typhimurium, and 6 S.typhimurium*	Human / blood culture

Alfadli et al., 2018	*41 total :17 Acinetobacter baumannii, 8 Klebsiella pneumoniae, 6 Staphylococcus aureus, 6 Pseudomonas aeruginosa, 2 Enterococcus faecalis, 1 Serratia marcescens, 1Enterococcus gallinarum*	Human
Alfouzan et al., 2020	*71(17 candidemic and 54 colonized) patients including 26 with candiduria / 7 environmental samples yielded C. auris.*	Human / obtained from axilla, groin, anterior nares, vascularline exit site, oropharynx, respiratory and/or urinary tract from patients with or without clinical signs ofinfection. Environmental samples from rooms/units occupied by all C. auris-infected/colonized patients: medical instruments,linen, walls/floor, furniture and high touch areas such as doorknob, bed railings/bedside drawer andtoilet faucet/flush handles
Jamal et al., 2013	*14 carbapenem non-susceptible enterobacterial/9 K. pneumoniae, 3 Escherichia coli, 1 Enterobactercloacae and 1 K. oxytoca*	Human / blood culture, urine, wound and central venous pressure Most patients were male .
Jamal et al., 2016	*764 Enterobacteriaceae- K. pneumoniae (n = 25), E. coli (n = 22), Morganella morganii(n = 6), Enterobacter cloacae (n = 5), Enterobacter aerogenes (n = 2), and Providencia stuartii (n = 1)*	Human / blood/ Urine/ Wound/ screening retactal swab / screening throat swab/ tissue / CVP tip
Khan et al., 2014	*2 cholesterol-dependent Candida glabrata (Kw1018/12 and Kw1154/12)*	Human / blood sample
Sonnevend et al., 2016	-	
Khan et al., 2018	*17 isolates Candida auris*	Human / blood sample
Sonnevend et al., 2019	*5 Klebsiella pneumoniae, 1 Enterobacter cloacae, 1 Escherichia coli*	Human
Wibberg., 2018	*A. baumannii strain K50*	Human / urine
Vali et al., 2014	*173 ESBL-producing K. pneumoniae*	Human / urine, bile, and catheter.
Pa´ l et al., 2017	*total 157, only 16 NDM-producing Enterobacteriaceae isolated from Kuwait*	Human
Dashti et al., 2014	*83 MDR E. coli*	Human
Alfouzan et al., 2019	*209 Staphylococcus aureus*	Human/ medical wards, surgical wards , Obstetrics & gynaecology ward, Intensive care units,
Udo et al., 2017	*total, 6,922 MRSA isolates*	Human / Skin and soft tissues, Nasal swabs, Blood, Urine, Throat swabs, ear swabs,
Zowawi et al. 2015	*117 isolates Acinetobacterbaumannii / only Kuwait (n 8)*	Human / swab specimens /sputum/ blood
Ahmad et al. 2016	*70 Mycobacterium tuberculosis isolates*	Human
Ahmad et al. 2019	*314 Candida auris*	Human
Al-Mutairi et al. 2020	*47 Mycobacterium tuberculosis*	Human
AlFouzan et al. 2012	*291 Staphylococcus aureus*	Human
Al-Mutairi et al. 2011	*48 Mycobacterium tuberculosis strains*	Human
AL-sweih et al. 2011	*250 Acinetobacter baumannii isolates*	Human
Hamza et al. 2018	*71 patients*	Human
Ibrahim et al. 2018	*954 patients*	Human
Jamal et al. 2014	*Acinetobacter baumannii (13), Haemophilus influenzae (2) Klebsiella pneumoniae (10), Klebsiella oxytoca (2) Legionella pneumophila (2), Proteus sp. (1) Pseudomonas aeruginosa (12), Serratia marcescens (3)*	Human

	Staphylococcus aureus (5)	
Jamal et al. 2015	*66 Enterobacteriaceae isolates*	Human
Khan et al. 2018	*280 Candida spp.*	Human
Lesho et al. 2016	*Salmonella enterica (-)*	Human
Mokaddas et al. 2016	*452 Mycobacterium tuberculosis Isolates*	Human
Nasser et al. 2018	*6 Acinetobacter baumannii strains*	Human
Sonnevend et al. 2015	*17 Klebsiella pneumoniae*	Human
	5 E. coli	
	3 Enterobacter cloacae +2 others	
Toprak et al. 2018	*14 Prevotella bivia, 1 Prevotella disiens, 1 Prevotella nigrescens*	Human
Emara et al. 2015	*1 Candida auris*	Human
Bahrain		
Sonnevend et al. 2016	*2 E. Coli strains*	Human
Thani, 2019	*1 E. Coli strain*	Human
Zowawi et al. 2015	*8 Acinetobacter Baumannii isolates*	Human
Jordan		
Abdel-Halim et al. 2019	*3 E. Coli strains*	Human
Alaboudi et al. 2020	*32 Campylobacter isolates*	Chickens
Älgå et al. 2018	*36 MDR bacteria*	Human
Al-lawama et al. 2016	*19 Acinetobacter spp.*	Human
Almomani et al. 2015	*119 Acinetobacter baumannii*	Human
Al-Zoubi et al. 2015	*358 Staphylococcus aureus isolates*	Human
Badran et al. 2015	*142 E. Coli*	Human
Batarseh et al. 2015	*116 Acinetobacter baumannii*	Human
Burjaq et al. 2020	*29 Salmonella spp.*	Irrigation water
Darwish et al. 2010	*2 E. Coli strains*	Human
Haddadin et al. 2019	*68 E. Coli strains, 70 E. Coli strains*	Human, Green vegetables
Ibrahim et al. 2019	*269 E. Coli strains*	Chicken
Nairoukh et al. 2018	*150 E. Coli strains*	Human
Nimri et al. 2011	*100 Mycobacterium tuberculosis strains*	Human
Obaidat et al. 2015	*156 Staphylococcus aureus isolates*	Fish
Obaidat et al. 2020	*53 L. monocytogenes 287 S. enterica 17 E. coli isolates*	Beef cattle
Obeidat et al. 2014	*74 Acinetobacter baumannii*	Human
Osaili et al. 2013	*5 Salmonella*	Meat products
	21 Listeria monocytogenes	
Samrah et al. 2015	-	Human
Tarazi et al. 2020	*34 E. coli isolates*	Chicken
Tarazi et al. 2021	*15 Pseudomonas aeruginosa*	Drinking water
	16 Pseudomonas aeruginosa	Human
Abu Salah et al. 2013	*52 E. Coli*	Human
Al-Dabaibah et al. 2012	*63 A. baumannii*	Human
Alkhawaja et al. 2020	*Propionibacterium acnes, Staphylococcus aureus, Staphylococcus epidermidis*	Human

Al-Lahham et al. 2017	*241 Streptococcus pneumoniae*	Human
Burjaq et al. 2013	*17 E. Coli*	Vegetables
Ghanem et al 2018	*21 E. Coli*	Hospital and home environment
Ismail et al. 2017	*30 Staphylococcus aureus*	Cows milk
Nimri et al. 2014	*41 E. Coli, 37 Salmonella spp.*	Meat
Obaidat et al 2017	*140 Staphylococcus aureus*	dairy cattle, sheep, and goat bulk tank milk
Sallam et al. 2019	*544 Streptococcus pneumoniae*	Human
Iraq		
Ahmed et al. 2014	*270 Mycobacterium tuberculosis isolates*	Human
Ahmed et al. 2018	*25 Pseudomonas aeruginosa*	Human
Al-Delaimi et al. 2020	*20 Pseudomonas aeruginosa (patient samples)*	Human
Al-Guranie et al. 2020	*112 E. Coli strains, 17 MDR*	Human
Al-Kadmy et al. 2017	*21 Acinetobacter baumannii*	Human
Al-Mayahie et al. 2013	*61 E. Coli isolates*	Human
Al-Muhanna et al. 2016	*17 Morganella morganii isolates*	Human
Al-Rubaye et al. 2015	*110 isolates, 40 MDR Mycobacterium tuberculosis strains*	Human
Huang et al. 2012	*38 Enterobacteriaceae isolates, 13 MDR strains*	Human
Ibrahim et al. 2014	*10 E. Coli isolates*	Human
	10 E. Coli isolates	River water
Jasim et al. 2020	*100 Arcobacter butzleri*	Cattle meat product
	20 cryaerophilus	
Kareem et al. 2017	*30 Acinetobacter baumannii*	Human
Kusradze et al. 2011	*8 Acinetobacter baumannii strains*	Human
Mahdi et al. 2017	*14 Acinetobacter baumannii isolates*	Camel milk
Mahdi et al. 2019	*37 Pseudomonas aeruginosa*	Human
Merza et al. 2010	*12 Mycobacterium tuberculosis strains*	Human
Muslim et al. 2017	*51 Acinetobacter baumannii*	Human
Pishtiwan et al. 2019	*20 Klebsiella pneumoniae*	Human
	48 E. Coli	
Talat et al. 2020	*1 Staphylococcus epidermidis ST35 strain*	Human
AbdAlhussen et al. 2016	*40 Pantoea spp.*	Human and hospital environment
Abed et al. 2020	*142 E. Coli isolates*	Chicken
Ahmed et al. 2015	*3 Listeria monocytogenes*	Chicken
Al Zebary et al. 2017	*85 S. aureus isolates*	Human
Alaa et al. 2020	*87 E. coli*	Human and different animals
AL-Fatlawy et al. 2020	*231 Salmonella Typhi*	Human
Al-Gburi et al. 2020	*4 Providencia spp.*	Cow's milk
Alhamdani et al. 2020	*41 Pseudomonas aeruginosa*	Human and hospital environment
AL-Harmoosh et al. 2016	*10 A. baumannii*	Human
Ali et al. 2018	*148 E. Coli*	Human usinary tract
Ali et al. 2020	*40 Pseudomonas aeruginosa*	Human

Ali et al. 2021	*19 K. pneumoniae, 14 E. coli, 7 P. aeruginosa, 3 Enterbacter spp., 2 K. oxytoca, 2 A. baumannii, 1 Citrobacter koseri*	Human
Aljanaby et al. 2017	*122 P. aeruginosa, 83 K. pneumoniae, 32 E. Coli, 5 A. baumannii, 2 Proteus ssp.*	Human
Aljanaby et al. 2018	*80 K. pneumoniae*	Human
Aljanaby et al. 2018	*Staphylococcus saprophyticus 8, 109 Escherichia coli, Klebsiella pneumoniae 85, Acinetobacter baumannii 32, Pseudomonas aeruginosa 18, Serratia marcescens 11*	Human
AL-Khikani et al. 2020	*2 Klebsiella oxytoca, 16 K. pneumoniae*	Human
Alm'amoori et al. 2020	*49 E. Coli*	Human
Al-Mayahi et al. 2018	*1209 Providencia sp.*	Human, chickens, soil, wastewater , food
Al-Mayahi et al. 2020	*6 Listeria monocytogenes*	Human
AL-Muqdadi et al. 2019	*69 K. pneumoniae*	Human
Al-Ouqaili et al. 2018	*44 A. baumannii, 9 Pseudomonas aeruginosa*	Human
Al-Sa'ady et al. 2019	*5 E. faecalis*	Human
Al-Sa'ady et al. 2020	*10 Streptococcus pneumoniae*	Human
Alyousef et al. 2017	*97 K. pneumoniae*	Human
Awayid et al. 2019	*90 E. Coli*	Human
Azeez et al. 2019	*11 Acinetobacter baumani*	Human
Aziz et al. 2019	*99 Staphylococcus aureus*	Human
Babakir-Mina et al. 2012	*1063 Staphylococcus aureus*	Human
Behbahani et al. 2019	*63 Pseudomonas aeruginosa*	Human
Dawood et al. 2020	*12 E. Coli*	Human
Dibby et al. 2020	*60 Salmonella enterica*	Human
Fazaa et al. 2020	*55 Pantoea spp.*	Human
Flaifel et al. 2020	*60 Mycobacterium Tuberculosis*	Human
Ghaima et al. 2016	*48 Acinetobacter baumannii*	Human
Hammoudi et al. 2019	*18 K. pneumoniae, 10 P. aeruginosa, 6 P. mirabilis, 31 E. Coli*	Human
Hanan et al. 2019	*20 Vibrio cholerae*	Human
HARB et al. 2017	*33 Salmonella enterica*	Human
Hasan et al. 2019	*36 Pseudomonas aeruginosa*	Human
Hayder et al. 2019	*30 Citrobacter freundii*	Human
Jaaffar et al. 2019	*82 Pseudomonas aeruginosa*	Human
Jassim et al. 2020	*4 Salmonella spp.*	Milk
Kanaan et al. 2019	*35 Staphylococcus aureus*	Meat
Kanaan et al. 2020	*20 Campylobacter jejuni*	Chicken
Kareem et al. 2019	*P. aeruginosa, A. baumannii*	Human
Karomi et al. 2020	*13 E. Coli*	Human
Majeed et al. 2018	*E. coli 49, K. pneumonia 35, Pseudomonas aeruginosa 18, Citrobacter freundii 12, Enterobocter aerogenes 8, Proteus mirabilis 4*	Human
Qasim et al. 2019	*10 Acinetobacter Baumannii*	Human
Radhi et al. 2019	*21 Acinetobacter baumannii*	Human

Ridha et al. 2019	*124 Acinetobacter baumannii*	Human
Sahan et al. 2020	*50 Serratia marcescens*	Human
Salman et al. 2018	*66 Klebsiella pneumoniae*	Human
Shlash et al. 2018	*50 K. pneumoniae*	Human
Suhail et al. 2020	*21 Mycobacterium tuberculosis*	Human
Tuwaij et al. 2020	*30 K. pneumoniae*	Human
Wahaab et al. 2021	*Escherichia coli, Klebsiella pneumonia, Acinetobacter baumannii, Staph. Epidermidis, Staph. hemolyticus, Staph. aureus*	Human
Yemen		
Bakour et al. 2014	*3 Acinetobacter baumannii strains*	Human
Jaber et al 2019	*52 Mycobacterium tuberculosis isolates*	Human
Jaber et al. 2019	*115 Mycobacterium tuberculosis isolates*	Human
AL Baidani et al. 2011	*60 Staphylococcus aureus*	Human
Al-Mahbashi et al. 2012	*120 Mycobacterium tuberculosis*	Human
Nasser et al. 2019	*65 Pseudomonas aeruginosa*	Human

Reference

1 Abd El Ghany M, Sharaf H, Al-agamy MH, Shibl A, Hill-Cawthorne GA, Hong P-Y. Genomic characterization of NDM-1 and 5, and OXA-181 carbapenemases in uropathogenic Escherichia coli isolates from Riyadh, Saudi Arabia. *PLoS ONE* 2018; **13**: e0201613.

2 Abdel-Halim H, Al Dajani A, Abdelhalim A, Abdelmalek S. The search of potential inhibitors of the AcrAB–TolC system of multidrug-resistant Escherichia coli: an in silico approach. *Appl Microbiol Biotechnol* 2019; **103**: 6309–6318.

3 Abed AR, Khudhair AM, Hussein IM. Effects of Misuse of Antibiotics on the Resistance of Escherichia coli Isolated from the Intestines of Broiler Chickens. *ijddt* 2020; **10**: 190–194.

4 Ahmad S, Al-Mutairi NM, Mokaddas E. Variations in the occurrence of specific rpoB mutations in rifampicin- resistant Mycobacterium tuberculosis isolates from patients of different ethnic groups in Kuwait. *INDIAN J MED RES* 2012; : 8.

5 Ahmad Hamad P, Khadija KM. PREVALENCE OF BLATEM, BLASHV, AND BLACTX-M GENES AMONG ESBL-PRODUCING KLEBSIELLA PNEUMONIAE AND ESCHERICHIA COLI ISOLATED FROM THALASSEMIA IN ERBIL, IRAQ. *Mediterr J Hematol Infect Dis* 2019; **11**: e2019041.

6 Ahmed MU, Farooq R, Hawashim NA *et al.* Sensitive, resistant and multi-drug resistant Acinetobacter baumanii at Saudi Arabia hospital eastern region. *Pak J Pharm Sci* 2015; : 9.

7 Al Johani SM, Akhter J, Balkhy H, El-Saed A, Younan M, Memish Z. Prevalence of antimicrobial resistance among gram-negative isolates in an adult intensive care unit at a tertiary care center in Saudi Arabia. *Annals of Saudi Medicine* 2010; **30**: 364–369.

8 Al Wutayd O, Al Nafeesah A, Adam I, Babikir I. The antibiotic susceptibility patterns of uropathogens isolated in Qassim, Saudi Arabia. *J Infect Dev Ctries* 2018; **12**: 946–952.

9 Alaa AR, Solhan MA, Lalan RM *et al.* THE ANTIBIOTIC RESISTANCE PATTERN AND MOLECULAR CHARACTERIZATION OF blaCTX AND blaTEM GENES OF E. coli ISOLATED FROM DIFFERENT HOSTS BASED ON THE RATE OF ANTIBIOTIC CONSUMPTION IN SULAYMANIYAH/IRAQ. *Appl Ecol Env Res* 2020; **18**: 6025–6040.

10 Alanazi MQ, Alqahtani FY, Aleanizy FS. An evaluation of E. coli in urinary tract infection in emergency department at KAMC in Riyadh, Saudi Arabia: retrospective study. *Ann Clin Microbiol Antimicrob* 2018; **17**: 3.

11 Alatoom A, Elsayed H, Lawlor K *et al.* Comparison of antimicrobial activity between ceftolozane–tazobactam and ceftazidime–avibactam against multidrug-resistant isolates of Escherichia coli , Klebsiella pneumoniae , and Pseudomonas aeruginosa. *International Journal of Infectious Diseases* 2017; **62**: 39–43.

12 Alavudeen SS, Vigneshwaran E, Asiri SAA *et al.* Distribution of Multi-Resistant Bacterial Isolates from Clinical Specimens in a Hospital Environment of Kingdom of Saudi Arabia. *JYP* 2017; **9**: 347–351.

13 Al-Ayed MSZ, Asaad AM, Qureshi MA, Attia HG, AlMarrani AH. Antibacterial Activity of *Salvadora persica* L. (Miswak) Extracts against Multidrug Resistant Bacterial Clinical Isolates. *Evidence-Based Complementary and Alternative Medicine* 2016; **2016**: 1–5.

14 Albukhari TAM, Nafady-Hego H, Elgendy H, Abd Elmoneim HM, Nafady A, Alzahrani AM. Analysis of Bacterial and Fungal Infections after Cytoreduction Surgery and Hyperthermic Intraperitoneal Chemotherapy: An Observational Single-Centre Study. *International Journal of Microbiology* 2019; **2019**: 1–10.

15 Al-Guranie DR, Al-Mayahie SM. Prevalence of E. coli ST131 among Uropathogenic E. coli Isolates from Iraqi Patients in Wasit Province, Iraq. *International Journal of Microbiology* 2020; **2020**: 1–9.

16 Ali FA, Hussen BM, Zaki SM. MOLECULAR DETECTION OF BLACTX-M GENE AMONG PSEUDOMONAS AERUGINOSA STRAINS ISOLATED FROM DIFFERENT CLINICAL SAMPLES IN ERBIL CITY. *ATMPH* 2020; **23**. doi:10.36295/ASRO.2020.231231.

17 Aljanaby AAJ. Antibacterial activity of an aqueous extracts of Alkanna tinctoria roots against drug resistant aerobic pathogenic bacteria isolated from patients with burns infections. *Russ Open Med J* 2018; **7**: e0104.

18 Aljanaby AAJ, Tuwaij NSS, Al-khilkhali HJB. Antimicrobial susceptibility patterns of Klebsiella pneumoniae isolated from older smokers and non-smokers of inpatients in intensive care unit infected with chronic pneumonia in AL-Najaf hospital, Iraq. *J Pharm Sci* 2018; **10**: 6.

19 Alm'amoori K, Hadi Z, Almohana A. Molecular Investigation of Plasmid–Mediated Quinolone Resistant Genes among aminoglycoside-resistant uropathogenic Escherichia coli Isolates from Babylon Hospitals, Iraq. *Indian Journal of Forensic Medicine & Toxicology*; **14**: 493–498.

20 Al-Mayahie SM. Phenotypic and genotypic comparison of ESBL production by Vaginal Escherichia coli isolates from pregnant and non-pregnant women. *Ann Clin Microbiol Antimicrob* 2013; **12**: 7.

21 Al-Mulla N, Elshafie SS, Janahi M, Al-Nasser A, Chandra P, Taj-Aldeen SJ. Bacterial bloodstream infections and antimicrobial susceptibility pattern in pediatric hematology/oncology patients after anticancer chemotherapy. *IDR* 2014; : 289.

22 AlOtair HA, Hussein MA, Elhoseny MA, Alzeer AH, Khan MF. Severe pneumonia requiring ICU admission: Revisited. *Journal of Taibah University Medical Sciences* 2015; **10**: 293–299.

23 Alqasim A, Abu Jaffal A, Alyousef AA. Prevalence of Multidrug Resistance and Extended-Spectrum *β* -Lactamase Carriage of Clinical Uropathogenic *Escherichia coli* Isolates in Riyadh, Saudi Arabia. *International Journal of Microbiology* 2018; **2018**: 1–9.

24 Alshareef H, Alfahad W, Albaadani A, Alyazid H, Talib RB. Impact of antibiotic de-escalation on hospitalized patients with urinary tract infections: A retrospective cohort single center study. *Journal of Infection and Public Health* 2020; **13**: 985–990.

25 Alshukairi AN, Moalim HM, Alsaedi A *et al.* Family cluster of multi-drug resistant tuberculosis in Kingdom of Saudi Arabia. *Journal of Infection and Public Health* 2020; **13**: 154–157.

26 Altalhi AD, Gherbawy YA, Hassan SA. Antibiotic Resistance in *Escherichia coli* Isolated from Retail Raw Chicken Meat in Taif, Saudi Arabia. *Foodborne Pathogens and Disease* 2010; **7**: 281–285.

27 Awayid HS, Sahar BR, Jalil IS, Nouman KT. Immunization against Multi drug Resistance Uropathogenic *E. coli* Isolate from Urinary Tract Infection in Pregnancy. *Rese Jour of Pharm and Technol* 2019; **12**: 5444.

28 Azab KSM, Abdel-Rahman MA, El-Sheikh HH, Azab E, Gobouri AA, Farag MMS. Distribution of Extended-Spectrum β-Lactamase (ESBL)-Encoding Genes among Multidrug-Resistant Gram-Negative Pathogens Collected from Three Different Countries. *Antibiotics* 2021; **10**: 247.

29 Azim NSA, Al-Harbi MA, Al-Zaban MI, Nofal MY, Somily AM. Prevalence and Antibiotic Susceptibility among Gram Negative Bacteria Isolated from Intensive Care Units at a Tertiary Care Hospital in Riyadh, Saudi Arabia. *J Pure Appl Microbiol* 2019; **13**: 201–208.

30 Badran EF, Din RAQ, Shehabi AA. Low intestinal colonization of Escherichia coli clone ST131 producing CTX-M-15 in Jordanian infants. *Journal of Medical Microbiology* 2016; **65**: 137–141.

31 Balkhy HH, El-Saed A, Alshamrani MM *et al.* Ten-year resistance trends in pathogens causing healthcare-associated infections; reflection of infection control interventions at a multi-hospital healthcare system in Saudi Arabia, 2007–2016. *Antimicrob Resist Infect Control* 2020; **9**: 21.

32 Balkhy HH, El-Saed A, Alshamrani MM *et al.* High Burden of Resistant Gram Negative Pathogens Causing Device-associated Healthcare Infections in a Tertiary Care Setting in Saudi Arabia, 2008-2016. *Journal of Global Antimicrobial Resistance* 2020; **23**: 26–32.

33 Balkhy H, El-Saed A, Maghraby R *et al.* Drug-resistant ventilator associated pneumonia in a tertiary care hospital in Saudi Arabia. *Ann Thorac Med* 2014; **9**: 104.

34 Bindayna KM, Khanfar HS, Senok AC, Botta GA. Predominance of CTX-M genotype among extended spectrum beta lactamase isolates in a tertiary hospital in Saudi Arabia. ; : 5.

35 Burjaq SZ, Shehabi A. Fresh leafy green vegetables associated with multidrug resistant E.coli. *int arab j antimic agents* 2013; **3**: 1–7.

36 Darwish RM, Aburjai TA. Effect of ethnomedicinal plants used in folklore medicine in Jordan as antibiotic resistant inhibitors on Escherichia coli. *BMC Complement Altern Med* 2010; **10**: 9.

37 Dashti AA, Vali L, El-Shazly S, Jadaon MM. The characterization and antibiotic resistance profiles of clinical Escherichia coli O25b-B2-ST131 isolates in Kuwait. *BMC Microbiol* 2014; **14**: 214.

38 Dawood WS. Molecular and susceptibility Study of Antibiotic Resistance Genes in E. coli Isolated from Selected Iraqi Patients. *Systematic Reviews in Pharmacy* 2020; **11**: 10.

39 El-Ghareeb WR, Abdel-Raheem SM, Al-Marri TM, Alaql FA, Fayez MM. Isolation and identification of extended spectrum β-lactamases (ESBLs) Escherichia coli from minced camel meat in Eastern province, Saudi Arabia. *Thai J Vet Med*; : 8.

40 El-Saed A, Balkhy HH, Alshamrani MM *et al.* High contribution and impact of resistant gram negative pathogens causing surgical site infections at a multi-hospital healthcare system in Saudi Arabia, 2007–2016. *BMC Infect Dis* 2020; **20**: 275.

41 Elsohaby I, Samy A, Elmoslemany A *et al.* Migratory Wild Birds as a Potential Disseminator of Antimicrobial-Resistant Bacteria around Al-Asfar Lake, Eastern Saudi Arabia. *Antibiotics* 2021; **10**: 260.

42 Eltai NO, Abdfarag EA, Al-Romaihi H *et al.* Antibiotic Resistance Profile of Commensal Escherichia coli Isolated from Broiler Chickens in Qatar. *Journal of Food Protection* 2017; **81**: 302–307.

43 Eltai NO, Al Thani AA, Al Hadidi SH, Al Ansari K, Yassine HM. Antibiotic resistance and virulence patterns of pathogenic Escherichia coli strains associated with acute gastroenteritis among children in Qatar. *BMC Microbiol* 2020; **20**: 54.

44 Fadlelmula A, Al-Hamam NA, Al-Dughaym AM. A potential camel reservoir for extended-spectrum β-lactamase-producing Escherichia coli causing human infection in Saudi Arabia. *Trop Anim Health Prod* 2016; **48**: 427–433.

45 Garcell HG, Arias AV, Pancorbo Sandoval CA *et al.* Incidence and Etiology of Surgical Site Infections in Appendectomies: A 3-Year Prospective Study. *Oman Med J* 2017; **32**: 31–35.

46 Ghanem B, Haddadin RN. Multiple drug resistance and biocide resistance in Escherichia coli environmental isolates from hospital and household settings. *Antimicrob Resist Infect Control* 2018; **7**: 47.

47 Haddadin RN, Assaf AM, Homsi A, Collier PJ, Shehabi A. Investigating possible association between multidrug resistance and isolate origin with some virulence factors of *Escherichia coli* strains isolated from infant faeces and fresh green vegetables. *J Appl Microbiol* 2019; **127**: 88–98.

48 Hameed T, Al Nafeesah A, Chishti S, Al Shaalan M, Al Fakeeh K. Community-acquired urinary tract infections in children: Resistance patterns of uropathogens in a tertiary care center in Saudi Arabia. *International Journal of Pediatrics and Adolescent Medicine* 2019; **6**: 51–54.

49 Hammoudi A. Antibiotics susceptibility pattern of some Enterobacteriaceae isolates from different clinical infectious sources. *International Journal of Research in Pharmaceutical Sciences* 2019; **10**: 734–741.

50 Hassan H, Abdalhamid B. Molecular characterization of extended-spectrum beta-lactamase producing Enterobacteriaceae in a Saudi Arabian tertiary hospital. *J Infect Dev Ctries* 2014; **8**: 282–288.

51 Huang X-Z, Frye JG, Chahine MA *et al.* Characteristics of Plasmids in Multi-Drug-Resistant Enterobacteriaceae Isolated during Prospective Surveillance of a Newly Opened Hospital in Iraq. *PLoS ONE* 2012; **7**: e40360.

52 Ibrahim MM, Abuelmatty AM, Mohamed GH *et al.* Best tigecycline dosing for treatment of infections caused by multidrug-resistant pathogens in critically ill patients with different body weights. *DDDT* 2018; **Volume 12**: 4171–4179.

53 Ibrahim RA, Cryer TL, Lafi SQ, Basha E-A, Good L, Tarazi YH. Identification of Escherichia coli from broiler chickens in Jordan, their antimicrobial resistance, gene characterization and the associated risk factors. *BMC Vet Res* 2019; **15**: 159.

54 Ibrahim I, Al- Shwaikh R, Ismaeil M. Virulence and antimicrobial resistance of Escherichia coli isolated from Tigris River and children diarrhea. *IDR* 2014; : 317.

55 Ibrahim ME, Bilal NE, Hamid ME. Comparison of Phenotypic Characteristics and Antimicrobial Resistance Patterns of Clinical Escherichia coli Collected From Two Unrelated Geographical Areas. *GJHS* 2014; **6**: p126.

56 Jaloob Aljanaby AA, Aljanaby IAJ. Antimicrobial sensitivity pattern of pathogenic bacteria isolated from older women with asymptomatic bacteriuria. *biomedicalresearch* 2018; **29**. doi:10.4066/biomedicalresearch.29-18-601.

57 Jamal W, Rotimi VO, Albert MJ, Khodakhast F, Nordmann P, Poirel L. High prevalence of VIM-4 and NDM-1 metallo-β-lactamase among carbapenem-resistant Enterobacteriaceae. *Journal of Medical Microbiology* 2013; **62**: 1239–1244.

58 Karomi ASA. Screening Study for Some Strains of E. Coli Collected from Five regions in Kurdistan-Iraq for its Sensitivity, Resistance and MDR against Thirteen Antibiotics. *MLU* 2020; **20**: 526–531.

59 Khairy GA, Kambal AM, Al-Dohayan AA *et al.* Surgical Site Infection in a Teaching Hospital: A Prospective Study. *Journal of Taibah University Medical Sciences* 2011; **6**: 114–120.

60 Khan FY, Elshafie SS, Almaslamani M *et al.* Epidemiology of bacteraemia in Hamad general hospital, Qatar: A one year hospital-based study. *Travel Medicine and Infectious Disease* 2010; **8**: 377–387.

61 Khan Z, Ahmad S, Al-Sweih N, Joseph L, Alfouzan W, Asadzadeh M. Increasing prevalence, molecular characterization and antifungal drug susceptibility of serial Candida auris isolates in Kuwait. *PLoS ONE* 2018; **13**: e0195743.

62 Majeed HT, Aljanaby AAJ. Antibiotic Susceptibility Patterns and Prevalence of Some Extended Spectrum Beta- Lactamases Genes in Gram-Negative Bacteria Isolated from Patients Infected with Urinary Tract Infections in Al-Najaf City, Iraq. 2019; **11**: 10.

63 Marie MA, John J, Krishnappa LG, Gopalkrishnan S. Molecular characterization of the β-lactamases in *Escherichia coli* and *Klebsiella pneumoniae* from a tertiary care hospital in Riyadh, Saudi Arabia: β-Lactamases in *Enterobacteriaceae*. *Microbiol Immunol* 2013; **57**: 805–810.

64 Mazi W, Begum Z, Abdulla D *et al.* Central line–associated bloodstream infection in a trauma intensive care unit: Impact of implementation of Society for Healthcare Epidemiology of America/Infectious Diseases Society of America practice guidelines. *American Journal of Infection Control* 2014; **42**: 865–867.

65 Memish ZA, Assiri A, Almasri M *et al.* Molecular Characterization of Carbapenemase Production Among Gram-Negative Bacteria in Saudi Arabia. *Microbial Drug Resistance* 2015; **21**: 307–314.

66 Mutti M, Sonnevend Á, Pál T *et al.* Complete Genome Sequence of *Escherichia coli* 81009, a Representative of the Sequence Type 131 C1-M27 Clade with a Multidrug-Resistant Phenotype. *Genome Announc* 2018; **6**. doi:10.1128/genomeA.00056-18.

67 Nairoukh YR, Mahafzah AM, Irshaid A, Shehabi AA. Molecular Characterization of Multidrug Resistant Uropathogenic E. Coli Isolates from Jordanian Patients. *TOMICROJ* 2018; **12**: 1–7.

68 Narchi H, Al-Hamdani M. Uropathogen Resistance to Antibiotic Prophylaxis in Urinary Tract Infections. *Microbial Drug Resistance* 2010; **16**: 151–154.

69 Nimri L, Abu AL- Dahab F, Batchoun R. Foodborne bacterial pathogens recovered from contaminated shawarma meat in northern Jordan. *J Infect Dev Ctries* 2014; **8**: 1407–1414.

70 Nimri L, Samara H, Batchoun R. Detection of mutations associated with multidrug-resistant *Mycobacterium tuberculosis* clinical isolates. *FEMS Immunol Med Microbiol* 2011; **62**: 321–327.

71 Obaidat MM. Prevalence and antimicrobial resistance of Listeria monocytogenes, Salmonella enterica and Escherichia coli O157:H7 in imported beef cattle in Jordan. *Comparative Immunology, Microbiology and Infectious Diseases* 2020; **70**: 101447.

72 Pál T, Ghazawi A, Darwish D *et al.* Characterization of NDM-7 Carbapenemase-Producing *Escherichia coli* Isolates in the Arabian Peninsula. *Microbial Drug Resistance* 2017; **23**: 871–878.

73 Radwan Ali M, Mohammed Khudhair A. Detection of Colony Adhesion Factors and Genetic Background of Adhesion Genes Among Multidrug-Resistant Uropathogenic Escherichia coli Isolated in Iraq. *J Pure Appl Microbiol* 2018; **12**: 2017–2026.

74 Rana MA, Abd El Rahaman B, Mady AF *et al.* Intra-pleural colistin methanesulfonate therapy for pleural infection caused by carbapenem-resistant Acinetobacter baumannii: a successful case report. *Infect Dis Rep* 2014; **6**. doi:10.4081/idr.2014.5413.

75 Saeed NK, Kambal AM, El-Khizzi NA. Antimicrobial-resistant bacteria in a general intensive care unit in Saudi Arabia. ; : 10.

76 Salah M, Badran E, Shehabi A. High incidence of multidrug resistant Escherichia coli producing CTX-M-type ESBLs colonizing the intestine of Jordanian infants. *The International Arabic Journal of Antimicrobial Agents* 2014; **3**: 1–8.

77 Shobrak MY, Hassan SA, Stiévenart C, El-Deeb BA, Gherbawy YA. Prevalence and Antibiotic Resistance Profile of Intestinal Bacteria Isolated from Captive Adult Houbara Bustards ( ) Exposed to Natural Weather Conditions in Saudi Arabia. *Escherichia coli* 2013; : 9.

78 Sonnevend Á, Ghazawi AA, Hashmey R *et al.* Characterization of Carbapenem-Resistant Enterobacteriaceae with High Rate of Autochthonous Transmission in the Arabian Peninsula. *PLoS ONE* 2015; **10**: e0131372.

79 Sonnevend Á, Ghazawi A, Alqahtani M *et al.* Plasmid-mediated colistin resistance in Escherichia coli from the Arabian Peninsula. *International Journal of Infectious Diseases* 2016; **50**: 85–90.

80 Sonnevend Á, Ghazawi A, Alqahtani M *et al.* Plasmid-mediated colistin resistance in Escherichia coli from the Arabian Peninsula. *International Journal of Infectious Diseases* 2016; **50**: 85–90.

81 Sonnevend A, Yahfoufi N, Ghazawi A, Jamal W, Rotimi V, Pal T. Contribution of horizontal gene transfer to the emergence of VIM-4 carbapenemase producer Enterobacteriaceae in Kuwait. *IDR* 2017; **Volume 10**: 469–478.

82 Taha MME, Homeida HE, Dafalla OME, Abdelwahab SI. Multidrug resistance, prevalence and phylogenetic analysis of genes encoding class II and III integrons in clinically isolated Escherichia coli. *Cell Mol Biol (Noisy-le-grand)* 2018; **64**: 122.

83 Taher I, Almaeen A, Aljourfi H *et al.* Surveillance of antibiotic resistance among uropathogens in Aljouf region northern Saudi Arabia. *IJM* 2020. doi:10.18502/ijm.v11i6.2218.

84 Tarazi YH, Abu-Basha EA, Ismail ZB, Tailony RA. In vitro and in vivo efficacy study of cefepime, doripenem, tigecycline, and tetracycline against extended-spectrum beta-lactamases Escherichia coli in chickens. *Vet World* 2020; **13**: 446–451.

85 Thani ASB. Characterization of previously identified novel DNA fragment associated with Pathogenicity Island III536 reveals new bla gene. *Infection, Genetics and Evolution* 2019; **75**: 103971.

86 Ahmad S, Mokaddas E, Al-Mutairi N, Eldeen HS, Mohammadi S. Discordance across Phenotypic and Molecular Methods for Drug Susceptibility Testing of Drug-Resistant Mycobacterium tuberculosis Isolates in a Low TB Incidence Country. *PLoS ONE* 2016; **11**: e0153563.

87 Ahmed MM, Mohammed SH, Nasurallah HAA, Ali MM, Couvin D, Rastogi N. Snapshot of the genetic diversity of Mycobacterium tuberculosis isolates in Iraq. *International Journal of Mycobacteriology* 2014; **3**: 184–196.

88 Ahmed-Abakur E, Saad Alnour T. Detection of multidrug resistant Mycobacterium tuberculosis in Tabuk, Saudi Arabia, using Genotype MTBDRplus. *Int J Mycobacteriol* 2019; **8**: 25.

89 Al Mahbashi AA, Mukhtar MM, Mahgoub ES. Molecular typing of Mycobacterium spp. isolates from Yemeni tuberculosis patients. *East Mediterr Health J* 2013; **19**: 942–946.

90 AL Qurainees GI, Tufenkeji HT. A child with complicated Mycobacterium tuberculosis. *International Journal of Pediatrics and Adolescent Medicine* 2016; **3**: 28–33.

91 Alateah SM, Othman MW, Ahmed M, Al Amro MS, Al Sherbini N, Ajlan HH. A retrospective study of tuberculosis prevalence amongst patients attending a tertiary hospital in Riyadh, Saudi Arabia. *Journal of Clinical Tuberculosis and Other Mycobacterial Diseases* 2020; **21**: 100185.

92 Al-Ghafli H, Kohl TA, Merker M *et al.* Drug-resistance profiling and transmission dynamics of multidrug-resistant Mycobacterium tuberculosis in Saudi Arabia revealed by whole genome sequencing. *IDR* 2018; **Volume 11**: 2219–2229.

93 Al-Hajoj S, Varghese B, Shoukri MM *et al.* Epidemiology of Antituberculosis Drug Resistance in Saudi Arabia: Findings of the First National Survey. *Antimicrob Agents Chemother* 2013; **57**: 2161–2166.

94 Al-Hajoj S, Shoukri M, Memish Z, AlHakeem R, AlRabiah F, Varghese B. Exploring the Sociodemographic and Clinical Features of Extrapulmonary Tuberculosis in Saudi Arabia. *PLoS ONE* 2015; **10**: e0101667.

95 Ali M, Howady F, Munir W *et al.* Drug-resistant tuberculosis: an experience from Qatar. *Libyan Journal of Medicine* 2020; **15**: 1744351.

96 Ali Chaudhry L, Rambhala N, Al-Shammri AS, Al-Tawfiq JA. Patterns of antituberculous drug resistance in Eastern Saudi Arabia: A 7-year surveillance study from 1/2003 to 6/2010. *JEGH* 2011; **2**: 57.

97 Aljadani R, Ahmed AE, AL-Jahdali H. Tuberculosis mortality and associated factors at King Abdulaziz Medical City Hospital. *BMC Infect Dis* 2019; **19**: 427.

98 Al-Mutairi NM, Ahmad S, Mokaddas E, Eldeen HS, Joseph S. Occurrence of disputed rpoB mutations among Mycobacterium tuberculosis isolates phenotypically susceptible to rifampicin in a country with a low incidence of multidrug-resistant tuberculosis. *BMC Infect Dis* 2019; **19**: 3.

99 Al-Mutairi NM, Ahmad S, Mokaddas E. First report of molecular detection of fluoroquinolone resistance-associated gyrA mutations in multidrug-resistant clinical Mycobacterium tuberculosis isolates in Kuwait. *BMC Res Notes* 2011; **4**: 123.

100 Al-Mutairi NM, Ahmad S, Mokaddas E. Molecular Screening Versus Phenotypic Susceptibility Testing of Multidrug-Resistant *Mycobacterium tuberculosis* Isolates for Streptomycin and Ethambutol. *Microbial Drug Resistance* 2018; **24**: 923–931.

101 Al-Mutairi NM, Ahmad S, Mokaddas EM. Correction to: Molecular characterization of multidrug-resistant Mycobacterium tuberculosis (MDR-TB) isolates identifies local transmission of infection in Kuwait, a country with a low incidence of TB and MDR-TB. *Eur J Med Res* 2020; **25**: 14.

102 Al-Rubaye DS, Henihan G, Al-Abasly AKA *et al.* Genotypic assessment of drug-resistant tuberculosis in Baghdad and other Iraqi provinces using low-cost and low-density DNA microarrays. *Journal of Medical Microbiology* 2016; **65**: 114–122.

103 Altuwaijri TA, Alhindi GK, Al-Qattan NM, Alkharashi SK, Somily AM, Altoijry AH. Occurrence of Venous Thromboembolism in Hospitalized Patients with Tuberculosis in Saudi Arabia: A Retrospective Cohort Study. *International Journal of Mycobacteriology* 2020; **9**: 4.

104 Alyamani EJ, Marcus SA, Ramirez-Busby SM *et al.* Genomic analysis of the emergence of drug-resistant strains of Mycobacterium tuberculosis in the Middle East. *Sci Rep* 2019; **9**: 4474.

105 Al-Zarouni M, Dash N, Al Ali M, Al-Shehhi F, Panigrahi D. Tuberculosis and MDR-TB in the northern emirates of United Arab Emirates: a 5-year study. *Southeast Asian J Trop Med Public Health* 2010; **41**: 163–168.

106 Asaad AM, Alqahtani JM. Primary anti-tuberculous drugs resistance of pulmonary tuberculosis in Southwestern Saudi Arabia. *Journal of Infection and Public Health* 2012; **5**: 281–285.

107 El Mahalli AA, Al-Qahtani MF. Predictors of drug resistance in tuberculosis patients in the Eastern Province, Saudi Arabia: *Journal of the Egyptian Public Health Association* 2015; **90**: 24–28.

108 Flaifel DK, Al-Azawi IH. The Role of IL-6 Gene Polymorphism in Multidrug-Resistant Tuberculosis Patients in Iraq. *ijddt* 2020; **10**: 81–84.

109 Habous M, Elimam M, AlDabal L, Chidambaran B, AlDeesi Z. Pattern of Primary Tuberculosis Drug Resistance and Associated Risk Factors at Dubai Health Authority in Dubai. *International Journal of Mycobacteriology* 2020; **9**: 6.

110 Jaber AAS, Ibrahim B. Health-related quality of life of patients with multidrug-resistant tuberculosis in Yemen: prospective study. *Health Qual Life Outcomes* 2019; **17**: 142.

111 Jaber AAS, Ibrahim B. Evaluation of risk factors associated with drug-resistant tuberculosis in Yemen: data from centres with high drug resistance. *BMC Infect Dis* 2019; **19**: 464.

112 Kareem PA, Alsammak EGH, Abdullah YJ, Bdaiwi QM. Estimation of antibacterial activity of zinc oxide, titanium dioxide, and silver nanoparticles against multidrug-resistant bacteria isolated from clinical cases in Amara City, Iraq. *Drug Invention Today* 2019; **11**: 5.

113 M. Elhassan M, A. Hemeg H, A. Elmekki M, A. Turkistani K, A. Abdul-Aziz A. Burden of Multidrug Resistant Mycobacterium tuberculosis Among New Cases in Al-Madinah Al-Monawarah, Saudi Arabia. *IDDT* 2017; **17**: 14–23.

114 Merza MA, Farnia P, Salih AM, Masjedi MR, Velayati AA. First insight into the drug resistance pattern of Mycobacterium tuberculosis in Dohuk, Iraq: Using spoligotyping and MIRU-VNTR to characterize multidrug resistant strains. *Journal of Infection and Public Health* 2011; **4**: 41–47.

115 Mokaddas E, Ahmad S, Eldeen HS, Al-Mutairi N. Discordance between Xpert MTB/RIF Assay and Bactec MGIT 960 Culture System for Detection of Rifampin-Resistant Mycobacterium tuberculosis Isolates in a Country with a Low Tuberculosis (TB) Incidence. *J Clin Microbiol* 2015; **53**: 1351–1354.

116 Sambas MFMK, Rabbani U, Al-Gethamy MMM *et al.* Prevalence and Determinants of Multidrug-Resistant Tuberculosis in Makkah, Saudi Arabia. *IDR* 2020; **Volume 13**: 4031–4038.

117 Somily AM, Naeem T, Habib HA, Sarwar MS, Kunimoto DY, Kambal AM. Changing epidemiology of tuberculosis detected by an 8-year retrospective laboratory study in a tertiary teaching hospital in central Saudi Arabia. *Saudi Med J* 2014; **35**: 691–698.

118 Varghese B, Supply P, Allix-Béguec C *et al.* Admixed Phylogenetic Distribution of Drug Resistant Mycobacterium tuberculosis in Saudi Arabia. *PLoS ONE* 2013; **8**: e55598.

119 Varghese B, Al-Hajoj S. First Insight Into the Fluoroquinolone and Aminoglycoside Resistance of Multidrug-Resistant Mycobacterium tuberculosis in Saudi Arabia. *The American Journal of Tropical Medicine and Hygiene* 2017; **96**: 1066–1070.

120 Abulhasan YB, Abdullah AA, Shetty SA, Ramadan MA, Yousef W, Mokaddas EM. Health Care-Associated Infections in a Neurocritical Care Unit of a Developing Country. *Neurocrit Care* 2020; **32**: 836–846.

121 Ahmed SS, Shariq A, Alsalloom AA, Babikir IH, Alhomoud BN. Uropathogens and their antimicrobial resistance patterns: Relationship with urinary tract infections. *International Journal of Health Sciences* 2019; **13**: 8.

122 Al Bshabshe A, Joseph MRP, Al Hussein A, Haimour W, Hamid ME. Multidrug resistance Acinetobacter species at the intensive care unit, Aseer Central Hospital, Saudi Arabia: A one year analysis. *Asian Pacific Journal of Tropical Medicine* 2016; **9**: 903–908.

123 Al Samawi MS, Khan FY, Eldeeb Y *et al. Acinetobacter* Infections among Adult Patients in Qatar: A 2-Year Hospital-Based Study. *Canadian Journal of Infectious Diseases and Medical Microbiology* 2016; **2016**: 1–5.

124 Al-Agamy MH, Jeannot K, El-Mahdy TS *et al.* First Detection of GES-5 Carbapenemase-Producing *Acinetobacter baumannii* Isolate. *Microbial Drug Resistance* 2017; **23**: 556–562.

125 Alamri AM, Alsultan AA, Ansari MA, Alnimr AM. Biofilm-Formation in Clonally Unrelated Multidrug-Resistant Acinetobacter baumannii Isolates. *Pathogens* 2020; **9**: 630.

126 Al-Anazi KA, Abdalhamid B, Alshibani Z *et al. Acinetobacter baumannii* Septicemia in a Recipient of an Allogeneic Hematopoietic Stem Cell Transplantation. *Case Reports in Transplantation* 2012; **2012**: 1–5.

127 Al-Dabaibah N, Obeidat N, Shehabi A. Epidemiology features of Acinetobacter baumannii colonizing respiratory tracts of ICU patients. *International Arabic Journal of Antimicrobial Agents* 2012; **2**: 1–7.

128 ALfadli M, EL-sehsah EM, Ramadan MA-M. Risk factors and distribution of MDROs among patients with healthcare associated burn wound infection. *Germs* 2018; **8**: 199–206.

129 Alhaddad M, AlBarjas A, Alhammar L, Al Rashed A, Badger-Emeka L. Molecular Characterization and Antibiotic Susceptibility Pattern of Acinetobacter Baumannii Isolated in Intensive Care Unit Patients in Al-Hassa, Kingdom of Saudi Arabia. *Int J App Basic Med Res* 2018; **8**: 19.

130 Al-Hamad A, Pal T, Leskafi H *et al.* Molecular characterization of clinical and environmental carbapenem resistant Acinetobacter baumannii isolates in a hospital of the Eastern Region of Saudi Arabia. *Journal of Infection and Public Health* 2020; **13**: 632–636.

131 Alharbi A, Alshami I. In vitro Effects of Tigecycline in Combination with Other Antimicrobials against Multidrug-Resistant Acinetobacter baumannii Isolates. *Journal of Pure and Applied Microbiology* 2015; **8**: 497–502.

132 AL-Harmoosh R, Jarallah E, AL-Shamari A. Coexistence of the blaIMP and blaSIM Genes in Clinical Isolates of AcinetobacterbaumanniiIN Babylon HospitalsIraq. *International Journal of PharmTech Research* 2016; **9**: 257–264.

133 Ali HM, Salem MZM, El-Shikh MS, Megeed AA, Alogaibi YA, Talea IA. Investigation of the Virulence Factors and Molecular Characterization of the Clonal Relations of Multidrug-Resistant Acinetobacter baumannii Isolates. *Journal of AOAC INTERNATIONAL* 2017; **100**: 152–158.

134 Ali KM, Al-Jaff BMA. Source and antibiotic susceptibility of gram-negative bacteria causing superficial incisional surgical site infections. *International Journal of Surgery Open* 2021; **30**: 100318.

135 Ali M.Marie M, Gowda Krishnappa L, J. Alzahrani A, A. Mubaraki M, A. Alyousef A. A prospective evaluation of synergistic effect of sulbactam and tazobactam combination with meropenem or colistin against multidrug resistant Acinetobacter baumannii. *Bosn J of Basic Med Sci* 2015; **15**. doi:10.17305/bjbms.2015.526.

136 Aljindan R, Bukharie H, Alomar A, Abdalhamid B. Prevalence of digestive tract colonization of carbapenem-resistant Acinetobacter baumannii in hospitals in Saudi Arabia. *Journal of Medical Microbiology* 2015; **64**: 400–406.

137 AL-Kadmy I, Ali A, Salman I, Khazaal S. Molecular characterization of Acinetobacter baumannii isolated from Iraqi hospital environment. *New Microbes and New Infections* 2017; **21**: 51–57.

138 Almaghrabi MK, Joseph MRP, Assiry MM, Hamid ME. Multidrug-Resistant *Acinetobacter baumannii* : An Emerging Health Threat in Aseer Region, Kingdom of Saudi Arabia. *Canadian Journal of Infectious Diseases and Medical Microbiology* 2018; **2018**: 1–4.

139 Almangour TA, Alenazi B, Ghonem L, Alhifany AA, Aldakheel BA, Alruwaili A. Inhaled colistin for the treatment of nosocomial pneumonia due to multidrug-resistant Gram-negative bacteria: A real-life experience in tertiary care hospitals in Saudi Arabia. *Saudi Pharmaceutical Journal* 2020; **28**: 1009–1013.

140 Almutairy R, Aljrarri W, Noor A *et al.* Impact of Colistin Dosing on the Incidence of Nephrotoxicity in a Tertiary Care Hospital in Saudi Arabia. *Antibiotics* 2020; **9**: 485.

141 Al-Obeid S, Jabri L, Al-Agamy M, Al-Omari A, Shibl A. Epidemiology of extensive drug resistant *Acinetobacter baumannii* (XDRAB) at Security Forces Hospital (SFH) in Kingdom of Saudi Arabia (KSA). *Journal of Chemotherapy* 2015; **27**: 156–162.

142 Al-Ouqaili MTS, Jal'oot AS, Badawy AS. Identification of an OprD and bla IMP Gene-mediated Carbapenem Resistance in Acinetobacter baumannii and Pseudomonas aeruginosa among Patients with Wound Infections in Iraq. 2018. doi:10.13140/RG.2.2.27887.28329.

143 Al-Sweih NA, Al-Hubail MA, Rotimi VO. Emergence of Tigecycline and Colistin Resistance in *Acinetobacter* Species Isolated from Patients in Kuwait Hospitals. *Journal of Chemotherapy* 2011; **23**: 13–16.

144 Al-Sweih NA, Al-Hubail M, Rotimi VO. Three distinct clones of carbapenem-resistant Acinetobacter baumannii with high diversity of carbapenemases isolated from patients in two hospitals in Kuwait. *Journal of Infection and Public Health* 2012; **5**: 102–108.

145 Aly M, Tayeb HT, Al Johani SM *et al.* Genetic diversity of OXA-51-like genes among multidrug-resistant Acinetobacter baumannii in Riyadh, Saudi Arabia. *Eur J Clin Microbiol Infect Dis* 2014; **33**: 1223–1228.

146 Aly MM, Abu Alsoud NM, Elrobh MS, Al Johani SM, Balkhy HH. High prevalence of the PER-1 gene among carbapenem-resistant Acinetobacter baumannii in Riyadh, Saudi Arabia. *Eur J Clin Microbiol Infect Dis* 2016; **35**: 1759–1766.

147 Azeez ZF, Hatite Al-Daraghi WA. Isolation of Lytic Acinetobacter baumannii Phage vB_Acib_C_A10 from Iraq pond waters and Comparing Its Antibacterial Effect with Cefotaxime Antibiotic. *IJPQA* 2019; **10**. doi:10.25258/ijpqa.10.1.14.

148 Bakour S, Alsharapy SA, Touati A, Rolain J-M. Characterization of *Acinetobacter baumannii* Clinical Isolates Carrying *bla* $_{\text{OXA-23}}$ Carbapenemase and 16S rRNA Methylase *armA* genes in Yemen. *Microbial Drug Resistance* 2014; **20**: 604–609.

149 Batarseh A, Al-Sarhan A, Maayteh M, Al-Khatirei S, Alarmouti M. Antibiogram of multidrug resistant Acinetobacter baumannii isolated from clinical specimens at King Hussein Medical Centre, Jordan: a retrospective analysis. *Easter Mediterr Health J* 2015; **21**: 828–834.

150 Conlon JM, Ahmed E, Pal T, Sonnevend A. Potent and rapid bactericidal action of alyteserin-1c and its [E4K] analog against multidrug-resistant strains of Acinetobacter baumannii. *Peptides* 2010; **31**: 1806–1810.

151 Conlon JM, Mechkarska M, Arafat K, Attoub S, Sonnevend A. Analogues of the frog skin peptide alyteserin-2a with enhanced antimicrobial activities against Gram-negative bacteria: ALYTESERIN-2: STRUCTURE-ACTIVITY. *J Pept Sci* 2012; **18**: 270–275.

152 Conlon JM, Sonnevend A, Pál T, Vila-Farrés X. Efficacy of six frog skin-derived antimicrobial peptides against colistin-resistant strains of the Acinetobacter baumannii group. *International Journal of Antimicrobial Agents* 2012; **39**: 317–320.

153 El-Ageery SM, Abo-Shadi MA, Elgendy AM, Alghaithy AA, Kandeel AY. The Role of Health Care Workers and Environment on Transmission of Methicillin–Resistant Staphylococcus aureus among Patients in a Medical Intensive Care Unit in a Saudi Hospital. *Appl Microbiol*; : 9.

154 Ghaima K, Saadedin S, Jassim K. Prevalence of BlaOXA like Carbapenemase Genes in Multidrug Resistant Acinetobacter baumannii Isolated from burns and Wounds in Baghdad Hospitals. *Research Journal of Pharmaceutical, Biological and Chemical Sciences* 2016; **7**: 1347.

155 Gowda KL, Marie MAM, Al-Sheikh YA *et al.* A 6-year surveillance of antimicrobial resistance patterns of *Acinetobacter baumannii* bacteremia isolates from a tertiary care hospital in Saudi Arabia during 2005–2010. *Libyan Journal of Medicine* 2014; **9**: 24039.

156 Hoang V-T, Dao T-L, Ly TDA *et al.* Acquisition of multidrug-resistant bacteria and encoding genes among French pilgrims during the 2017 and 2018 Hajj. *Eur J Clin Microbiol Infect Dis* 2021; **40**: 1199–1207.

157 Jamal W, Al Roomi E, AbdulAziz LR, Rotimi VO. Evaluation of Curetis Unyvero, a Multiplex PCR-Based Testing System, for Rapid Detection of Bacteria and Antibiotic Resistance and Impact of the Assay on Management of Severe Nosocomial Pneumonia. *Journal of Clinical Microbiology* 2014; **52**: 2487–2492.

158 Kareem SM, Al-Kadmy IMS, Al-Kaabi MH, Aziz SN, Ahmad M. Acinetobacter baumannii virulence is enhanced by the combined presence of virulence factors genes phospholipase C (plcN) and elastase (lasB). *Microbial Pathogenesis* 2017; **110**: 568–572.

159 Kusradze I, Diene SM, Goderdzishvili M, Rolain J-M. Molecular detection of OXA carbapenemase genes in multidrug-resistant Acinetobacter baumannii isolates from Iraq and Georgia. *International Journal of Antimicrobial Agents* 2011; **38**: 164–168.

160 Lopes BS, Al-Agamy MH, Ismail MA *et al.* The transferability of blaOXA-23 gene in multidrug-resistant Acinetobacter baumannii isolates from Saudi Arabia and Egypt. *International Journal of Medical Microbiology* 2015; **305**: 581–588.

161 Mahdi L, Mahdi N, Al-kakei S *et al.* Treatment strategy by lactoperoxidase and lactoferrin combination: Immunomodulatory and antibacterial activity against multidrug-resistant Acinetobacter baumannii. *Microbial Pathogenesis* 2018; **114**: 147–152.

162 Mazi W, Alshammari F, Yu J, Saeed A. A descriptive analysis of PVL-positive multidrug-resistant Staphylococcus aureus in hospital-associated infections in Saudi Arabia. *Bioinformation* 2020; **16**: 586–593.

163 Mechkarska M, Prajeep M, Radosavljevic GD *et al.* An analog of the host-defense peptide hymenochirin-1B with potent broad-spectrum activity against multidrug-resistant bacteria and immunomodulatory properties. *Peptides* 2013; **50**: 153–159.

164 Muslim SN, Al-Kadmy IMS, Auda IG, Mohammed Ali AN, Al-Jubori SS. A Novel Genetic Determination of a Lectin Gene in Iraqi Acinetobacter baumannii Isolates and Use of Purified Lectin as an Antibiofilm Agent. *Journal of AOAC INTERNATIONAL* 2018; **101**: 1623–1630.

165 Nasser K, Mustafa AS, Khan MW *et al.* Draft Genome Sequences of Six Multidrug-Resistant Clinical Strains of Acinetobacter baumannii, Isolated at Two Major Hospitals in Kuwait. *Genome Announc* 2018; **6**. doi:10.1128/genomeA.00264-18.

166 Obeidat N, Jawdat F, Al-Bakri AG, Shehabi AA. Major biologic characteristics of Acinetobacter baumannii isolates from hospital environmental and patients' respiratory tract sources. *American Journal of Infection Control* 2014; **42**: 401–404.

167 Qasim ZJ, Kadhim HS, Abdulamir AS. Identification of Antibiotic Resistance Genes in Multi-Drug Resistant Acinetobacter Baumannii Clinical Isolates of Iraqi Patients (Zq Strains), Using Whole-Genome Sequencing. *ijpqa* 2019; **10**: 670–680.

168 Rabaan AA, Saunar JV, Bazzi AM, Raslan WF, Taylor DR, Al-Tawfiq JA. Epidemiology and detection of acinetobacter using conventional culture and in-house developed PCR based methods. *Journal of Infection and Public Health* 2017; **10**: 124–128.

169 Radhi SH, Al-Charrakh AH. Occurrence of MBLs and Carbapenemases among MDR and XDR *Acinetobacter baumannii* Isolated from Hospitals in Iraq. *Ind Jour of Publ Health Rese & Develop* 2019; **10**: 668.

170 Ridha D, Ali M, Jassim K. Occurrence of Metallo-β-lactamase Genes among Acinetobacter baumannii Isolated from Different Clinical Samples. *J Pure Appl Microbiol* 2019; **13**: 1111–1119.

171 Saeed NK, Kambal AM, El-Khizzi NA. Antimicrobial-resistant bacteria in a general intensive care unit in Saudi Arabia. *Saudi Med J* 2010; **31**: 1341–1349.

172 Salahuddin N, Amer L, Joseph M, El Hazmi A, Hawa H, Maghrabi K. Determinants of Deescalation Failure in Critically Ill Patients with Sepsis: A Prospective Cohort Study. *Critical Care Research and Practice* 2016; **2016**: 1–7.

173 Samrah S, Bashtawi Y, Hayajneh W, Almomani B, Momany S, Khader Y. Impact of colistin-initiation delay on mortality of ventilator-associated pneumonia caused by A. baumannii. *J Infect Dev Ctries* 2016; **10**: 1129–1134.

174 Senok A, Garaween G, Raji A, Khubnani H, Kim Sing G, Shibl A. Genetic relatedness of clinical and environmental Acinetobacter baumanii isolates from an intensive care unit outbreak. *J Infect Dev Ctries* 2015; **9**: 665–669.

175 Shah MW, Yasir M, Farman M *et al.* Antimicrobial Susceptibility and Molecular Characterization of Clinical Strains of *Acinetobacter baumannii* in Western Saudi Arabia. *Microbial Drug Resistance* 2019; **25**: 1297–1305.

176 Somily AM. Comparison of E-test and disc diffusion methods for the in vitro evaluation of the antimicrobial activity of colistin in multi-drug resistant Gram-negative Bacilli. 2010; : 5.

177 Somily AM, Absar MM, Arshad MZ *et al.* Antimicrobial susceptibility patterns of multidrug-resistant Pseudomonas aeruginosa and Acinetobacter baumannii against carbapenems, colistin, and tigecycline. *Saudi Med J* 2012; **33**: 750–755.

178 Vijayakumar R, Sandle T, Al-Aboody MS *et al.* Distribution of biocide resistant genes and biocides susceptibility in multidrug-resistant Klebsiella pneumoniae, Pseudomonas aeruginosa and Acinetobacter baumannii — A first report from the Kingdom of Saudi Arabia. *Journal of Infection and Public Health* 2018; **11**: 812–816.

179 Wahaab ITA, Almaroof SQM, Yaseen ZT. Causative Microorganisms and Antibiotics Susceptibility in Neonatal Sepsis at Neonatal Intensive Care Unit: A Longitudinal Study from Diyala Governorate in Iraq. *IJFMT* 2021. doi:10.37506/ijfmt.v15i1.13382.

180 Wibberg D, Salto IP, Eikmeyer FG *et al.* Complete Genome Sequencing of Acinetobacter baumannii Strain K50 Discloses the Large Conjugative Plasmid pK50a Encoding Carbapenemase OXA-23 and Extended-Spectrum β-Lactamase GES-11. *Antimicrob Agents Chemother* 2018; **62**. doi:10.1128/AAC.00212-18.

181 Yasir M, Shah MW, Jiman-Fatani AA *et al.* Draft genome sequence of a clinical Acinetobacter baumannii isolate of new sequence type ST1688 from Saudi Arabia. *Journal of Global Antimicrobial Resistance* 2019; **18**: 151–152.

182 Zowawi HM, Sartor AL, Sidjabat HE *et al.* Molecular Epidemiology of Carbapenem-Resistant Acinetobacter baumannii Isolates in the Gulf Cooperation Council States: Dominance of OXA-23-Type Producers. *J Clin Microbiol* 2015; **53**: 896–903.

183 Al-Agamy MH, El-Mahdy TS, Radwan HH, Poirel L. Cooccurrence of NDM-1, ESBL, RmtC, AAC(6′)-Ib, and QnrB in Clonally Related *Klebsiella pneumoniae* Isolates Together with Coexistence of CMY-4 and AAC(6′)-Ib in *Enterobacter cloacae* Isolates from Saudi Arabia. *BioMed Research International* 2019; **2019**: 1–7.

184 Alatoom A, Sartawi M, Lawlor K *et al.* Persistent candidemia despite appropriate fungal therapy: First case of Candida auris from the United Arab Emirates. *International Journal of Infectious Diseases* 2018; **70**: 36–37.

185 Al-Baloushi AE, Pál T, Ghazawi A, Sonnevend A. Genetic support of carbapenemases in double carbapenemase producer Klebsiella pneumoniae isolated in the Arabian Peninsula. *AMicr* 2018; **65**: 135–150.

186 Alghoribi MF, Binkhamis K, Alswaji AA *et al.* Genomic analysis of the first KPC-producing Klebsiella pneumoniae isolated from a patient in Riyadh: A new public health concern in Saudi Arabia. *Journal of Infection and Public Health* 2020; **13**: 647–650.

187 Algowaihi R, Ashgar S, Sirag B, Shalam S, Nassir A, Ahmed A. Draft Genome Sequence of a Multidrug-Resistant Klebsiella pneumoniae Strain Isolated from King Abdullah Medical City, Makkah, Saudi Arabia. *Genome Announc* 2016; **4**. doi:10.1128/genomeA.00375-16.

188 AL-Khikani FHO, Abadi RM, Ayit AS. Emerging Carbapenemase Klebsiella oxytoca with Multidrug Resistance Implicated in Urinary Tract Infection. 2020; **4**: 4.

189 AL-Muqdadi BMJ, AL-Saadi BQH. DETECTION OF ARMA GENE, KPC ENZYME AND MOLECULAR TYPING OF K. PNEUMONIAE CLINICAL ISOLATE FROM PUBLIC HOSPITALS IN BAGHDAD CITY, IRAQ. ; : 6.

190 Al-Qahtani AA, Al-Agamy MH, Ali MS, Al-Ahdal MN, Aljohi MA, Shibl AM. Characterization of extended-spectrum beta-lactamase-producing *Klebsiella pneumoniae* from Riyadh, Saudi Arabia. *Journal of Chemotherapy* 2014; **26**: 139–145.

191 Alraddadi BM, Saeedi M, Qutub M, Alshukairi A, Hassanien A, Wali G. Efficacy of ceftazidime-avibactam in the treatment of infections due to Carbapenem-resistant Enterobacteriaceae. *BMC Infect Dis* 2019; **19**: 772.

192 Alsanie WF. Molecular diversity and profile analysis of virulence-associated genes in some Klebsiella pneumoniae isolates. *Practical Laboratory Medicine* 2020; **19**: e00152.

193 Alyousef AA, Khazaal SS, Ali ANM, Hussein NH, Hussein SMA. Detection of prevalent mechanism of extended spectrum β-lactamases, metallo β-lactamases, and AmpC β lactamases-producing Klebsiella pneumoniae in the tertiary care hospital. *Reviews in Medical Microbiology* 2017; **28**: 133–139.

194 Salman and Ghaima. 2018; **15**: 11.

195 Bindayna K, Khanfar HS, Senok AC, Botta GA. Predominance of CTX-M genotype among extended spectrum beta lactamase isolates in a tertiary hospital in Saudi Arabia. *Saudi Med J* 2010; **31**: 859–863.

196 El Nekidy WS, Mooty MY, Attallah N, Cardona L, Bonilla MF, Ghazi IM. Successful treatment of multidrug resistant Klebsiella pneumoniae using dual carbapenem regimen in immunocompromised patient. *IDCases* 2017; **9**: 53–55.

197 Hassan MI, Alkharsah KR, Alzahrani AJ, Obeid OE, Khamis AH, Diab A. Detection of extended spectrum beta-lactamases-producing isolates and effect of AmpC overlapping. *J Infect Dev Ctries* 2013; **7**: 618–629.

198 Khan FY, Abukhattab M, AbuKamar M, Anand D. Adult K lebsiella pneumoniae meningitis in Qatar: clinical pattern of ten cases. *Asian Pacific Journal of Tropical Biomedicine* 2014; **4**: 669–672.

199 Lagha R, Ben Abdallah F, ALKhammash AAH *et al.* Molecular characterization of multidrug resistant Klebsiella pneumoniae clinical isolates recovered from King Abdulaziz Specialist Hospital at Taif City, Saudi Arabia. *Journal of Infection and Public Health* 2021; **14**: 143–151.

200 Poirel L, Al Maskari Z, Al Rashdi F, Bernabeu S, Nordmann P. NDM-1-producing Klebsiella pneumoniae isolated in the Sultanate of Oman. *Journal of Antimicrobial Chemotherapy* 2011; **66**: 304–306.

201 Saaid Tuwaij NS, Al-khilkhali HJB, Mohsen HM. Prevalence of SUL(1,2), GYR(A, B) and OXA genes among multidrug resistance Klebsiella pneumoniae isolates recovered from women suffering urinary tract infection. *ijrps* 2020; **11**: 2424–2432.

202 Salman R, Ghaima K. Prevalence of ESBL genes in ESBL producing Klebsiella pneumoniae isolated from patients with urinary tract infections in Baghdad, Iraq. *BIOSCIENCE RESEARCH* 2018; **15**: 2049–2059.

203 Shibl A, Al-Agamy M, Memish Z, Senok A, Khader SA, Assiri A. The emergence of OXA-48- and NDM-1-positive Klebsiella pneumoniae in Riyadh, Saudi Arabia. *International Journal of Infectious Diseases* 2013; **17**: e1130–e1133.

204 Shlash AAA, Tuwaij NSS. MOLECULAR DISSEMINATION OF AMBLER CLASS A AND C β- LACTAMASE GENES AMONG CEFTRIAXONE RESISTANT KLEBSIELLA PNEUMONIAE INFECTION IN NAJAF CITY, IRAQ. ; : 13.

205 Vali L, Dashti AA, Jadaon MM, El-Shazly S. The emergence of plasmid mediated quinolone resistance qnrA2 in extended spectrum β-lactamase producing Klebsiella pneumoniae in the Middle East. *DARU J Pharm Sci* 2015; **23**: 34.

206 Zaman T uz, Alrodayyan M, Albladi M *et al.* Clonal diversity and genetic profiling of antibiotic resistance among multidrug/carbapenem-resistant Klebsiella pneumoniae isolates from a tertiary care hospital in Saudi Arabia. *BMC Infect Dis* 2018; **18**: 205.

207 uz Zaman T, Aldrees M, Al Johani SM, Alrodayyan M, Aldughashem FA, Balkhy HH. Multi-drug carbapenem-resistant Klebsiella pneumoniae infection carrying the OXA-48 gene and showing variations in outer membrane protein 36 causing an outbreak in a tertiary care hospital in Riyadh, Saudi Arabia. *International Journal of Infectious Diseases* 2014; **28**: 186–192.

208 AbdulWahab A, Taj-Aldeen SJ, Ibrahim EB *et al.* Genetic relatedness and host specificity of Pseudomonas aeruginosa isolates from cystic fibrosis and non-cystic fibrosis patients. *IDR* 2014; : 309.

209 AbdulWahab A, Zahraldin K, Sid Ahmed M *et al.* The emergence of multidrug-resistant Pseudomonas aeruginosa in cystic fibrosis patients on inhaled antibiotics. *Lung India* 2017; **34**: 527.

210 Ahmad I, Irfan S, Abohashrh M *et al.* Inhibitory Effect of Nepeta deflersiana on Climax Bacterial Community Isolated from the Oral Plaque of Patients with Periodontal Disease. *Molecules* 2021; **26**: 202.

211 Alamri AM, Alfifi S, Aljehani Y, Alnimr A. Whole Genome Sequencing of Ceftolozane-Tazobactam and Ceftazidime-Avibactam Resistant Pseudomonas aeruginosa Isolated from a Blood Stream Infection Reveals VEB and Chromosomal Metallo-Beta Lactamases as Genetic Determinants: A Case Report. *IDR* 2020; **Volume 13**: 4215–4222.

212 Al-Delaimi MS, Yacoob Aldosky HY. Amending the Efficiency of Antimicrobials against Multidrug-Resistant Pseudomonas aeruginosa by Low-Frequency Magnetic Fields. *Bull Exp Biol Med* 2020; **170**: 35–39.

213 Alhamdani RJM, Al-Luaibi YYY. DETECTION OF EXOA, NAN1 GENES, THE BIOFILM PRODUCTION WITH THE EFFECT OF OYSTER SHELL AND TWO PLANT EXTRACTS ON PSEUDOMONAS AEROGINOSA ISOLATED FROM BURN' PATIENT AND THEIR SURROUNDING ENVIRONMENT. *Systematic Reviews in Pharmacy* 2020; **11**: 11.

214 Alhussain FA, Yenugadhati N, Al Eidan FA, Al Johani S, Badri M. Risk factors, antimicrobial susceptibility pattern and patient outcomes of Pseudomonas aeruginosa infection: A matched case-control study. *Journal of Infection and Public Health* 2021; **14**: 152–157.

215 Al-Zahrani IA, Al-Ahmadi BM. Dissemination of VIM-producing *Pseudomonas aeruginosa* associated with high-risk clone ST654 in a tertiary and quaternary hospital in Makkah, Saudi Arabia. *Journal of Chemotherapy* 2021; **33**: 12–20.

216 Asghar A. Antimicrobial susceptibility and metallo-β-lactamase production among Pseudomonas aeruginosa isolated from Makkah hospitals. *Pakistan Journal of Medical Sciences* 2012; **28**: 781–786.

217 Behbahani MR, Keshavarzi A, Pirbonyeh N, Javanmardi F, Khoob F, Emami A. Plasmid--related β-lactamase genes in Pseudomonas aeruginosa isolates: a molecular study in burn patients. *Journal of Medical Microbiology*; : 7.

218 Bosaeed M, Ahmad A, Alali A *et al.* Experience With Ceftolozane-Tazobactam for the Treatment of Serious *Pseudomonas aeruginosa* Infections in Saudi Tertiary Care Center. *Infect Dis (Auckl)* 2020; **13**: 117863372090597.

219 Bourghli A, Boissiere L, Obeid I. Thoracic Kyphotic Deformity Secondary to Old *Pseudomonas aeruginosa* Spondylodiscitis in an Immunocompromised Patient With Persistent Infection Foci—A Case Report. *Int J Spine Surg* 2019; **13**: 392–398.

220 Hassan S, Najati A, Abass K. Isolation and identification of multi-drug resistant "pseudomonas aeruginosa" from burn wound infection in Kirkuk City, Iraq. *Eurasian Journal of Biosciences* 2019; **13**: 1045–1050.

221 Hassan SA, Shobrak MY. Prevalence and Antimicrobial Resistance Characteristics of Gram-negative Bacteria Associated with Wild Animals Presenting at Live Animal Market, Taif, Western Saudi Arabia. *ANTIMICROBIAL RESISTANCE* 2014; : 11.

222 Jaaffar AI. Detection of blaIMP gene among Pseudomonas aeruginosa isolated from different clinical samples. *Drug Invention Today* 2019; **12**: 5.

223 Khan R, Al-Dorzi HM, Tamim HM *et al.* The impact of onset time on the isolated pathogens and outcomes in ventilator associated pneumonia. *Journal of Infection and Public Health* 2016; **9**: 161–171.

224 Khan MA, Faiz A. Antimicrobial resistance patterns of *Pseudomonas aeruginosa* in tertiary care hospitals of Makkah and Jeddah. *Annals of Saudi Medicine* 2016; **36**: 23–28.

225 Mahdi LH, Jabbar HS, Auda IG. Antibacterial immunomodulatory and antibiofilm triple effect of Salivaricin LHM against Pseudomonas aeruginosa urinary tract infection model. *International Journal of Biological Macromolecules* 2019; **134**: 1132–1144.

226 Nasser M, Gayen S, Kharat AS. Prevalence of β-lactamase and antibiotic-resistant Pseudomonas aeruginosa in the Arab region. *Journal of Global Antimicrobial Resistance* 2020; **22**: 152–160.

227 Nasser M, Kharat AS. Phenotypic Demonstration of ß-lactamase (ESßLs, MßLs, and Amp-C) among MDR Pseudomonas aeruginosa isolates obtained From Burn wound infected in Yemen. *Jabb* 2019; **7**: 31–34.

228 Sid Ahmed MA, Khan FA, Sultan AA *et al.* β-lactamase-mediated resistance in MDR-Pseudomonas aeruginosa from Qatar. *Antimicrob Resist Infect Control* 2020; **9**: 170.

229 Tarazi YH, Abu-Basha E, Ismail ZB, Al-Jawasreh SI. Antimicrobial susceptibility of multidrug-resistant Pseudomonas aeruginosa isolated from drinking water and hospitalized patients in Jordan. *Acta Tropica* 2021; **217**: 105859.

230 Zikri A, El Masri K. Use of Ceftolozane/Tazobactam for the Treatment of Multidrug-Resistant Pseudomonas aeruginosa Pneumonia in a Pediatric Patient with Combined Immunodeficiency (CID): A Case Report from a Tertiary Hospital in Saudi Arabia. *Antibiotics* 2019; **8**: 67.

231 Abulreesh HH, Organji SR, Osman GEH, Elbanna K, Almalki MHK, Ahmad I. Prevalence of antibiotic resistance and virulence factors encoding genes in clinical Staphylococcus aureus isolates in Saudi Arabia. *Clinical Epidemiology and Global Health* 2017; **5**: 196–202.

232 Abulreesh HH, Organji SR. The Prevalence of Multidrug-resistant Staphylococci in Food and the Environment of Makkah, Saudi Arabia. *Research J of Microbiology* 2011; **6**: 510–523.

233 Al Zebary MK, Yousif SY, Assafi MS. The Prevalence, Molecular Characterization and Antimicrobial Susceptibility of S. aureus Isolated from Impetigo Cases in Duhok, Iraq. *TODJ* 2017; **11**: 22–29.

234 AlFouzan W, Al-Haddad A, Udo E, Mathew B, Dhar R. Frequency and Clinical Association of Panton-Valentine Leukocidin-Positive ***Staphylococcus aureus*** Isolates: A Study from Kuwait. *Med Princ Pract* 2013; **22**: 245–249.

235 Alghizzi MJ, Alansari M, Shami A. The Prevalence of Staphylococcus aureus and Methicillin Resistant Staphylococcus aureus in Processed Food Samples in Riyadh, Saudi Arabia. *J Pure Appl Microbiol* 2021; **15**: 91–99.

236 Al-Zoubi MS, Al-Tayyar IA, Hussein E, Jabali AA, Khudairat S. Antimicrobial susceptibility pattern of Staphylococcus aureus isolated from clinical specimens in Northern area of Jordan. ; : 8.

237 Babakir-Mina M, Othman N, Najmuldeen HH *et al.* Antibiotic susceptibility of vancomyin and nitrofurantoin in Staphylococcus aureus isolated from burnt patients in Sulaimaniyah, Iraqi Kurdistan. 2012; : 8.

238 Hamid, ME. Prevalence of Bacterial Pathogens in Aseer Region, Kingdom of Saudi Arabia: Emphasis on Antimicrobial Susceptibility of Staphylococcus aureus. *OMJ* 2011; **26**: 368–369.

239 Ismail ZB. Molecular characteristics, antibiogram and prevalence of multi-drug resistant Staphylococcus aureus (MDRSA) isolated from milk obtained from culled dairy cows and from cows with acute clinical mastitis. *Asian Pacific Journal of Tropical Biomedicine* 2017; **7**: 694–697.

240 Kanaan MHG. Antibacterial effect of ozonated water against methicillin-resistant Staphylococcus aureus contaminating chicken meat in Wasit Province, Iraq. *Vet World* 2018; **11**: 1445–1453.

241 Kanaan MHG, Al-Isawi AJO. PREVALENCE OF METHICILLIN OR MULTIPLE DRUG-RESISTANT STAPHYLOCOCCUS AUREUS IN CATTLE MEAT MARKETED IN WASIT PROVINCE. 2019; **19**: 495–502.

242 Obaidat MM, Bani Salman AE, Lafi SQ. Prevalence of Staphylococcus aureus in Imported Fish and Correlations between Antibiotic Resistance and Enterotoxigenicity. *Journal of Food Protection* 2015; **78**: 1999–2005.

243 Vellappally S, Divakar DD, Al Kheraif AA *et al.* Occurrence of vancomycin-resistant Staphylococcus aureus in the oral cavity of patients with dental caries. *AMicr* 2017; **64**: 343–351.

244 Abdalla NM, Osman AA, Haimour WO *et al.* Antimicrobial Susceptibility Pattern in Nosocomial Infections Caused by Acinetobacter Species in Asir Region, Saudi Arabia. *Pakistan J of Biological Sciences* 2013; **16**: 275–280.

245 Al-lawama M, Aljbour H, Tanash A, Badran E. Intravenous Colistin in the treatment of multidrug-resistant Acinetobacter in neonates. *Ann Clin Microbiol Antimicrob* 2016; **15**: 8.

246 Balkhy HH, Bawazeer MS, Kattan RF *et al.* Epidemiology of Acinetobacter spp.-associated healthcare infections and colonization among children at a tertiary-care hospital in Saud Arabia: a 6-year retrospective cohort study. *Eur J Clin Microbiol Infect Dis* 2012; **31**: 2645–2651.

247 Somily AM, Al-Khattaf AS, Kambal AM. Antimicrobial activity of tigecycline against bacterial isolates from intensive care units in a teaching hospital in Central Saudi Arabia. *Saudi Med J* 2010; **31**: 18–24.

248 Al Mayahi Z, Kamel S, Amer H, Beatty M. Outbreak of colistin-resistant organisms at a tertiary hospital in Riyadh, Saudi Arabia, 2016. *Pan Afr Med J* 2019; **34**. doi:10.11604/pamj.2019.34.162.19998.

249 Al-Kharousi ZS, Guizani N, Al-Sadi AM, Al-Bulushi IM. Antibiotic Resistance of Enterobacteriaceae Isolated from Fresh Fruits and Vegetables and Characterization of their AmpC β-Lactamases. *Journal of Food Protection* 2019; **82**: 1857–1863.

250 Alkofide H, Alhammad AM, Alruwaili A *et al.* Multidrug-Resistant and Extensively Drug-Resistant Enterobacteriaceae: Prevalence, Treatments, and Outcomes – A Retrospective Cohort Study. *IDR* 2020; **Volume 13**: 4653–4662.

251 Jamal WY, Albert MJ, Khodakhast F, Poirel L, Rotimi VO. Emergence of New Sequence Type OXA-48 Carbapenemase-Producing *Enterobacteriaceae* in Kuwait. *Microbial Drug Resistance* 2015; **21**: 329–334.

252 Jamal WY, Albert MJ, Rotimi VO. High Prevalence of New Delhi Metallo-β-Lactamase-1 (NDM-1) Producers among Carbapenem-Resistant Enterobacteriaceae in Kuwait. *PLoS ONE* 2016; **11**: e0152638.

253 Khan U, Huerga H, Khan AJ *et al.* The endTB observational study protocol: treatment of MDR-TB with bedaquiline or delamanid containing regimens. *BMC Infect Dis* 2019; **19**: 733.

254 Al-Mayahi FSA, Ali RH. PRELIMINARY STUDY OF EMERGENCE MDR OF PROVIDENCIA SPP. ISOLATES PRODUCING ESBL, AmpC AND MBL AMONG PATIENTS WITH RTI AND IN WASTEWATER IN AL-DIWANIYA CITY, IRAQ. *Biochem Cell Arch* 2018; **18**: 12.

255 Ayyal Al-Gburi NM. Isolation and Molecular Identification and Antimicrobial Susceptibility of Providencia spp. from Raw Cow's Milk in Baghdad, Iraq. *Veterinary Medicine International* 2020; **2020**: 1–6.

256 Hassan SA, Al-Naami MY. Prevalence and Antimicrobial Resistance Characteristics of Gram-negative Bacteria Associated with Wild Animals Presenting at Live Animal Market, Taif, Western Saudi Arabia. *J PURE APPL MICROBIO* 2014; **8**.https://microbiologyjournal.org/archive_mg/jmabsread.php?snoid=2292&month=&year=.

257 Al-Lahham A, Qayyas JA. The Impact of the 7-Valent Pneumococcal Conjugate Vaccine on Nasopharyngeal Carriage of Streptococcus pneumoniae in Infants of Ajlun Governorate in Jordan. 2018; **11**: 8.

258 Al-Mazrou KA, Shibl AM, Kandeil W, Pirçon J-Y, Marano C. A prospective, observational, epidemiological evaluation of the aetiology and antimicrobial susceptibility of acute otitis media in Saudi children younger than 5 years of age. *JEGH* 2014; **4**: 231.

259 Almazrou Y, Shibl AM, Alkhlaif R *et al.* Epidemiology of invasive pneumococcal disease in Saudi Arabian children younger than 5 years of age. *JEGH* 2015; **6**: 95.

260 Alnimr AM, Farhat M. Phenotypic and molecular study of pneumococci causing respiratory tract infections: A 3-year prospective cohort. *SMJ* 2017; **38**: 350–358.

261 Al-Sa'ady AT, Hussein FH. Nanomedical Applications of Titanium Dioxide Nanoparticles as Antibacterial Agent against Multi-Drug Resistant Streptococcus Pneumoniae. *Systematic Reviews in Pharmacy* 2020; **11**: 11.

262 Elshafie S, Taj-Aldeen SJ. Emerging resistant serotypes of invasive Streptococcus pneumoniae. *Infect Drug Resist* 2016; **9**: 153–160.

263 Sallam M. Trends in Antimicrobial Drug Resistance of Streptococcus pneumoniae Isolates at Jordan University Hospital (2000–2018). *Antibiotics* 2019; **8**: 41.

264 Shin J, Baek JY, Kim SH, Song J-H, Ko KS. Predominance of ST320 among Streptococcus pneumoniae serotype 19A isolates from 10 Asian countries. *Journal of Antimicrobial Chemotherapy* 2011; **66**: 1001–1004.

265 Taj-Aldeen SJ, Shamseldin Elshafie S. Emerging resistant serotypes of invasive Streptococcus pneumoniae. *IDR* 2016; **Volume 9**: 153–160.

266 Al Baidani A, Elshoni W, Shawa T. Antibiotic susceptibility pattern of methicillin-resistant Staphylococcus aureus in three hospitals at Hodeidah city, Yemen. *Global Journal of Pharmacology* 2011; **55**: 105–111.

267 Albarrag A, Shami A, Almutairi A, Alsudairi S, Aldakeel S, Al-Amodi A. Prevalence and Molecular Genetics of Methicillin-Resistant *Staphylococcus aureus* Colonization in Nursing Homes in Saudi Arabia. *Canadian Journal of Infectious Diseases and Medical Microbiology* 2020; **2020**: 1–6.

268 Alfouzan W, Udo EE, Modhaffer A, Alosaimi A. Molecular Characterization of Methicillin- Resistant Staphylococcus aureus in a Tertiary Care hospital in Kuwait. *Sci Rep* 2019; **9**: 18527.

269 Alfouzan W, Ahmad S, Dhar R *et al.* Molecular Epidemiology of Candida Auris Outbreak in a Major Secondary-Care Hospital in Kuwait. *JoF* 2020; **6**: 307.

270 Alhussaini MS. Methicillin-resistant Staphylococcus aureus Nasal Carriage Among Patients Admitted at Shaqra General Hospital in Saudi Arabia. *Pakistan J of Biological Sciences* 2016; **19**: 233–238.

271 Alkharsah KR, Rehman S, Alkhamis F, Alnimr A, Diab A, Al-Ali AK. Comparative and molecular analysis of MRSA isolates from infection sites and carrier colonization sites. *Ann Clin Microbiol Antimicrob* 2018; **17**: 7.

272 Aziz ZS, Hassan MA. Phenotypic and Molecular Study of *mecA* Gene in MRSA Isolated from Clinical Cases in Misan Province/Iraq. *Ind Jour of Publ Health Rese & Develop* 2019; **10**: 553.

273 Helmi NR, Zaman RM, Aly MM. PREVALENCE OF GRAM-POSITIVE BACTERIA IN JEDDAH, KINGDOM OF SAUDI ARABIA: STUDY OF ANTIMICROBIAL RESISTANCE PATTERNS AND MOLECULAR TYPING. 2013; : 15.

274 Obaidat MM, Bani Salman AE, Roess AA. High prevalence and antimicrobial resistance of mecA Staphylococcus aureus in dairy cattle, sheep, and goat bulk tank milk in Jordan. *Trop Anim Health Prod* 2018; **50**: 405–412.

275 Udo EE, Boswihi SS. Antibiotic Resistance Trends in Methicillin-Resistant ***Staphylococcus aureus*** Isolated in Kuwait Hospitals: 2011-2015. *Med Princ Pract* 2017; **26**: 485–490.

276 Hala S, Antony CP, Alshehri M *et al.* First report of Klebsiella quasipneumoniae harboring blaKPC-2 in Saudi Arabia. *Antimicrob Resist Infect Control* 2019; **8**: 203.

277 Ejaz H, Alzahrani B, Hamad MFS *et al.* Molecular Analysis of the Antibiotic Resistant NDM-1 Gene in Clinical Isolates of Enterobacteriaceae. *Clin Lab* 2020; **66**. doi:10.7754/Clin.Lab.2019.190727.

278 Abdalhamid B, Almaghrabi R, Althawadi S, Omrani A. First report of Candida auris infections from Saudi Arabia. *Journal of Infection and Public Health* 2018; **11**: 598–599.

279 Ahmad S, Khan Z, Al-Sweih N, Alfouzan W, Joseph L. *Candida auris* in various hospitals across Kuwait and their susceptibility and molecular basis of resistance to antifungal drugs. *Mycoses* 2020; **63**: 104–112.

280 AlJindan R, AlEraky DM, Mahmoud N *et al.* Drug Resistance-Associated Mutations in ERG11 of Multidrug-Resistant Candida auris in a Tertiary Care Hospital of Eastern Saudi Arabia. *J Fungi (Basel)* 2020; **7**: 18.

281 Al-Jindan R, Al-Eraky D. Two Cases of the Emerging Candida auris in a university hospital from Saudi Arabia. *Saudi J Med Med Sci* 2021; **9**: 71.

282 Almaghrabi RS, Albalawi R, Mutabagani M *et al.* Molecular characterisation and clinical outcomes of *Candida auris* infection: Single-centre experience in Saudi Arabia. *Mycoses* 2020; **63**: 452–460.

283 Emara M, Ahmad S, Khan Z *et al. Candida auris* Candidemia in Kuwait, 2014. *Emerg Infect Dis* 2015; **21**: 1091–1092.

284 Khan Z, Ahmad S, Benwan K *et al.* Invasive Candida auris infections in Kuwait hospitals: epidemiology, antifungal treatment and outcome. *Infection* 2018; **46**: 641–650.

285 Hassan MM, Belal E-SB. Antibiotic resistance and virulence genes in enterococcus strains isolated from different hospitals in Saudi Arabia. *Biotechnology & Biotechnological Equipment* 2016; **30**: 726–732.

286 Salem-Bekhit M, Moussa I, Muharram M, Alanazy F, Hefni H. Prevalence and antimicrobial resistance pattern of multidrug-resistant enterococci isolated from clinical specimens. *Indian Journal of Medical Microbiology* 2012; **30**: 44–51.

287 Dibby H, Shlash R. The Problem of Multidrug Resistance Bacterial Strains in Daily Clinical Practice in Dealing with Typhoid Fever in Mid-Euphrates Region of Iraq: A Cross Sectional Study. *Indian Journal of Forensic Medicine & Toxicology* 2020; **14**: 626–630.

288 El-Tayeb MA, Ibrahim ASS, Al-Salamah AA, Almaary KS, Elbadawi YB. Prevalence, serotyping and antimicrobials resistance mechanism of Salmonella enterica isolated from clinical and environmental samples in Saudi Arabia. *Brazilian Journal of Microbiology* 2017; **48**: 499–508.

289 Harb A, O'Dea M, Hanan ZK, Abraham S, Habib I. Prevalence, risk factors and antimicrobial resistance of *Salmonella* diarrhoeal infection among children in Thi-Qar Governorate, Iraq. *Epidemiol Infect* 2017; **145**: 3486–3496.

290 Zahraa A Sahan, Saad L Hamed. Molecular Detection of Extended-Spectrum β-Lactamases- Producer Serratia marcescens Causing Neonatal Sepsis in Iraq. *ijrps* 2020; **11**: 5803–5808.

291 Abdel-Haleem AM, Rchiad Z, Khan BK *et al.* Genome Sequence of a Multidrug-Resistant Strain of Stenotrophomonas maltophilia with Carbapenem Resistance, Isolated from King Abdullah Medical City, Makkah, Saudi Arabia. *Genome Announc* 2015; **3**. doi:10.1128/genomeA.01166-15.

292 El-Kersh TA, Marie MA, Al-Sheikh YA, Al-Agamy MH, Al-Bloushy AA. Prevalence and risk factors of early fecal carriage of *Enterococcus faecalis* and *Staphylococcus spp* and their antimicrobial resistant patterns among healthy neonates born in a hospital setting in central Saudi Arabia. *SMJ* 2016; **37**: 280–287.

293 Farman M, Yasir M, Al-Hindi RR *et al.* Genomic analysis of multidrug-resistant clinical Enterococcus faecalis isolates for antimicrobial resistance genes and virulence factors from the western region of Saudi Arabia. *Antimicrob Resist Infect Control* 2019; **8**: 55.

294 Kankalil George S, Suseela MR, El Safi S *et al.* Molecular determination of van genes among clinical isolates of enterococci at a hospital setting. *Saudi Journal of Biological Sciences* 2021; **28**: 2895–2899.

295 Albert MJ, Bulach D, Alfouzan W *et al.* Non-typhoidal Salmonella blood stream infection in Kuwait: Clinical and microbiological characteristics. *PLoS Negl Trop Dis* 2019; **13**: e0007293.

296 Garaween G, Somily A, Raji A *et al.* Serogenotyping and emergence of extended-spectrum β-lactamase genes in non-typhoidal Salmonella: first report from Saudi Arabia. *Journal of Medical Microbiology* 2016; **65**: 1343–1346.

297 Alsalem Z, Elhadi N, Aljeldah M, Alzahrani F, Nishibuchi M. Characterization of Vibrio vulnificus Isolated from the Coastal Areas in the Eastern Province of Saudi Arabia. *J Pure Appl Microbiol* 2018; **12**: 1355–1364.

298 M Kurdi Al-Dulaimi M, Abd Mutalib S, Abd Ghani M, Mohd Zaini NA, Ariffin AA. Multiple Antibiotic Resistance (MAR), Plasmid Profiles, and DNA Polymorphisms among Vibrio vulnificus Isolates. *Antibiotics (Basel)* 2019; **8**: E68.

299 Abro AH. Salmonella typhi: Antibiotic sensitivity pattern in Dubai, United Arab Emirates. *Pakistan Journal of Medical Sciences* 2011; **27**: 6.

300 AL-Fatlawy HNK, AL-Hadrawi HAN. Molecular Profiling of Class I Integron Gene in MDR Salmonella typhi Isolates. *J Pure Appl Microbiol* 2020; **14**: 1825–1833.

301 John Albert M, Bulach D, Alfouzan W *et al.* Non-typhoidal Salmonella blood stream infection in Kuwait: Clinical and microbiological characteristics. *PLoS Neglected Tropical Diseases* 2019; **13**: 1–21.

302 Abduljabbar A, Aljanaby J. Antibacterial Activity Of An Aqueous Extracts Of Alkanna Tinctoria Roots Against Drug Resistant Aerobic Pathogenic Bacteria Isolated From Patients With Burns Infections. *Russian Open Medical Journal* 2018; **7**: e0104.

303 Yassin MT, Mostafa AA, Al-Askar AA, Bdeer R. In vitro antifungal resistance profile of Candida strains isolated from Saudi women suffering from vulvovaginitis. *Eur J Med Res* 2020; **25**: 1.

304 Majeed HT, Aljanaby AAJ. Antibiotic Susceptibility Patterns and Prevalence of Some Extended Spectrum Beta-Lactamases Genes in Gram-Negative Bacteria Isolated from Patients Infected with Urinary Tract Infections in Al-Najaf City, Iraq. *Avicenna J Med Biotechnol* 2019; **11**: 192–201.

305 Alaboudi AR, Malkawi IM, Osaili TM, Abu-Basha EA, Guitian J. Prevalence, antibiotic resistance and genotypes of Campylobacter jejuni and Campylobacter coli isolated from chickens in Irbid governorate, Jordan. *International Journal of Food Microbiology* 2020; **327**: 108656.

306 Ghunaim H, Behnke JM, Aigha I *et al.* Analysis of Resistance to Antimicrobials and Presence of Virulence/Stress Response Genes in Campylobacter Isolates from Patients with Severe Diarrhoea. *PLoS ONE* 2015; **10**: e0119268.

307 Kanaan MHG, Mohammed FA. ANTIMICROBIAL RESISTANCE OF CAMPYLOBACTER JEJUNI FROM POULTRY MEAT IN LOCAL MARKETS OF IRAQ. ; : 7.

308 Jasim SA, Al-abodi HR, Ali WS. Resistance rate and novel virulence factor determinants of Arcobacter spp., from cattle fresh meat products from Iraq. *Microbial Pathogenesis* 2021; **152**: 104649.

309 AbdAlhussen LS, Darweesh MF. Prevelance and antibiotic susceptibility patterns of Pantoea spp. *International Journal of ChemTech Research* 2016; : 9.

310 Fazaa SA, Darweesh MF. Prevalence of Pantoea spp. among recurrent UTI patients with emphasis on risk factors and antibiotic resistance patterns in Al-Qadisiyah hospitals, Iraq. *ATMPH* 2020; **23**. doi:10.36295/ASRO.2020.231202.

311 Burjaq SZ, Abu-Romman SM. Prevalence and Antimicrobial Resistance of Salmonella spp. From Irrigation Water in Two Major Sources in Jordan. *Curr Microbiol* 2020; **77**: 3760–3766.

312 Jassim AA, Al-Gburi NM. VIRULENCE GENES AND ANTIMICROBIAL RESISTANCE OF SALMONELLA ISOLATED FROM MILK IN WASIT PROVINCE, IRAQ. ; : 8.

313 Olaitan AO, Dia NM, Gautret P *et al.* Acquisition of extended-spectrum cephalosporin- and colistin-resistant Salmonella enterica subsp. enterica serotype Newport by pilgrims during Hajj. *International Journal of Antimicrobial Agents* 2015; **45**: 600–604.

314 Osaili TM, Al-Nabulsi AA, Shaker RR *et al.* Prevalence of Salmonella Serovars, Listeria monocytogenes, and Escherichia coli O157:H7 in Mediterranean Ready-to-Eat Meat Products in Jordan. *Journal of Food Protection* 2014; **77**: 106–111.

315 Abd Al-Mayahi FS, Jaber SM. Multiple drug resistance of Listeria monocytogenes isolated from aborted women by using serological and molecular techniques in Diwaniyah city/ Iraq. *IJM* 2020. doi:10.18502/ijm.v12i4.3933.

316 Ahmed MS, Taha ZMA, Omer LT. Isolation and Molecular Identification with Resistant Profile Determination of Listeria monocytogenes from Imported Chicken Carcasses in Duhok, Kurdistan Region, Iraq. *Journal of Pure and Applied Microbiology* 2015; **9**: 97–103.

317 Alanber MN, Alharbi NS, Khaled JM. Evaluation of multidrug-resistant Bacillus strains causing public health risks in powdered infant milk formulas. *Journal of Infection and Public Health* 2020; **13**: 1462–1468.

318 Thualfakar Hayder, Aljanaby. Antibiotics susceptibility patterns of Citrobacter freundii isolated from pa-tients with urinary tract infection in Al-Najaf governorate – Iraq. *ijrps* 2019; **10**: 1481–1488.

319 Abd El Hafez M, Khalaf NG, El Ahmady M, Abd El Aziz A, Hashim AEG. An outbreak of methicillin resistant Staphylococcus epidermidis among neonates in a hospital in Saudi Arabia. *J Infect Dev Ctries* 2011; **5**: 692–699.

320 Talat A, Khalid S, Majeed HAR, Khan AU. Whole-genome sequence analysis of multidrug-resistant Staphylococcus epidermidis ST35 strain isolated from human ear infection of an Iraqi patient. *Journal of Global Antimicrobial Resistance* 2020; **21**: 318–320.

321 Al-Muhanna AS, Al-Muhanna S, Alzuhairi MA. Molecular investigation of extended-spectrum beta-lactamase genes and potential drug resistance in clinical isolates of *Morganella morganii*. *Annals of Saudi Medicine* 2016; **36**: 223–228.

322 M. Kurdi Al-Dulaimi M, Abd. Mutalib S, Abd. Ghani M, Mohd. Zaini NA, Ariffin AA. Multiple Antibiotic Resistance (MAR), Plasmid Profiles, and DNA Polymorphisms among Vibrio vulnificus Isolates. *Antibiotics* 2019; **8**: 68.

323 Hanan ZK. Molecular Detection of Cholera Infection during the Outbreak in Thi-Qar Province/Iraq in 2015-2016. *J Phys: Conf Ser* 2019; **1279**: 012068.

324 AlJindan R, AlEraky DM, Borgio JF *et al.* Diagnostic deficiencies of C. difficile infection among patients in a tertiary hospital in Saudi Arabia: A laboratory-based case series. *Saudi Journal of Biological Sciences* 2021; : S1319562X21003053.

325 Al-Sa'ady AT. Detection of vancomycin resistance in multidrug-resistant Enterococcus faecalis isolated from burn infections. *Drug Invention Today* 2019; **11**: 7.

326 Ulger Toprak N, Veloo ACM, Urban E *et al.* A multicenter survey of antimicrobial susceptibility of Prevotella species as determined by Etest methodology. *Anaerobe* 2018; **52**: 9–15.

327 Taj-Aldeen SJ, Deshmukh A, Doiphode S *et al.* Molecular Identification and Susceptibility Pattern of Clinical *Nocardia* Species: Emergence of *Nocardia crassostreae* as an Agent of Invasive Nocardiosis. *Canadian Journal of Infectious Diseases and Medical Microbiology* 2013; **24**: e33–e38.

328 Taj-Aldeen SJ, Shamseldin Elshafie S. Emerging resistant serotypes of invasive Streptococcus pneumoniae. *IDR* 2016; **Volume 9**: 153–160.

329 El-Mahdy TS, Al-Agamy MH, Al-Qahtani AA, Shibl AM. Detection of *bla* $_{\text{OXA-23-like}}$ and *bla* $_{\text{NDM-1}}$ in *Acinetobacter baumannii* from the Eastern Region, Saudi Arabia. *Microbial Drug Resistance* 2017; **23**: 115–121.

330 Khan Z, Ahmad S, Joseph L, Al-Obaid K. Isolation of cholesterol-dependent, multidrug-resistant Candida glabratastrains from blood cultures of a candidemia patient in Kuwait. *BMC Infect Dis* 2014; **14**: 188.

331 Alosaimi RS, Muhmmed kaaki M. Catheter-Related ESBL-Producing *Leclercia adecarboxylata* Septicemia in Hemodialysis Patient: An Emerging Pathogen? *Case Reports in Infectious Diseases* 2020; **2020**: 1–3.

332 Guan Q, Almutairi TS, Alhalouli T, Pain A, Alasmari F. Metagenomics of Imported Multidrug-Resistant *Mycobacterium leprae* , Saudi Arabia, 2017. *Emerg Infect Dis* 2020; **26**: 615–617.

333 Abdalhamid B, Elhadi N, Alsamman K, Aljindan R. Chryseobacterium gleum pneumonia in an infant with nephrotic syndrome. *IDCases* 2016; **5**: 34–36.
